# HPV knowledge and vaccine acceptance among European adolescents and their parents: a systematic literature review

**DOI:** 10.1186/s40985-020-00126-5

**Published:** 2020-05-14

**Authors:** Noelia López, Maria Garcés-Sánchez, Maria Belén Panizo, Ignacio Salamanca de la Cueva, Maria Teresa Artés, Beatriz Ramos, Manuel Cotarelo

**Affiliations:** 1 Medical Affairs Department, Merck Sharp & Dohme Spain, Madrid, Spain; 2 Nazaret Healthcare Center, Valencia, Spain; 3 Illescas Healthcare Center, Toledo, Spain; 4 Instituto Hispalense de Pediatría, Sevilla, Spain; 5 Adelphi Spain, Barcelona, Spain

**Keywords:** HPV, Papillomavirus, Vaccination, Acceptance, Adolescent, Knowledge

## Abstract

**Background:**

Since the introduction of HPV vaccines, several studies have been conducted in different countries to assess HPV knowledge and vaccine acceptance. The aim of this study was to perform a systematic literature review to summarize results and identify factors associated with HPV knowledge and vaccine acceptance in adolescents and their parents and to compile the measurement tools used in the published research studies performed in European countries where HPV is licensed.

**Methods:**

A systematic literature review was conducted for studies published between January 1st 2006 and December 31st 2017.

**Results:**

Seventy non-interventional studies performed in 16 European countries met the inclusion criteria. Thirty-eight of them reported data on HPV knowledge and 40 reported data on HPV vaccine acceptance. Further, 51.8% of adolescents (range 0% to 98.6%) and 64.4% of parents (range 1.7% to 99.3%) knew about HPV infection. Insufficient information and safety concerns were the main barriers to vaccination acceptance.

**Conclusion:**

HPV knowledge and vaccine acceptance are still modest and vary widely between studies across EU countries. Coordinated efforts should be made to provide the relevant population with information for informed decision-making about HPV vaccination.

## Background

Human papillomavirus [HPV] infection is one of the major causes of infection-related cancer worldwide and is the causal factor in other diseases such as genital warts or recurrent respiratory papillomatosis [1]. More than 200 HPV types have been already sequenced. According to the International Agency for Research on Cancer, high-risk HPV genotypes including 16, 18, 31, 33, 45, 52, and 58 are responsible for around 90% of anogenital HPV-positive cancers worldwide, whereas HPV 6 and 11, low-risk genotypes, are responsible for 90% of genital warts [2]. Apart from anogenital cancers, HPV is known to be responsible for a variable fraction of head and neck cancers [3].

Approximately, 80% of sexually active individuals will be infected by HPV during their lifetime [4]. Most of these infections are immunologically controlled within 1–2 years. However, if the infection persists, it can cause cellular changes that can lead to certain types of cancers. According to the latest data reported for Europe, an estimated 680,344 to 844,391 genital warts; 216,636 to 413,977 cases of high-grade cervical intraepithelial lesions (CIN2+); 31,130 cervical cancer cases; 6786 head and neck cancers; and 10,076 cancers in vulva, vagina, penis, and anus attributable to the aforementioned nine HPV types [2] are diagnosed annually in males and females. Some of these conditions, such as anal or oropharyngeal cancer, have increased recently [5].

Currently, there are three licensed HPV vaccines in the European Union (EU): a bivalent, including HPV types 16 and 18, approved in 2006; a tetravalent, including HPV types 6, 11, 16, and 18, authorized in 2006; and a nonavalent vaccine, including HPV 6, 11, 16, 18, 31, 33, 45, 52 and 58 that was licensed in 2015 [6–8]. According to the European Centre for Disease Prevention and Control (ECDC), by 2012, 19 European countries had introduced HPV vaccination in females, and ten of them had organized catch-up programs [9]. More recently, certain countries have extended HPV vaccination to males within their immunization programs. Up to 27 countries globally, 13 of them in Europe have implemented gender-neutral vaccination programs [10].

Despite these advances, coverage rates of HPV vaccination programs differ widely between countries [9]. To address this, in May 2017, the World Health Organization (WHO) underlined the importance of cervical cancer and other HPV-related diseases as a global public health problem and reiterated its recommendation to include HPV vaccines in national immunization programs as part of a coordinated and comprehensive strategy to prevent HPV-related diseases [11].

Since most HPV vaccination programs target mainly young adolescents, parents have the authority to take most decisions about vaccination. Therefore, the success of HPV vaccination programs will largely depend on parental decision-making [12]. A comprehensive model to explain vaccine hesitancy among parents was defined by Dubé et al. [13] including a number of factors at the individual level: knowledge and information, past experiences, perceived importance of vaccination, risk perception and trust, subjective norm, religious and moral convictions but also the historical, political, and socio-cultural context, public health policies, health professionals recommendations, and media influence.

The concept of knowledge commonly includes the awareness about “who, where, and when” one should be vaccinated and self-estimated sufficiency of information about vaccination or satisfaction with information on vaccination [13]. While acceptability is a more complex multi-faceted construct that reflects the extent to which people delivering or receiving a healthcare intervention consider it to be appropriate, based on anticipated or experienced cognitive and emotional responses to the intervention [14]. Several theoretical models of acceptability have been proposed in the literature. The 5C model [15] describes five relevant psychological antecedents of vaccination: confidence, complacency (risk perceptions), constraints (barriers), calculation (extent of information search), and collective responsibility (willingness to protect the community).

Since the introduction of HPV vaccines, several studies have been conducted in different countries to assess HPV knowledge and vaccine acceptance.

At the end of 2017, two systematic reviews explored factors related to the uptake of vaccination programs in the EU [16, 17]. The first one [16] examined worldwide HPV vaccination uptake and associated factors; however, studies not reporting HPV vaccination coverage rates were excluded. The second study [17] assessed parental attitudes toward HPV vaccination in male children; no data about female vaccination were included. Consequently, there is no comprehensive systematic literature review summarizing factors influencing HPV knowledge and vaccine acceptance among adolescents and their parents since the introduction of HPV vaccines until now in EU countries. Moreover, to our knowledge, no publication has consistently compiled measurement tools used in published studies that assessed HPV knowledge and vaccine acceptance. This compilation could be useful for new researchers in this field.

The aim of this study was to perform a systematic literature review to identify factors associated with HPV knowledge and vaccine acceptance in adolescents and their parents, to summarize the results for both outcomes, and to compile the measurement tools, items, and questionnaires used in published research studies performed in European countries where HPV vaccines are licensed.

## Methods

We reviewed all the scientific literature published between January 1st, 2006 and December 31st, 2017 to identify studies evaluating parental and/or adolescent HPV knowledge and/or acceptance of HPV vaccination. Our search was limited to studies targeting populations from European countries where HPV vaccines were licensed when this protocol was written to ensure homogeneity as far as common regulation (European Medicines Agency, EMA) among the included countries and common vaccination recommendations (European Centre for Disease Prevention and Control, EDCD) (Austria, Belgium, Bulgaria, Croatia, Cyprus, Czech Republic, Denmark, Estonia, Finland, France, Germany, Greece, Hungary, Iceland, Ireland, Italy, Latvia, Liechtenstein, Lithuania, Luxemburg, Macedonia, Netherlands, Norway, Portugal, Serbia, Slovenia, Spain, Sweden, Switzerland, and the UK).

### Inclusion and exclusion criteria

Studies whose primary outcomes were HPV knowledge and/or acceptance of HPV vaccination were included. Following PICOTS, we defined the following criteria for study selection:

- Population: only studies performed in parents of children of any age under 19 years or an adolescent population defined as individuals aged 9–18 years old living in a European country where HPV vaccines were licensed were included.
- Intervention: not applicable.
- Comparator: results regarding HPV knowledge and acceptability of HPV vaccination were recalled, whenever possible by sex of the respondent, sex of the target child, and country.
- Outcomes: a study was regarded as measuring knowledge of HPV if it assessed a set of true/false, yes/no or any other format of questions that could be translated into a scoring system showing the knowledge and understanding of how the virus is spread, what conditions result from HPV infections and how HPV can be prevented. A study was regarded as measuring acceptance of HPV vaccination whenever it somehow evaluated a positive or negative intention or willingness toward vaccinating children (girls or boys) or oneself (in the case of adolescents) in the future (vaccine intention); or having consented or not to vaccination their children or oneself (in the case of adolescents) in the past. In addition, drivers for accepting HPV vaccination and barriers and reasons to refuse it were recorded and analyzed.
  Study design: quantitative survey research studies published as original articles were included. Reviews, editorials, and gray literature (dissertations, conference abstracts, trial registries, pharmaceutical company databases, etc.) were not included in the search.
- Time: only studies published in English during the last 11 years were included (2006–2017).

### Search strategy

The search strategy used appropriate keywords, medical subject heading, and free-text terms for the following concepts: “human papillomavirus AND [survey OR questionnaire OR assessment] AND [knowledge OR acceptance OR attitudes] AND vaccine.”

A combination of text words and MeSH Terms was defined with a medical librarian and after several preliminary manual test searches.

The following principal sources of electronic reference libraries were searched to access the available data: The Cochrane Library, Medline through PubMed, EMBASE, and Popline. An exploratory search in Google and Google Scholar was also made to avoid any publication bias.

We also included the World Bank Library Network, although we did not obtain relevant publications from this source.

The list of search queries used per bibliographic source is provided in the Supplementary Material.

### Study selection

The titles of all studies identified were screened independently by two reviewers and duplicates were removed. Titles were screened for inclusion and abstracts were further reviewed based on eligibility criteria. Any disagreements on selection of studies between the two primary reviewers were resolved by an expert committee comprised of four expert pediatricians in HPV and vaccination (MGS, DMP, MBO, and ISC). Following retrieval of the full texts of all the studies that met the inclusion/exclusion criteria, data confirming these criteria were extracted from each study by the two reviewers on a standardized abstraction sheet. Any disagreement on the final selection of studies to be included in the review was resolved by the expert committee.

### Assessment of study quality in included studies

Study quality was assessed by two independent reviewers using the Mixed Method Assessment Tool (MMAT) developed by Pluye et al. [18] (see Supplementary Material) to identify factors that might have introduced bias or limited the generalizability of the results. In case of non-concordance, the expert committee also assessed the quality of the non-concordant studies and a consensus decision was taken by the two reviewers and the expert committee. Studies showing a MMAT score < 50 were excluded.

### Data collection

Data extraction was performed by the lead reviewer using a bespoke form. An additional reviewer checked a random sample of 15% of the data records to detect possible extraction errors.

The form’s suitability was assessed by performing a pilot extraction of three studies selected on the basis of the diversity of their content and design. Both the lead reviewer and the additional reviewer performed the pilot extraction independently. Results and completion difficulties were subsequently compared to improve the questionnaire accordingly. The final data extraction form used is included in the Supplementary Material.

### Data analysis

Items were classified under headings and subheadings based on their conceptual meaning. Main headings were knowledge about HPV, knowledge about HPV vaccine, acceptability of vaccines in general, and acceptability of HPV vaccine. The complete list of subheadings defined under each heading is shown in first column of Table 2.

For each individual item reported by the studies included, the literal item text, the reported number, and percentage of participants choosing the response answer that implies knowledge (yes, true, correct…) and/or acceptability (yes, positive, agreement…), respectively was recorded in a database.

For each heading and subheading, the number of items reporting data, sum of the studies’ sample size, sum of the number of participants answering each item as previously defined, computed pooled percentage (based on the two previous frequency values), arithmetic mean of the percentages of the items included in the heading, minimum reported percentage, and maximum reported percentage for each conceptual heading in the original study were obtained or calculated.

To collect the information on factors associated with HPV knowledge and/or HPV vaccine acceptance, reviewers extracted the data of odds ratio, beta coefficients, or *p* values (depending on availability) reported in the included studies that presented a statistical association with HPV knowledge and/or with the acceptance of HPV vaccination. Total number of studies studying each factor is reported and whether the analysis of the relationship was multivariate or bivariate.

## Results

A total of 2118 publications were identified: 609 were retrieved from PubMed, 206 from Cochrane Library, 1124 from EMBASE, 141 from the World Bank Library, and 38 from Popline. The exploratory search in Google and Google Scholar did not yield any new peer-reviewed publication not already included.

Duplicates were excluded and 1666 publications were retained for title and abstract-based screening. Of these, 1516 were excluded according to selection criteria. Only 150 were selected for full-text review. Eleven of them were excluded because the aim of the study was not consistent with the objective of this systematic review, ten were not original papers providing primary data, one was conducted in a country where HPV vaccines were not commercialized, 18 did not provide segregated data for the target population of this review or were conducted in a different target population, 31 studies were performed in non-European countries, four were published in a language other than English, and three were additional duplicates.

**Fig. 1:**
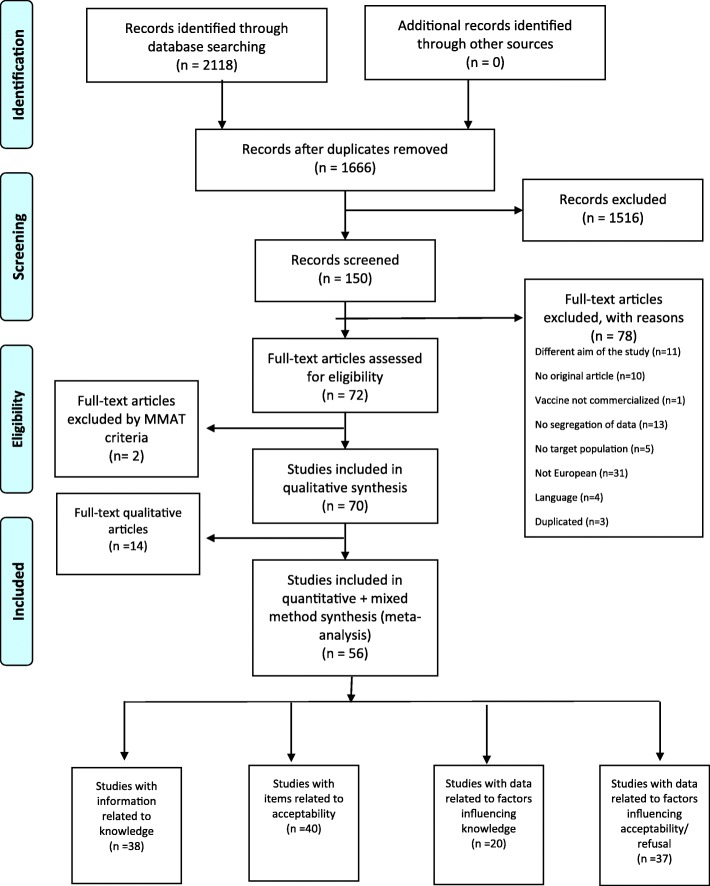
PRISMA flow diagram

Thus, 72 studies that met the inclusion criteria were finally included, although two had to be excluded after quality evaluation with MMAT. Ultimately, 70 publications were included for further analysis. See PRISMA flow diagram (Fig. 1), list of excluded studies and reason for exclusion in Supplementary Material.

All 70 publications were non-interventional studies, 14 were qualitative studies and were therefore not included in the quantitative synthesis, although they were used to guide the conceptual framework to classify the items of quantitative studies under headings and subheadings. Items used across studies to measure this knowledge were divided into two main headings: knowledge of HPV and knowledge of HPV vaccine. We also included a third heading for “other” information, such as knowledge of sexually transmitted infections (STI). Items related to information source were also classified (see Supplementary Material).

Thirty-eight publications reported data about HPV knowledge and 40 about HPV vaccine acceptance. Of them, 20 and 37 studies, respectively, reported quantitative results about factors influencing HPV knowledge and HPV vaccine acceptance.

In terms of geographical distribution, publications included in this review were produced in 16 European countries, the UK having most publications, 20 (28.6%), followed by Italy, ten (14.3%), and Sweden, eight (11.4%). There was only one pan-European study involving four countries: France, the UK, Germany, and Italy.

Twenty-nine studies were conducted on adolescents aged between 9 and 21 years (15 in females, one in males, and 13 in both genders); 36 were conducted in parents of adolescents (12 in mothers only and 24 in in both parents). A summary of the characteristics of studies included in the systematic review is provided in Table 1.

**Table 1:** Summary of characteristics of studies included in the systematic review

Author	Year of publication	Country	Interviewed population	Study setting	Sample size	Study aim	Study design	MMAT score
Alberts CJ et al. [19]	2017	Netherlands	Parents (mother and father) of female children 13 y.o.	Regional sample	1257	To explore the possible impact of ethnicity on the determinants of both HPV vaccination intention and HPV vaccination uptake among parents/guardians having a daughter to whom the HPV vaccination is proposed.	Quantitative non-randomized	75
Anagnostou PA et al. [20]	2017	Greece	Adolescents (male and female) 12–18 y.o.	National sample	268	(1) To develop an instrument to assess knowledge of HPV and its vaccine and utilize this instrument to measure knowledge levels of Greek adolescents in Lyceum schools of Western Thessaloniki; and (2) to examine the associations of the resulting knowledge measure scores with sociodemographic characteristics.	Quantitative non-randomized	75
Grandahl M et al. [21]	2017	Sweden	Parents (mother and father) of female children 11–12 y.o.	Regional sample	366	To examine the association between parents’ refusal and sociodemographic background, knowledge and beliefs about HPV and the HPV vaccination in relation to the Health Belief Model.	Quantitative non-randomized	50
Grandahl M et al. [22]	2017	Sweden	Adolescents (male and female) 16 y.o.	National sample	751	To examine HPV catch-up vaccination status in adolescents in relation to (1) socioeconomic factors, (2) beliefs and knowledge about HPV prevention, and (3) sexual behavior.	Quantitative non-randomized	100
Navarro-Illana P et al. [23]	2017	Spain	Female adolescents and parents of female children 12–16	Regional sample	833	To describe the drivers associated with HPV vaccination in adolescent girls and their parents’ opinion about the vaccine.	Quantitative non-randomized	100
Patel H et al.et al [24]	2017	Latvia	Adolescents (male and female) 18 (16–21) y.o.	Regional sample	121	To evaluate awareness of HPV and its vaccine among Latvian adolescents.	Quantitative non-randomized	50
Vaidakis D et al. [25]	2017	Greece	Adolescents (male and female) 17–18 y.o.	National sample	4507	To identify sexual behaviour, attitudes, beliefs and knowledge of sexually transmitted infections (STIs) focused on HPV in the Greek adolescent population.	Quantitative non-randomized	100
Balla B et al. [26]	2016	Hungary	Female adolescents 18–19 y.o.	Regional sample	492	To explore the attitudes towards the HPV vaccine and the knowledge of cervical cancer among senior high-school girls in Budapest.	Quantitative non-randomized	50
Borena W et al. [27]	2016	Austria	Parents (mother and father) of children (male and female) 9–10 y.o.	National sample	439	To assess parental factors associated with decision to let children receive gender-neutral, free of- charge, school-based HPV immunization.	Quantitative non-randomized	100
Oddsson K et al. [28]	2016	Iceland	Parents (mother and father) of female children 12 y.o.	National sample	583	To assess attitude and knowledge among parents or guardians of 12 y.o. girls about HPV, cervical cancer and HPV vaccination.	Quantitative non-randomized	75
Schülein S et al. [29]	2016	Germany	Female adolescents 9–17 y.o.	National sample	2,224	To determine HPV vaccine uptake as well as factors associated with uptake in 9 to 17-year-old girls in Germany during the first year of vaccine availability.	Quantitative non-randomized	75
Voidăzan S et al. [30]	2016	Romania	Parents (mother and father) of children (male and female) 7–10 y.o.	Regional sample	918	To evaluate the level of parental knowledge about HPV infection and HPV vaccination including the information obtained from general practitioners and identification of barriers in implementing a vaccination strategy.	Quantitative non-randomized	100
Agorastos T et al. [31]	2015	Greece	Mothers of children (male and females) 13 y.o.	National sample	10,758	To investigate the possible effect of demographic factors on HPV vaccination acceptance in Greece.	Quantitative non-randomized	25
Firenze A et al. [32]	2015	Italy	Female adolescents 15 (13–17) y.o.	Regional sample	378	To evaluate knowledge and factors associated with HPV unvaccinated girls after five years of vaccination program implementation in Sicily, an Italian region with low vaccination coverage (< 50.0%).	Quantitative non-randomized	50
Forster A et al. [33]	2015	United Kingdom	Female adolescents 15–16 y.o.	Regional sample	2163	To explore reasons for being un-/under vaccinated.	Quantitative non-randomized	100
La Torre G et al. [34]	2015	Italy	Mothers of female children under and over 18 y.o.	Regional sample	444	To evaluate the knowledge and attitudes of Italian mothers—whose daughters had been vaccinated in 2012—towards primary (anti-HPV vaccination) and secondary (Pap test screening) cervical cancer prevention, as well as sources of information and mother-daughter communication on health issues.	Quantitative non-randomized	50
Lee Mortensen G et al. [35]	2015	UK, France, Germany, and Italy.	Parents (mother & father) of male children 12–17 y.o.	Multinational sample	1,837	To examine parental attitudes to HPV vaccination of their sons given brief information about HPV	Mixed Method	75
Maier C et al. [36]	2015	Romania	Adolescents (male and female) 16–18 y.o.	Regional sample	524	To explore the barriers to HPV vaccination with a view to developing strategies for expanding primary HPV infection prevention.	Quantitative non-randomized	50
Navarro-Illana P et al. [37]	2015	Spain	Mothers of female children 12–16 y.o.	Regional sample	833	To assess knowledge about HPV infection and its vaccine among the mothers of girls and to identify factors associated with the willingness to vaccinate their daughters.	Quantitative non-randomized	100
Bianco AS et al. [38]	2014	Italy	Parents (mother and father) of male children 10–14 y.o.	National sample	1021	To elicit information about parents’ knowledge, attitudes, and acceptability toward HPV infection and vaccination of male adolescents in Italy; to identify subgroups of this population who exhibit poor knowledge about prevention of HPV infection and reveal negative attitudes toward HPV vaccination in relation to their male sons.	Quantitative non-randomized	75
Bowyer HL et al. [39]	2014	United Kingdom	Female adolescents 16–17 y.o.	Regional sample	650	To examine psychosocial predictors of HPV vaccine uptake and the association between vaccine intention and uptake 1 year later in adolescent girls (aged 16–17 years) in England.	Quantitative non-randomized	100
Giambi C et al. [40]	2014	Italy	Parents (mother and father) of female children 12–14 y.o.	National sample	1738	To explore reasons for non-vaccination HPV in Italy.	Quantitative non-randomized	50
Mollers M et al. [41]	2014	Netherlands	Female adolescents 16–17 y.o.	National sample	2989	To explore differences between vaccinated and unvaccinated girls with regards to characteristics such as education, ethnicity, (sexual) risk behaviour and knowledge of HPV. Understanding the features of these two groups could provide insight in future vaccine and screening targeting efforts.	Quantitative non-randomized	75
Navarro - Illana P et al. [42]	2014	Spain	Female adolescents 15 y.o.	Regional sample	833	To assess the knowledge and attitudes of 15-year-old Spanish girls (who were candidates to receive the vaccine) towards HPV infection and the vaccine, and to identify independently associated factors that could potentially be modified by an intervention.	Quantitative non-randomized	100
Wegwarth O. et al. [43]	2014	Germany	Parents (mother and father) of female children 12–17 y.o.	Regional sample	225	To learn how balanced versus unbalanced information about HPV vaccination influences (1) girls’ and parents’ knowledge of the risk of cervical cancer and the effectiveness of the HPV vaccine (both being the basis for informed decisions), (2) their perceived risk of developing cervical cancer without having the HPV vaccine, (3) the intention to have the vaccine, (4) the actual vaccination decision, and (5) the phenomenon of the “knowledge–behavior gap”	Quantitative non-randomized	100
Bowyer HL et al. [44]	2013	United Kingdom	Female adolescents 15–16 y.o.	Regional sample	1033	To examine knowledge about HPV and the HPV vaccine, in girls based in London, England, three years after the introduction of routine school-based vaccination.	Quantitative non-randomized	100
Hofman R et al. [45]	2013	Netherlands	Female adolescents 11–14 y.o.	Regional sample	237	To evaluate to what extent reading an official information leaflet about HPV contributes to girls’ knowledge levels, and to what extent an increase in knowledge is boosted by a pre-test measurement.	Quantitative randomized controlled	75
Stöker P et al. [46]	2013	Germany	Female adolescents 15 (14–18) y.o.	Regional sample	476	To assess HPV-vaccination coverage and knowledge among students in Berlin, to identify factors influencing HPV-vaccine uptake.	Quantitative non-randomized	75
Sopracordevole F et al. [47]	2013	Italy	Adolescents (male and female) 16 (13–20) y.o.	Regional sample	1105	To assess the knowledge of teenage girls on HPV infection and vaccination 12 months after the start of a vaccine administration and information campaign.	Quantitative non-randomized	100
Tisi G et al. [48]	2013	Italy	Parents (mother and father) of male children 11–15 y.o.	Regional sample	161	To evaluate the comprehension and acceptance of HPV vaccination in parents of adolescent boys aged 11 to 15 years.	Quantitative non-randomized	75
van Keulen H et al. [49]	2013	Netherlands	Female adolescents and Mothers of female adolescents 13–14 y.o.	National sample	1594	To examine the social and psychological determinants of the HPV vaccination intentions of girls aged 13 to 16 years and their mothers who were targeted by the Dutch catch-up campaign of 2009.	Quantitative non-randomized	75
Forster A et al. [50]	2012	United Kingdom	Male adolescents 16–18 y.o.	Regional sample	528	To assess boys’ willingness to have HPV vaccination, eliciting reasons for their decisions.	Quantitative randomized controlled	75
Gefenaite G et al. [51]	2012	Netherlands	Parents (mother and father) of female children 13–16 y.o.	Regional sample	609	To identify the most important determinants of refusing the vaccination.	Quantitative non-randomized	75
Haesebaert J et al. [52]	2012	France	Mothers of female children 14–18 y.o.	Regional sample	210	To assess knowledge about cervical cancer, the Pap test and HPV vaccination in 18–65-year-old French women one year after the introduction of the vaccine. The second objective was to assess mothers’ acceptance of HPV vaccination for their 14–18-year-old daughters and determinants of that acceptability.	Mixed Method	60
Marek E et al. [53]	2012	Hungary	Adolescents (male and female) 14–19 y.o.	Regional sample	394	To explore the impact of a brief, HPV-focused program on adolescents’ knowledge, beliefs and attitudes.	Quantitative non-randomized	100
Samkange-Zeeb F et al. [54]	2012	Germany	Female adolescents 12–20 y.o.	Regional sample	632	To assess awareness of HPV and of vaccination status among girls attending grades 8–13 in Bremen and Bremerhaven, two German cities.	Quantitative non-randomized	75
Sopracordevole F et al. [55]	2012	Italy	Adolescents (male and female) 16 y.o.	Regional sample	1105	To assess teens’ knowledge of HPV infection and vaccination one year after the initiation of the public vaccination programme and information campaign on the disease and the opportunity of vaccination.	Quantitative non-randomized	75
Balemans R et al. [56]	2011	Belgium	Adolescents (male and female) 14-17 y.o.	Regional sample	186	To investigate knowledge among adolescents in Antwerp about vaccination and to describe their information sources, motives and barriers for vaccination.	Quantitative non-randomized	75
Chadenier GMC et al. [57]	2011	Italy	Mothers of female children 12 y.o.	Regional sample	475	To observe the coverage of the first vaccination campaign in two suburbs of Milan, to assess knowledge about HPV and cervical cancer among mothers of recipients, and to collect opinions of healthcare professionals involved in the organization of the campaign.	Quantitative non-randomized	100
Marek E t al [58].	2011	Hungary	Adolescents (male & female) 12-19 y.o.	National sample	1,769	To determine factors and motivations affecting the uptake of HPV vaccination among Hungarian adolescents.	Quantitative non-randomized	100
Dahlström L et al. [59]	2010	Sweden	Parents (mother and father) of children (male and female) 12–15 y.o.	National sample	20,000	To examine Swedish parents’ perceptions and concerns about HPV vaccination, their willingness to vaccinate their children against HPV when the vaccine is free or not and correlates of acceptability of the new HPV vaccine.	Quantitative randomized controlled	100
Lee Mortensen G et al. [60]	2010	Denmark	Parents (mother and father) of male children 12–15 y.o.	Regional sample	450	To assess parental attitudes to HPV vaccination of their sons.	Quantitative non-randomized	75
Morison L et al. [61]	2010	United Kingdom	Parents (mother and father) of female children 11–12 y.o.	Regional sample	245	To evaluate the role of temporal perspective in the formation of attitudes and intentions towards the vaccine.	Mixed Method	67
Pelucchi C et al. [62]	2010	Italy	Adolescents (male and female) and parents 14–19 y.o.	Regional sample	858 and 2331	To provide data on the knowledge of Italian adolescents and parents concerning HPV infection and its prevention in order to allow the development of adequate training programmes.	Quantitative non-randomized	100
Gottvall M et al. [63]	2009	Sweden	Adolescents (male and female) 15–16 y.o.	Regional sample	608	To investigate knowledge of HPV and attitudes to HPV vaccination and condom use among Swedish first year upper secondary school students.	Quantitative non-randomized	100
Höglund A et al. [64]	2009	Sweden	Adolescents (male and female) 16 (15–20) y.o.	Regional sample	459	To investigate knowledge of and attitudes to sexually transmitted infection (STI) and STI prevention with special focus on HPV and the vaccine against HPV, among 16-year-old high school students in a Swedish context.	Quantitative non-randomized	100
Marlow L et al. [65]	2009	United Kingdom	Female adolescents 16–19 y.o.	Regional sample	335	To assess acceptability of HPV vaccination among female adolescents (16–19 years) and investigate socio-cultural variation in intended acceptance.	Quantitative non-randomized	100
Tozzi A et al .[66]	2009	Italy	Mothers of female children 10–12 y.o.	Regional sample	1,007	To assess parents’ knowledge about HPV and HPV vaccination and their willingness to have their daughters immunized, and to investigate the roles of the different medical specialists in the immunization strategy as perceived by parents.	Quantitative non-randomized	75
De Visser R et al. [67]	2008	United Kingdom	Parents (mother and father) of children (male and female) 12-13 y.o.	Regional sample	353	To examine how intentions to vaccinate against HPV are influenced by general beliefs about vaccination, specific beliefs about HPV vaccination, knowledge about HPV and cervical cancer, and beliefs about adolescent sexual behavior.	Quantitative non-randomized	75
Lenselink C et al. [68]	2008	Netherlands	Parents (mother and father) of children (male and female) 10–12 y.o.	Regional sample	356	To determine whether parents would accept HPV vaccination for their children and which variables may influence their decision, including knowledge about cervical cancer and HPV.	Quantitative non-randomized	75
Stretch S et al. [69]	2008	United Kingdom	Parents (mother and father) of female children 12-13 y.o.	Regional sample	651	To assess parental attitudes and information needs in an adolescent HPV vaccination programme	Quantitative non-randomized	75
Brabin L et al. [70]	2007	United Kingdom	Parents (mother and father) of children (male and female) 11–12 y.o.	Regional sample	317	To investigate parents’ views on making HPV vaccination available to adolescent minors at sexual health clinics without parental consent.	Mixed Method	100
Marlow L et al. [71]	2007	United Kingdom	Mothers of female children 11 (8–14) y.o.	Regional sample	684	To examine the association between general vaccine attitudes, trust in doctors and the government, past experience with vaccination and acceptance of HPV vaccination.	Quantitative non-randomized	50
Marlow L et al. [72]	2007	United Kingdom	Mothers of female children 11 (8–14) y.o.	Regional sample	684	To determine the acceptability of childhood HPV vaccination and examine demographic, cultural, and psychosocial predictors of vaccine acceptance.	Quantitative non-randomized	75
Woodhall S et al. [73]	2007	Finland	Adolescents (male and female) and their parents 14–15 y.o.	Regional sample	1124	To evaluate acceptance of HPV vaccination by adolescents and their parents.	Quantitative non-randomized	75
Brabin L et al. [12]	2006	United Kingdom	Parents (mother and father) of children (male and female) 11–12 y.o.	Regional sample	317	To assess parental consent and potential HPV vaccine uptake in eight secondary schools using stratified randomization according to school type and ethnicity	Quantitative non-randomized	50

List of studies are ordered by author, year, and country

### Results of studies evaluating HPV knowledge in adolescents and their parents

Thirty-eight studies reported data results on HPV knowledge. Table 2 presents the results of each study included in this systematic review grouped by headings and subheadings. A total of 154,090 adolescents and 75,597 parents answered one or some of the items included in this review. The percentage of adolescents that had heard about HPV varied greatly in the studies; from 5.2% [64] to 94.0% [54]. This same applied to parents: the percentage of parents that had heard about HPV varied between 29.5% [68] and 93.8% [74] depending on the study.

**Table 2:**
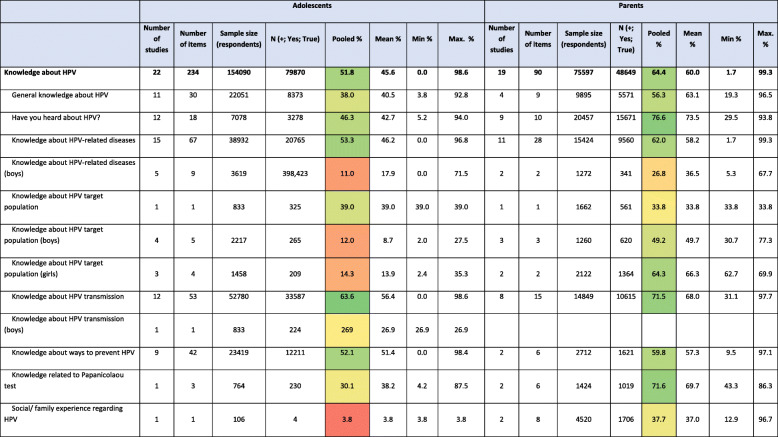

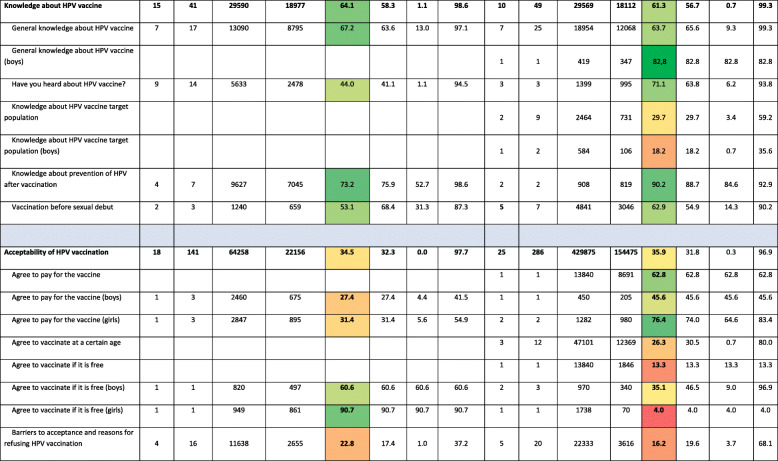

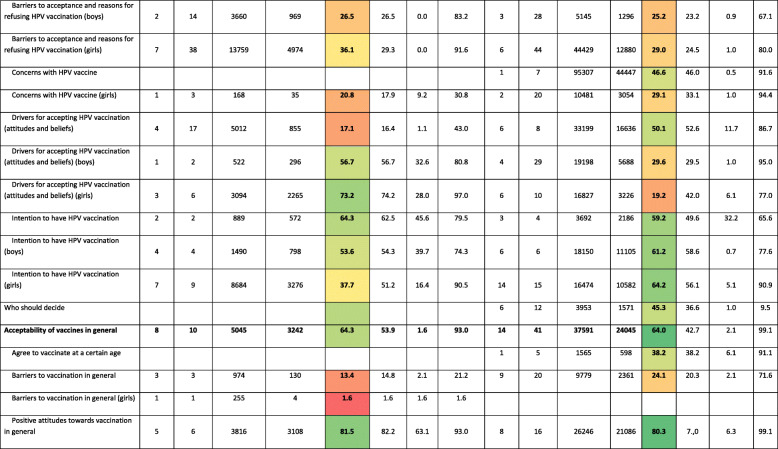
Knowledge about HPV and HPV vaccine and HPV vaccine acceptability in adolescents and their parents

Number of studies: number of studies reporting these data

Number of items: number of items reporting these data

Sample size (respondents): total number of respondents to any item under this heading or subheading across the included studies

N (+; Yes; True): total number of respondents who responded +, Yes or True to any item under this heading or subheading across the included studies

Pooled %: calculated value using previous columns N/sample size

Mean of %: average of the percentages reported in the studies for the items under this heading or subheading

Min %: minimum of the percentages reported in the studies for the items under this heading or subheading

Max. %: maximum of the percentages reported in the studies for the items under this heading or subheading

Colour scale:  from lowest to highest percentages

Between 1.1% [63] and 94.5% [47] of adolescents and 6.2% [68] and 93.8% [74] of parents said they had heard of HPV vaccine. Only one study assessed HPV vaccine knowledge in boys.

**Fig. 2:**
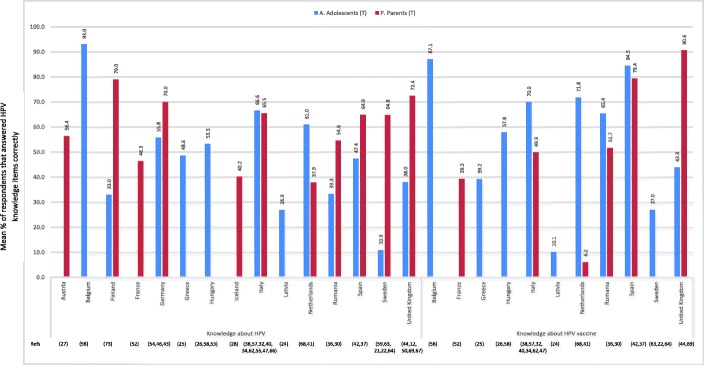
Percentage of HPV and vaccine knowledge per country

Studies performed in Finland were the ones showing the highest overall knowledge of HPV among parents; around 79% of Finnish respondents answered correctly to items related to HPV knowledge, followed by studies in the UK and Germany (72.4% and 74%, respectively). The lowest percentage was in The Netherlands (37.9%). Among adolescents, Belgium studies showed the highest percentage of HPV knowledge (93%), followed by Italian’s (66.6%), and the lowest was in Sweden (10.9%). HPV vaccination knowledge varied also between 6.2 and 90.6% in parents, being highest in the UK and lowest in the Netherlands studies; and between 10.1 and 87.1% in adolescents, being highest in Belgium and lowest in Latvia (Fig. 2).

Parents’ most common source of information on HPV was the pediatrician, 46.9% [66] to 92% [38] of the respondents depending on the publication, whereas for adolescents it was school ranging from 0% [36] to 61.3% [58]. However, the most common source of information of HPV vaccines was the vaccination centre for parents (77.3%) [38], although this figure came from a single study, and for adolescents it was family doctor or medical staff ranging from 4.2% [36] to 32% [32] (See Supplementary Material).

There were some differences regarding knowledge related to gender. Only 12.0% (pooled percentage) (ranging from 2.0% [63] to 27.5% [44]) of adolescents knew that males are target for HPV infection versus a pooled percentage of 13.9% that knew females are target for HPV infection (ranging from 2.4% [63] to 35.3% [22]).

### Results of studies evaluating HPV vaccine acceptance in adolescents and their parents

Forty studies reported data on HPV vaccine acceptance. Table 2 presents the results of each study included in this systematic review grouped by headings and subheadings. Further, 64,258 adolescents and 429,875 parents answered at least one of the items on acceptance included in the studies included in this systematic review.

The percentage of adolescents with positive intention to be vaccinated against HPV was 64.3% (pooled percentage), ranging from 45.6% [73] to 79.5% [26]. Between 39.7% [62] and 74.3% [63] of adolescents showed positive attitude toward boys HPV vaccination.

The percentage of parents with positive intention to vaccinate their children against HPV was 59.2% (pooled percentage) ranging from 32.2% [12] to 65.6% [62] and was quite similar in terms of vaccinating girls or boys.

**Fig. 3:**
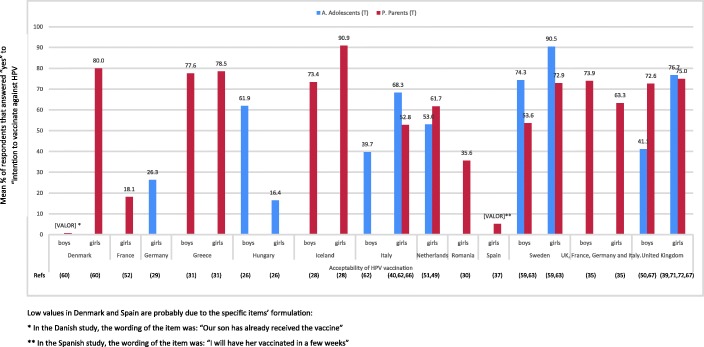
Intention to vaccinate girls/boys per country. Low values in Denmark and Spain are probably due to the specific items’ formulation: * In the Danish study, the wording of the item was: “Our son has already received the vaccine.” ** In the Spanish study, the wording of the item was: “I will have her vaccinated in a few weeks.”

By countries, studies conducted in Nordic countries like Sweden and Iceland presented the highest percentage of vaccine acceptance in adolescents and parents, respectively (Fig. 3).

Between 7.7% [21] and 32.5% [58] of adolescents, depending on the publication, had safety concerns about HPV vaccines, and 37% in one study [73] considered that HPV vaccination may encourage early sexual debut. Regarding HPV vaccination in males, between 68.7% [36] and 83.2% [36] of adolescents reported lack of information as the major reason for refusing vaccination.

Among parents, between 7.9% [71] and 68.1%% [30], depending on the publication, also reported safety concerns as the major barrier to refusing vaccination, followed by the idea that HPV vaccination may encourage sexual activity (10.5% [59] to 42% [73]). When questioned specifically about HPV vaccination in their sons, safety concerns (29.0% [60] to 67.1% [38]) and lack of information (25.5% [35] to 56.5% [35]) were also identified as the most common barriers.

In contrast, 7.7% [22] to 43.0% [63] of adolescents and 13.7% [59] to 73.4% [28] of parents considered the vaccine to be effective in protecting against HPV-related diseases. For HPV vaccination in males, 32.6% [63] to 80.8% [63] of adolescents and 17.9% [38] to 91.9% [48] of parents also perceived the vaccine to be effective in males. Moreover, 26.2% [35] to 84.6% [35] of parents considered HPV vaccination of their sons as a social responsibility.

### Factors associated with HPV knowledge in adolescents and their parents

The main sociodemographic factors associated with HPV knowledge in adolescents and their parents identified in this analysis were female gender (nine studies out of ten studying this factor), extent of higher education (five studies out of six), and higher income groups (one study out of two). Regardless of the investigators’ questions about HPV knowledge, the percentage of female adolescents knowing about HPV is consistently higher than that of boys: ranging from 16.4% [63] to 92.8% [55] in adolescent females across the publications versus 8.1% [24] to 51.3% [55] in adolescent males.

Adolescent’s age at first sexual intercourse, age of respondent parents, and religion were also identified in several publications as being related with HPV knowledge, although results are discrepant.

Additionally, being vaccinated against HPV or having a vaccinated older sister were also positively associated with levels of HPV knowledge.

A complete list of factors associated with HPV knowledge is included in the Supplementary Material.

### Factors associated with HPV vaccine acceptance

Up to 80 factors presented a statistically significant association with HPV vaccine acceptance in at least one of the studies included in this systematic review: 21 were sociodemographic or family characteristics, 37 factors were drivers, and 22 were barriers to vaccine acceptance.

Within demographic factors, female gender and younger age of respondent parent, female gender of the adolescent, higher household income, and previous childhood vaccinations are the ones most consistently associated with HPV vaccine acceptance.

Drivers associated with HPV vaccine acceptance in the studies were belief in vaccine efficacy (eight studies out of eight studying this factor), existing awareness of HPV (six studies out of six), belief that HPV vaccine prevents cervical cancer (six studies out of six), susceptibility to HPV infection (four studies out of five), receiving information from the doctor (four studies out of four), desire to fit in social norms (four studies out of four), perception of disease severity (three studies out of three), and intention to do Pap test (three studies out of three).

The most frequently identified barriers to HPV vaccine acceptance were doubts about HPV vaccine safety profile (12 studies out of 12) followed by the belief that the vaccine will impact sexual behavior (six studies out of six), low perceived susceptibility to HPV infection (three studies out of three), and doubts about HPV vaccine efficacy (three studies out of three).

A complete summary of the factors associated with parental and adolescent HPV knowledge and vaccine acceptance is included in the Supplementary Material.

### Measurement tools used to evaluate HPV knowledge and vaccine acceptance in adolescents and their parents

A complete list of items used in these questionnaires, including 38 and 40 questionnaires to assess HPV knowledge and vaccine acceptance respectively, is provided in the Supplementary Material.

## Discussion

This review shows that HPV knowledge and vaccine acceptance vary widely across different studies and countries. In general, figures are still modest and lower in comparison with other routine vaccines. Safety concerns are still the main barrier to vaccination, and lack of HPV and vaccine knowledge has been identified, which is even greater for male vaccination. In contrast, main drivers to vaccination are perception of efficacy of HPV vaccine and social responsibility.

To our knowledge, this is the first systematic literature review to summarize factors influencing HPV knowledge and vaccine acceptance among adolescents and their parents since the marketing authorization of HPV vaccines in Europe and complementary to a recent systematic review on HPV vaccine hesitancy in Europe [75]. Additionally, it provides a compilation of the measurement tools, items, and questionnaires used in these published studies that can be useful for future research.

Vaccination acceptance is critical to ensuring the success of national immunization programs. Previous knowledge has been already identified as a known prerequisite for informed decision-making and vaccine acceptance [16]. HPV vaccination coverage rates and parental acceptance have been a subject of debate over the last decade, as they are still lower than expected in comparison with other vaccines administered routinely to adolescents. By May 2018, HPV immunization programs had been introduced in 80 countries, areas, or territories [76]. Although Scientific Societies and Public Health Authorities have made great efforts to guarantee the success of vaccination programs, there is still room for improvement, as vaccination coverage rate is still under 50% in many European countries [77]. For instance, Ireland and Denmark recently registered a decline in HPV vaccine uptake due to parental concerns. Cross-sectorial alliances between educational, parental, scientific, and political bodies were necessary to overcome this issue in those countries and ensure protection against morbidity and mortality associated to HPV related-cancer [78].

Recognition of the social and economic impact of the entire HPV burden of disease is still inadequate [1]: recurrent respiratory papillomatosis in children, highly contagious infections in adolescents, genital warts, precancerous lesions in young adults that may have consequences for reproductive capacity, and finally, a considerable number of cancers in different anatomic locations that affect males and females every year. The vast majority of this burden is attributable to HPV genotypes whose infection can be prevented with HPV vaccination. However, the population is not very aware of all this information [1] which is also reflected in our systematic review.

Our results show that HPV knowledge are still moderate and vary widely between European countries and the populations interviewed. These results are substantially aligned with findings from previous publications: according to Loke et al. [16], the percentage of adolescents that had heard about HPV infection and HPV vaccines varied between 21.5 and 77.6% and 9.9 and 40.3%, with Malaysia and Hong Kong being the countries with the highest percentage and Latvia the lowest. In parents, between 49.0% (USA) and 92.7% (Canada) had heard of HPV and between 43.7% (Hong Kong) and 95% (USA) about HPV vaccine. According to Radisic et al. [17], knowledge of HPV infection and vaccine in the male population was mostly modest, and parents often expressed a need for more information about HPV vaccine before taking decision about their sons’ vaccination.

In our study, percentages of HPV vaccination acceptance are also quite modest and lower than for vaccines in general. The percentages of parents and adolescents that intended to vaccinate or receive the vaccination were 59.2% and 64.3%, respectively.

As previously described, our results showed that female gender and having higher education impact HPV knowledge and vaccine acceptance positively. Main drivers for HPV vaccination included the perception of HPV severity and impact and the belief that HPV vaccines are effective. This was also identified in previous publications [20] and further work is required to increase HPV awareness in the population. The source of information is also known to be critical for a positive attitude—when information is provided by the doctor, a greater level of HPV knowledge and a vaccine acceptance were shown in parents and adolescents. A reliable source that provides balanced and understandable scientific information seems to be critical to making well-informed decisions. On the contrary, most common barriers were the idea that HPV vaccination may encourage sexual activity, and safety concerns.

More than 11 years after authorization, HPV vaccines have proven to be effective [79–83] and to have a favorable safety profile, as shown in clinical trials and post-authorization studies, in which a rapid reduction in HPV-related diseases has been observed following vaccine introduction [84, 85]. This has been greatly acknowledged by major Health Bodies, such as the WHO [85], which in 2017 stated that accumulated safety studies including several million people showed no new adverse events of concern. More recently, the WHO has urged countries to set cervical cancer elimination goal and in other countries, such as the USA, Scientific Societies have even promoted HPV-cancer elimination goal, since HPV is responsible for a variety of cancers other than cervical cancer that affect both genders [86].

So far, 28 countries have extended their HPV national immunization programs to include boys and prevent them from suffering HPV-related diseases [76]. However, there is even less awareness of HPV infection in males and the fact that they can also benefit from HPV vaccination. According to our results, a small percentage of adolescents knew that males are targets of HPV infection, and only 5.3% of mothers were aware about male diseases caused by HPV infection. Lack of information has also been identified in this and previous reviews as a key barrier to HPV vaccination in males [16]. Therefore, concerted efforts should be made to increase awareness of HPV infection in males and to ensure the success of HPV gender-neutral vaccination programs.

A recent review [87] has shown that informational strategies may influence the intent to vaccinate by increasing HPV-related knowledge and awareness, but the effect on HPV vaccine behavior is minimal. The most effective strategy to change vaccination behavior is multifaceted. This is consistent with a previous review [88] that also stressed the effectiveness of interventions that target both the provider and the patient.

Also, we found that HPV knowledge and vaccine acceptance varied by several patients’ characteristics (in particular their socioeconomic background, as already found for other vaccines [89]). These results highlight the need for “tailored” interventions, carefully designed to respond to specific concerns and beliefs of the target population in order to reduce social inequalities in vaccination.

The items compiled in the different questionnaires could be useful for countries and investigators who intend to assess HPV knowledge and vaccine acceptance in their countries to make their results comparable with existing published data and to assess temporal trends or factors that influence the variations in HPV knowledge and vaccine acceptance.

Main limitations of this review stem from the variability across the studies included and, particularly from the lack of coincidence of the items used in each study to measure HPV knowledge and vaccine acceptance in adolescents and their parents. Also, it is difficult to assess a temporal trend in HPV knowledge and vaccine acceptance, as the questionnaires used and target populations differed widely between studies. Other limitations are heterogeneous sampling methods, sample sizes, population included, year of the study, and HPV vaccine implementation in the country. Also, this study is focused only in studies conducted in Europe and results cannot be extrapolated worldwide. This systematic review excluded all non-English-language publications. However, since most relevant research is commonly published in international journals in English, we do not expect to have missed any relevant study. Nevertheless, pooled results should be interpreted with caution.

## Conclusions

Concerted efforts should be made to conduct multifaceted and tailored interventions to the population providing balanced information for decision-making on HPV vaccination. Increasing HPV vaccination uptake in males and females could dramatically change the epidemiology of HPV-related diseases and their consequences in countries.

## Supplementary information

**Additional file 1.** SM-1- Search strategy.

## Data Availability

Not applicable.

## Abbreviations

CIN: Cervical intraepithelial lesions
ECDC: European Centre for Disease Prevention and Control
EMA: European Medicines Agency
EU: European Union
HPV: Human Papillomavirus
MMAT: Mixed Method Assessment Tool
STI: Sexually transmitted infections
WHO: World Health Organization
