# Correction to: HPV knowledge and vaccine acceptance among European adolescents and their parents: a systematic literature review

**DOI:** 10.1186/s40985-020-00130-9

**Published:** 2020-07-21

**Authors:** Noelia López, Maria Garcés-Sánchez, Maria Belén Panizo, Ignacio Salamanca de la Cueva, Maria Teresa Artés, Beatriz Ramos, Manuel Cotarelo

**Affiliations:** 1 Medical Affairs Department, Merck Sharp & Dohme Spain, Madrid, Spain; 2 Nazaret Healthcare Center, Valencia, Spain; 3 Illescas Healthcare Center, Toledo, Spain; 4 Instituto Hispalense de Pediatría, Sevilla, Spain; 5 Adelphi Spain, Barcelona, Spain

**Correction to: Public Health Rev 41, 10 (2020)**

**https://doi.org/10.1186/s40985-020-00126-5**

In the original publication of this article [1] a large part of Table 2 was missing due to an error during typesetting.

**Table 2:**
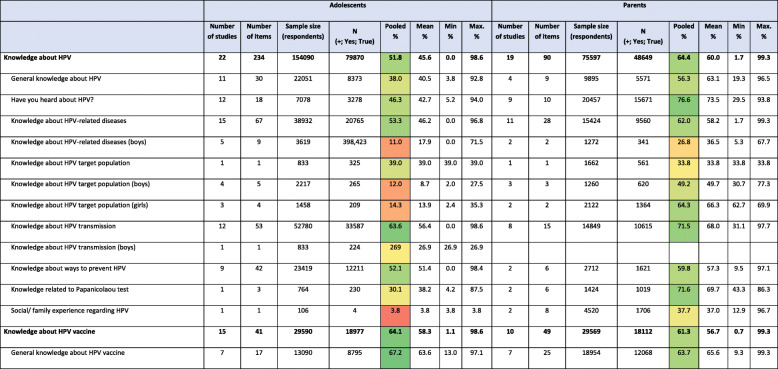
Knowledge about HPV and HPV vaccine and HPV vaccine acceptability in adolescents and their parents

The full table is published in this correction article. The original publication has been updated.

The publisher apologizes to the authors & readers for the inconvenience.
