# Supplementary material for: HPV knowledge and vaccine acceptance among European adolescents and their parents: a systematic literature review
Source: Public Health Rev. 2020 May 14;41:10. doi: 10.1186/s40985-020-00126-5 (PMC7222509; doi:10.1186/s40985-020-00126-5)
Supplement: Supplementary file 1 — Additional file 1. SM-1- Search strategy. [file 40985_2020_126_MOESM1_ESM.docx]

**Supplementary material**

**SM-1- Search strategy**

| DATABASE | QUERIES | ADDITIONAL FILTERS | Nº of RESULTS |
| --- | --- | --- | --- |
| PubMed  ([www.ncbi.nlm.nih.gov](http://www.ncbi.nlm.nih.gov)  /pubmed) | - (human papillomavirus OR HPV) AND (immunis* O immuniz* OR vaccin*) AND (knowledg* OR accept* OR attitud* OR percept* OR decision* OR choice* OR motivat* OR uptak* OR barrier* OR refus* OR participat* OR attitud* OR intent*) AND (austria OR belgium OR Bulgaria OR Croatia OR Cyprus OR Czech Republic OR Denmark OR Estonia OR Finland OR France OR Germany OR Greece OR Hungary OR Iceland OR Ireland OR Italy OR Latvia OR Liechtenstein OR Lithuania OR Luxemburg OR Macedonia OR Netherlands OR Norway OR Portugal OR Serbia OR Slovenia OR Spain OR Sweden OR Switzerland OR United Kingdom OR Malta OR Poland OR Romania OR Slovakia)] NOT review | **Publication dates**: 01/01/2006 – 31/12/2017  **Text availability**: Abstract  **Species**: Humans  **Language**: English | 609 citations |
| Cochrane  (<http://www.cochrane>  library.com) | - (human papillomavirus OR HPV) AND (immunis* OR immuniz* OR vaccin*) AND (knowledg* OR accept* OR attitud* OR percept* OR decision* OR choice* OR motivat* OR uptak* OR barrier* OR refus* OR participat* OR attitud* OR intent*) [All text] - human papillomavirus AND (survey OR questionnaire OR assessment) AND (knowledge OR acceptability OR attitudes) AND vaccine [All text] | **Publication dates**: 2006 – 2018  **Cochrane database**: Trials, Method studies, technology assessment, Cochrane groups | 206 citations |
| EMBASE  (www.embase.com) | - ('human papillomavirus':ab,ti OR 'hpv':ab,ti) AND ('survey':ab,ti OR 'questionnaire':ab,ti OR 'assessment':ab,ti) AND ('knowledge':ab,ti OR 'acceptability':ab,ti OR 'attitudes':ab,ti) AND 'vaccine':ab,ti AND [2006-2018]/py AND [english]/lim - ('human papillomavirus':ab,ti OR 'hpv':ab,ti) AND ('immunis*':ab,ti OR 'immuniz*':ab,ti OR 'vaccin*':ab,ti) AND ('knowledg*':ab,ti OR 'accept*':ab,ti OR 'percept*':ab,ti OR 'decision*':ab,ti OR 'choice*':ab,ti OR 'motivat*':ab,ti OR 'uptak*':ab,ti OR 'barrier*':ab,ti OR 'refus*':ab,ti OR 'participat*':ab,ti OR 'attitud*':ab,ti OR 'intent':ab,ti) AND ('austria':ab,ti OR 'belgium':ab,ti OR 'bulgaria':ab,ti OR 'croatia':ab,ti OR 'cyprus':ab,ti OR 'czech republic':ab,ti OR 'denmark':ab,ti OR 'estonia':ab,ti OR 'finland':ab,ti OR 'france':ab,ti OR 'germany':ab,ti OR 'greece':ab,ti OR 'hungary':ab,ti OR 'iceland':ab,ti OR 'ireland':ab,ti OR 'italy':ab,ti OR 'latvia':ab,ti OR 'liechtenstein':ab,ti OR 'lithuania':ab,ti OR 'luxemburg':ab,ti OR 'macedonia':ab,ti OR 'netherlands':ab,ti OR 'norway':ab,ti OR 'portugal':ab,ti OR 'serbia':ab,ti OR 'slovenia':ab,ti OR 'spain':ab,ti OR 'sweden':ab,ti OR 'switzerland':ab,ti OR 'united kingdom':ab,ti OR 'malta':ab,ti OR 'poland':ab,ti OR 'romania':ab,ti OR 'slovakia)':ab,ti) AND [2006-2018]/py NOT 'review':ab,ti AND [english]/lim | **Publication dates**: 2006 – 2018  **Language**: English | 1124 citations |
| World Bank Group  (www.worldbank.org /en/research/brief/  publications) | - human papillomavirus AND (survey OR questionnaire OR assessment) AND (knowledge OR acceptability OR attitudes) AND vaccine NOT review | **Publication dates**: 2006 – 2018  **Language**: English | 141 citations |
| Popline  (www.popline.org) | - (( ( ( human papillomavirus ) ) ) OR ( ( ( hpv ) ) ) AND ( ( ( survey ) OR ( questionnaire ) OR ( assessment ) ) ) AND ( ( ( vaccine ) ) )) AND ( ( Language: English ) AND ( Publication Year:[2006 TO 2018] ) ) - (( ( ( human papillomavirus ) ) ) OR ( ( ( hpv ) ) ) AND ( ( ( knowledge ) OR ( acceptability ) OR ( attitudes ) ) ) AND ( ( ( vaccine ) ) )) AND ( ( Language:English ) AND ( Publication Year:[2006 TO 2018] ) ) - (( ( ( human papillomavirus ) ) ) OR ( ( ( hpv ) ) ) AND ( ( ( knowledge ) OR ( acceptability ) OR ( attitudes ) ) )) AND ( ( Language:English ) AND ( Publication Year:[2006 TO 2018] ) ) - (( ( ( human papillomavirus ) ) ) OR ( ( ( hpv ) ) ) AND ( ( ( survey ) OR ( questionnaire ) OR ( assessment ) ) )) AND ( ( Language:English ) AND ( Publication Year:[2006 TO 2018] ) ) | **Publication dates**: 2006 – 2018  **Language**: English  **Region/Country**: Europe | 38 citations |

**SM-2- Tool for quality assessment**

Method Assessment Tool (MMAT) developed by Pluye et al(1)


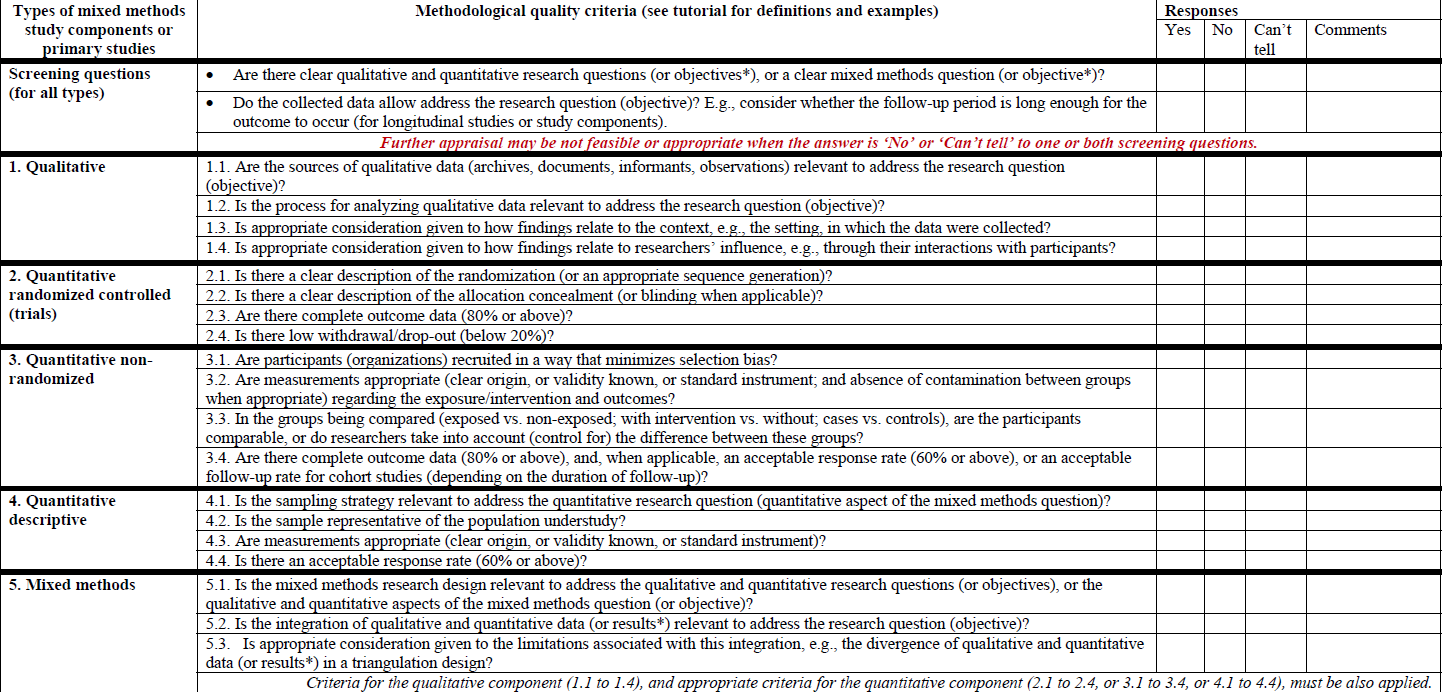


**SM-3- Data extraction form**

The following data extraction sheet was used to extract the data from all the studies included in the qualitative and quantitative synthesis

| Full reference |  |
| --- | --- |
| Internal Id |  |
| Title |  |
| Study aim |  |
| Year of publication | \|_\|_\|_\|_\| |
| Type of publication | \|_\| Original Paper |
| Study design | \|_\| Quantitative non-randomized  \|_\| Quantitative randomized controlled  \|_\| Qualitative  \|_\| Mixed Method |
| Study methods | *Multiple answer*  \|_\| Interviews  \|_\| Inductive content analysis  \|_\| Focus groups  \|_\| Paper & pencil-administered questionnaire  \|_\| Phone-administered questionnaire  \|_\| On line-administered questionnaire  \|_\| Computer-assisted personal interviews (CAPI) |
| Theoretical model | *Multiple answer*  \|_\| Theory of planned behaviour and protection motivation theory  \|_\| Health Belief Model  \|_\| Precaution Adoption Process Model  \|_\| Other, specify: _____________  \|_\| No theoretical model |
| Study setting | \|_\| Regional sample  \|_\| National sample  \|_\| Multinational sample |
| HPV vaccination program description | To be detailed up to the maximum level of available information  **Vaccination target:**  \|_\| Girls  \|_\| Boys and girls  **Vaccine coverage rate:**  \|_\| 0%  \|_\| less than 50%  \|_\| 50%-80%  \|_\| more than 80% |
| Sample size description | To be detailed up to maximum level of available information  \|_\|_\|_\| male children from \|_\|_\| to \|_\|_\| years old  \|_\|_\|_\| female children from \|_\|_\| to \|_\|_\| years old  \|_\|_\|_\| parents of male children from \|_\|_\| to \|_\|_\| years old  \|_\|_\|_\| parents of female children from \|_\|_\| to \|_\|_\| years old  \|_\|_\|_\| parents of children from \|_\|_\| to \|_\|_\| years old  \|_\|_\|_\| mothers of male children from \|_\|_\| to \|_\|_\| years old  \|_\|_\|_\| mothers of female children from \|_\|_\| to \|_\|_\| years old  \|_\|_\|_\| fathers of male children from \|_\|_\| to \|_\|_\| years old  \|_\|_\|_\| fathers of female children from \|_\|_\| to \|_\|_\| years old |
| Measurement tool | \|_\| y/n |
| Knowledge assessment method/scale | \|_\| y/n  \|_\| true/false  \|_\| ordinal scale  \|_\| other, specify: ________ |
| Knowledge assessment items | List of items |
| Subheading of knowledge | \|_\| HPV knowledge  \|_\| HPV vaccine knowledge |
| Subheading 2 | \|_\| Cervical cancer knowledge  \|_\| Diagnostic  \|_\| HPV knowledge  \|_\| HPV sources of information  \|_\| HPV vaccination beliefs  \|_\| HPV vaccine sources of information  \|_\| Papanicolaou test knowledge  \|_\| Parent and child confidence  \|_\| Post-vaccination behaviour  \|_\| Prevention of STDs  \|_\| Prevention of HPV  \|_\| Risk factors  \|_\| Sexual behaviour  \|_\| STI knowledge  \|_\| Transmission knowledge  \|_\| Unvaccinated/vaccination status |
| Knowledge assessment results | % of y/n (or the relevant scale) per item and factor provided |
| n | \|_\| |
| Acceptance assessment method/scale | \|_\| y/n  \|_\| true/false  \|_\| ordinal scale  \|_\| other, specify: ________ |
| Acceptability assessment items | List of items |
| Subheading of acceptability | \|_\| HPV vaccination acceptability  \|_\| Knowledge of vaccines and its acceptability |
| Subheading 2 | \|_\| Attitudes towards HPV vaccination  \|_\| Attitudes towards vaccination  \|_\| Barriers  \|_\| HPV barriers  \|_\| HPV severity  \|_\| HPV vaccine efficacy  \|_\| Intention to have HPV vaccination  \|_\| Pap-test attitude  \|_\| Reasons for accepting HPV  \|_\| Reasons for refusing HPV vaccination  \|_\| Susceptibility  \|_\| Vaccination Barriers  \|_\| Visit reason |
| Acceptance assessment results | % of y/n (or the relevant scale) per item and factor provided |
| n | \|_\| |
| MMAT score | \|_\|_\|_\| % |
| Factors | \|_\| y/n |
| Factors evaluated for association with knowledge | List of factors and statistical parameter and significance (e.g. OR, p-value) |
| Factors evaluated for association with acceptance | List of factors and statistical parameter and significance (e.g. OR, p-value) |

**SM-4- List of all studies excluded and reasons for exclusion**

| Title | Exclusion criterion |
| --- | --- |
| Ward JK, Crépin L, Bauquier C, Vergelys C, Bocquier A, Verger P, Peretti-Watel P. ‘I don’t know if I’m making the right decision’: French mothers and HPV vaccination in a context of controversy, Health, Risk & Society, 2017;19 (1-2), 38-57 | Qualitative study |
| Gottvall M, Stenhammar C, Grandahl M. Parents' views of including young boys in the Swedish national school-based HPV vaccination programme: A qualitative study. BMJ Open 2017;7:e014255. | Qualitative study |
| Pot M, Paulussen TG, Ruiter RA, Eekhout I, de Melker HE, Spoelstra ME, et al. Effectiveness of a Web-Based Tailored Intervention With Virtual Assistants Promoting the Acceptability of HPV Vaccination Among Mothers of Invited Girls: Randomized Controlled Trial. J Med Internet Res. 2017 Sep 6;19(9):e312. | Aim of the study |
| Romejko-Wolniewicz E. Cervical cancer - Knowledge, prevention and exposure to risk factors among students from various countries. IFCPC2017 World Congress. Orlando, Fl, USA2017. | Conference abstract |
| HPV: Warts and all! an evaluation of knowledge and attitudes to HPV disease and the HPV vaccination, pre and post a 15-minute animated video. Irish Journal of Medical Science 185:546-546, December 2016. | Aim of the study |
| Mupandawana ET, Cross R. Attitudes towards human papillomavirus vaccination among African parents in a city in the north of England: A qualitative study. Reproductive Health 2016; 13:97 | Qualitative study |
| Zeraiq L, Nielsen D, Sodemann, M. Attitudes towards human papillomavirus vaccination among Arab ethnic minority in Denmark: A qualitative study. Scandinavian Journal of Public Health, 2015; 43: 408–414 | Qualitative study |
| Batista-Ferrer H, Trotter CL, Hickman M, Audrey, S. Barriers and facilitators to uptake of the school-based HPV vaccination programme in an ethnically diverse group of young women. Journal of Public Health 2015;38(3):569–577 | Qualitative study |
| Grandahl M, Oscarsson M, Stenhammar C, Nevéus T, Westerling R, Tydén T. Not the right time: Why parents refuse to let their daughters have the human papillomavirus vaccination. Acta Paediatrica, International Journal of Paediatrics 2014;103(4):436-441 | Qualitative study |
| Hofman R, De Bekker-Grob EW, Raat H, Helmerhorst TJ, Van Ballegooijen M, Korfage IJ. Parents’ preferences for vaccinating daughters against human papillomavirus in the Netherlands: A discrete choice experiment. BMC Public Health. 2014;14(1):1–8. | Aim of the study |
| Hofman R, De Bekker-Grob EW, Richardus JH, De Koning HJ, Van Ballegooijen M, Korfage IJ. Have preferences of girls changed almost 3 years after the much debated start of the HPV vaccination program in the Netherlands? A discrete choice experiment. PLoS One. 2014;9(8). | Aim of the study |
| Wegwarth O, Kurzenhäuser-Carstens S, Gigerenzer G. Overcoming the knowledge-behavior gap: The effect of evidence-based HPV vaccination leaflets on understanding, intention, and actual vaccination decision. Vaccine. 2014;32(12):1388–93. | Aim of the study |
| Gottvall M, Grandahl M, Höglund AT, Larsson M, Stenhammar C, Andrae B, Tydén T. rust versus concerns-how parents reason when they accept HPV vaccination for their young daughter. Upsala Journal of Medical Sciences. 2013; 118: 263–270 | Qualitative study |
| Hilton S, Patterson C, Smith E, Bedford H, Hunt K. Teenagers' understandings of and attitudes towards vaccines and vaccine-preventable diseases: A qualitative study. Vaccine 2013;31:2543– 2550 Contents | Qualitative study |
| Hofman R, Van Empelen P, Vogel I, Raat H, Van Ballegooijen M, Korfage IJ. Parental decisional strategies regarding hpv vaccination before media debates: A focus group study. J Health Commun. 2013;18(7):866–80. | Aim of the study |
| Lutringer-Magnin D, Kalecinski J, Cropet C, Barone G, Ronin V, Régnier V, et al. Prevention of sexually transmitted infections among girls and young women in relation to their HPV vaccination status. Eur J Public Health. 2013;23(6):1046–53. | Aim of the study |
| Steckelberg A, Albrecht M, Kezle A, Kasper J, Mühlhauser I. Impact of numerical information on risk knowledge regarding human papillomavirus (HPV) vaccination among schoolgirls: a randomised controlled trial. Ger Med Sci. 2013;11:1–9. | Aim of the study |
| Knowledge of HPV among low-income minority mothers of adolescents 9-17 years of age. Women’s Health 2013: the 21^st^ Annual Congress, Washington, DC, 2013. | Conference abstract |
| Mothers, fathers, and human papillomavirus vaccine: How do they mix? Southern Regional Meeting Abstracts. Journal of Investigative Medicine & Volume 60, Number 1, January 2012. | Conference abstract |
| Craciun C, Baban A, " Who will take the blame?": Understanding the reasons why Romanian mothers decline HPV vaccination for their daughters. Vaccine 2012;30: 6789– 6793 | Qualitative study |
| Abstracts presented at the 17th International Meeting of the European Society of Gynaecological Oncology. What parents of young girls think about HPV vaccination. International Journal of Gynecological Cancer: October 2011 - Volume 21 - Issue 12 - ppg S1-S1372 | Conference abstract |
| Gordon D, Waller J, Marlow LAV. Attitudes to HPV vaccination among mothers in the British Jewish community: Reasons for accepting or declining the vaccine. Vaccine 2011;29: 7350– 7356 | Qualitative study |
| Henderson L, Clements A, Damery S, Wilkinson C, Austoker J, Wilson S. 'A false sense of security' understanding the role of the HPV vaccine on future cervical screening behaviour: A qualitative study of UK parents and girls of vaccination age. J Med Screen 2011;18:41–45 | Qualitative study |
| Hilton S, Smith E. " I thought cancer was one of those random things. I didn't know cancer could be caught...": Adolescent girls' understandings and experiences of the HPV. Vaccine 2011; 29: 4409–4415 | Qualitative study |
| Williams K, Forster A, Marlow L, Waller J. Attitudes towards human papillomavirus vaccination: A qualitative study of vaccinated and unvaccinated girls aged 17-18 years. J Fam Plann Reprod Health Care 2011;37:22–25. | Qualitative study |
| Fahy A, Desmond DM. Irish mothers’ intentions to have daughters receive the HPV vaccine. Ir J Med Sci. 2010;179(3):427–30. | Aim of the study |
| HPV awareness and knowledge among Swiss students: A nationwide web-based survey. Arch Gynecol Obstet (2010) 282 (Suppl 1):S63–S270 | Conference abstract |
| Marlow L, Wardle J, Waller J. Attitudes to HPV vaccination among ethnic minority mothers in the UK: An exploratory qualitative study. Human Vaccines 2009; 5(2):105-110 | Qualitative study |
| Marlow LAV, Forster AS, Wardle J, Waller J. Mothers’ and Adolescents’ Beliefs about Risk Compensation following HPV Vaccination. J Adolesc Heal. 2009;44(5):446–51. | Aim of the study |
| Williams K et al. Family history of cervical cancer-influence on patients' knowledge and preventive behaviour. Cancer January 1, 2009. | Conference abstract |
| Stretch R. Implementing a school-based HPV vaccination programme. Nurs Times. 2008 Dec 2-8;104(48):30-3. | Aim of the study |
| Human papillomavirus (HPV) and HPV vaccines: The knowledge levels, opinions, and behaviours of parents. APhA2013, March 1–4, 2013, Los Angeles, CA. | Conference abstract |
| Paul Gard ET AL. Parents / Carers Views and Decisions about the Human Papillomavirus Vaccine. Yearbook of Pediatric Endocrinology 2010. | Conference abstract |
| Attitudes toward HPV vaccine: Differences among parents of male and female adolescents. ESC Congress Library. Aksu H, May28, 2014, 50646 | Conference abstract |
| Knowledge and opinion of adolescent girls and their mothers regarding HPV vaccine. ESC Congress Library, Aksu H, May 28, 2014, 50646. | Conference abstract |
| Höglund AT et al. Knowledge of human papillomavirus and attitudes  to vaccination among Swedish high school students. International Journal of STD & AIDS 2009; 20: 102–107. | Duplicated |
| Balemans R, Devroey D, Van De Vijver E, Chovanova H, Vandevoorde J. Knowledge and attitudes about vaccinations among adolescents. J Prev Med Hyg. 2011;52(2):64–72. | Duplicated |
| Paul Gard ET AL. Parents / Carers Views and Decisions about the Human Papillomavirus Vaccine. Yearbook of Pediatric Endocrinology 2010. | Duplicated |
| Kose D, Erkorkmaz U, Cmar N, Altinkaynak S. Mothers’ Knowledge and Attitudes aboout HPV Vaccination to prevent Cervical Cancers. Asian Pac J Cancer Prev. 2014;15(17):7263–6. | European (Vaccine non-commercialized) |
| Napolitano F, Napolitano P, Liguori G, Angelillo IF. Human papillomavirus infection and vaccination: Knowledge and attitudes among young males in Italy. Hum Vaccin Immunother. 2016,12(6):1504–10. | No segregation data |
| Di Giuseppe G, Abbate R, Liguori G, Albano L, Angelillo IF. Human papillomavirus and vaccination: Knowledge, attitudes, and behavioural intention in adolescents and young women in Italy. Br J Cancer. 2008;99(2):225–9. | No segregation data |
| Mehu-Parant F, Rouzier R, Soulat JM, Parant O. Eligibility and willingness of first-year students entering university to participate in a HPV vaccination catch-up program. Eur J Obstet Gynecol Reprod Biol. 2010;148(2):186–90. | No segregation data |
| Gottvall M, Tydén T, Höglund AT, Larsson M. Knowledge of human papillomavirus among high school students can be increased by an educational intervention. Int J STD AIDS. 2010;21(8):558–62. | No segregation data |
| Medeiros R, Ramada D. Knowledge differences between male and female university students about human papillomavirus (HPV) and cervical cancer: Implications for health strategies and vaccination. Vaccine. 2010;29(2):153–60. | No segregation data |
| Richard de Visscr, Louisa Waite, Chandni Parikhll and Aaron Lawrieri. The importance of social norms for uptake of catch-up human papillomavirus vaccination in young women. Sexual Health 331, 1448-5028/ I I /030330 | No segregation data |
| C.E. Schmeink, K.C.M. Gosens, W.J.G. Melchers, L.F.A.G. Massuger, R.L.M. Bekkers. Young adult’s awareness of HPV and vaccine acceptance after introduction of the HPV vaccine in the Dutch national vaccination program. Eur. J. Gynaec. Oncol. - ISSN: 0392-2936 XXXII, n. 5, 2011 | No segregation data |
| Donadiki EM, Jimenez-Garcia R, Hernandez-Barrera V, Carrasco-Garrido P, De Andres AL, Jimenez-Trujillo I, et al. Knowledge of the HPV vaccine and its association with vaccine uptake among female higher-education students in Greece. Hum Vaccines Immunother. 2013;9(2):300–5. | No segregation data |
| Kamzol W, Jaglarz K, Tomaszewski KA, Puskulluoglu M, Krzemieniecki K. Assessment of knowledge about cervical cancer and its prevention among female students aged 17-26 years. Eur J Obstet Gynecol Reprod Biol [Internet]. 2013;166(2):196–203. | No segregation data |
| McCusker SM, MacQueen I, Lough G, MacDonald AI, Campbell C, Graham S V. Gaps in detailed knowledge of human papillomavirus (HPV) and the HPV vaccine among medical students in Scotland. BMC Public Health [Internet]. 2013;13(1):1. | No segregation data |
| Michail G, Smaili M, Vozikis A, Jelastopulu E, Adonakis G, Poulas K. Female students receiving post-secondary education in Greece: The results of a collaborative human papillomavirus knowledge survey. Public Health. 2014;128(12):1099–105. | No segregation data |
| Schwarz TM, Honsberg T, Stephan N, Dannecker C, Gallwas J, Crispin A, et al. HPV vaccination: acceptance and influencing factors among young men in Germany. Future Microbiol. 2016;11(2):227–34. | No segregation data |
| Bettina Claudia Balla, András Terebessy, Emese Tóth and Péter Balázs. Young Hungarian student’s knowledge about HPV and their attitude toward HPV vaccination. Vaccines 2017, 5, 1; doi:10.3390/vaccines5010001 | No segregation data |
| Septimiu Voidăzan, Silviu-Horia Morariu, Monica Tarcea,  Horatiu Moldovan, Ioana Curticăpian, Minodora Dobreanu6 Human Papillomavirus (HPV) Infection and HPV Vaccination: Assessing the Level of Knowledge among Students of the University of Medicine and Pharmacy of Targus Murres, Romania. Acta Dermatovenerol Croat 2016;24(3):193-202. | No target population |
| Marlow LA V, Wardle J, Grant N, Waller J. Human papillomavirus (HPV) information needs: a theoretical framework. J Fam Plann Reprod Health Care. 2009;35(1):29–33. | No target population |
| Blödt S, Holmberg C, Müller-Nordhorn J, Rieckmann N. Human Papillomavirus awareness, knowledge and vaccine acceptance: A survey among 18-25 year old male and female vocational school students in Berlin, Germany. Eur J Public Health. 2012;22(6):808–13. | No target population |
| Sotiriadis A, Dagklis T, Siamanta V, Chatzigeorgiou K, Agorastos T. Increasing fear of adverse effects drops intention to vaccinate after the introduction of prophylactic HPV vaccine. Arch Gynecol Obstet. 2012 Jun 14 [cited 2018 Mar 1];285(6):1719–24. | No target population |
| Haesebaert J, Lutringer-Magnin D, Kalecinski J, Barone G, Jacquard AC, Leocmach Y, et al. Disparities of perceptions and practices related to cervical cancer prevention and the acceptability of HPV vaccination according to educational level in a French cross-sectional survey of 18-65 years old women. PLoS One. 2014;9(10):1–8. | No target population |
| Dempsey A et al. Understanding the Reasons Why Mothers Do or Do Not Have Their Adolescent Daughters Vaccinated Against Human Papillomavirus. Ann Epidemiol. 2009 August; 19(8): 531–538. | Non-European |
| Chan SSC, Yan Ng BH, Lo WK, Cheung TH, Hung Chung TK. Adolescent Girls’ Attitudes on Human Papillomavirus Vaccination. J Pediatr Adolesc Gynecol [Internet]. 2009;22(2):85–90. | Non-European |
| Waller J, Ostini R, Marlow LAV, McCaffery K, Zimet G. Validation of a measure of knowledge about human papillomavirus (HPV) using item response theory and classical test theory. Prev Med (Baltim). 2013;56(1):35–40. | Non-European |
| Teenager understanding of human papillomavirus (HPV) and acceptance of HPV vaccination. Abstracts Presented for the Fortieth Annual Meeting of the Society of Gynecologic Oncologists. February 2009Volume 112, Issue 2, Supplement, Pages S2–S185. | Non-European |
| Irvine UC. O934 HPV knowledge in low income and minority parents. Int J Gynecol Obstet. 2009;107:S359 | Non-European |
| Wong LP, Sam IC. Ethnically diverse female university students’ knowledge and attitudes toward human papillomavirus (HPV), HPV vaccination and cervical cancer. Eur J Obstet Gynecol Reprod Biol. 2010;148(1):90–5. | Non-European |
| Ferris D, Horn L, Waller JL. Parental Acceptance of a Mandatory Human Papillomavirus (HPV) Vaccination Program. J Am Board Fam Med [Internet]. 2010;23(2):220–9. | Non-European |
| Cheruvu VK, Bhatta MP, Drinkard LN. Factors associated with parental reasons for &quot;no-intent&quot; to vaccinate female adolescents with human papillomavirus vaccine: National Immunization Survey - Teen 2008-2012. BMC Pediatr [Internet]. 2017 Dec 13;17(1):52. | Non-European |
| Lees AC, Desousa N, Bailey SL, Coyne-Beasley T. 2. Parents’ Knowledge and Perceptions of HPV Male Outcomes and Intention to Vaccinate Sons. J Adolesc Heal. 2011;48(2):S5–S5 | Non-European |
| Stupiansky N, Zimet G, Cummings T, Fortenberry J, Shew M. Accuracy of self-reported HPV Vaccine Receipt Among Adolescent Girls and Their Mothers. J Adolesc Heal. 2012;50(1):103–5. | Non-European |
| Mahbubur Rahman, Lee B. Elam, Michael I. Balat, and Abbey B. Berenson.Association between well-woman visits of mothers and human papillomavirus (HPV) vaccination intent and uptake among their 9-17-year-old children. Vaccine. 2013 November 12; 31(47): 5544–5548. | Non-European |
| Hertweck SP, LaJoie AS, Pinto MD, Flamini L, Lynch T, Logsdon MC. Health Care Decision Making by Mothers for their Adolescent Daughters Regarding the Quadrivalent HPV Vaccine. J Pediatr Adolesc Gynecol. 2013;26(2):96–101. | Non-European |
| Kristin L. Johnson, Meng-Yun Lin, oward Cabral, Lewis E. Kazis IT katz. Variation in Human Papillomavirus Vaccine Uptake and Acceptability Between Female and Male Adolescents and Their caregivers. J Community Health. 2017;42(3):522–32. | Non-European |
| Marlee Grabiel, Thomas J. Reutzel, Sheila Wang, Rochelle Rubin,  Vinvia Leung, Adrienne Ordonez, Maggie Wong • Emily Jordan HPV and HPV Vaccines: The Knowledge Levels, Opinions, and Behavior of Parents. J Community Health DOI 10.1007/s10900-013-9725-6 | Non-European |
| Forster AS, McBride KA, Davies C, Stoney T, Marshall H, McGeechan K, et al. Development and validation of measures to evaluate adolescents’ knowledge about human papillomavirus (HPV), involvement in HPV vaccine decision-making, self-efficacy to receive the vaccine and fear and anxiety. Public Health. 2017 Jun [cited 2018 Mar 1];147:77–83. | Non-European |
| S.L. Davlin, A.B. Berenson, and M. Rahman. Correlates of HPV knowledge among low-income, minority mothers with a child 9 – 17 years of age. J Pediatr Adolesc Gynecol. 2015 February; 28(1): 19–23. | Non-European |
| Roberts JR, Thompson D, Rogacki B, Hale JJ, Jacobson RM, Opel DJ, et al. Vaccine hesitancy among parents of adolescents and its association with vaccine uptake. Vaccine. 2015;33(14):1748–55. | Non-European |
| Gargano LM, Weiss P, Underwood NL, Seib K, Sales JM, Vogt TM, et al. School-located vaccination clinics for adolescents: Correlates of acceptance among parents. J Community Health. 2015;40(4):660–9. | Non-European |
| Cunningham J, Wallston KA, Wilkins CH, Hull PC, Miller ST. Development and Psychometric Evaluation of the HPV Clinical Trial Survey for Parents (CTSP-HPV) Using Traditional Survey Development Methods and Community Engagement Principles. Clin Transl Sci. 2015;8(6):702–9. | Non-European |
| Clark SJ, Cowan AE, Filipp SL, Fisher AM, Stokley S. Parent HPV vaccine perspectives and the likelihood of HPV vaccination of adolescent males. Hum Vaccines Immunother. 2016;12(1):47–51. | Non-European |
| Brueggmann D, Opper N, Felix J, Groneberg DA, Mishell DR, Jaque JM. Development of a Cost-Effective Educational Tool to Promote Acceptance of the HPV Vaccination by Hispanic Mothers. J Community Health. 2016;41(3):468–75. | Non-European |
| Nickel B, Dodd RH, Turner RM, Waller J, Marlow L, Zimet G, et al. Factors associated with the human papillomavirus (HPV) vaccination across three countries following vaccination introduction. Prev Med Reports. 2017;8(October):169–76. | Non-European |
| Clark SJ, Cowan AE, Filipp SL, Fisher AM, Stokley S. Parent Perception of Provider Interactions Influences HPV Vaccination Status of Adolescent Females. Clin Pediatr (Phila). 2016;55(8):701–6. | Non-European |
| Schuler CL, Coyne-Beasley T. Has Their Son Been Vaccinated? Beliefs About Other Parents Matter for Human Papillomavirus Vaccine. Am J Mens Health. 2014;10(4):318–24. | Non-European |
| Cunningham-Erves J, Talbott LL, O’Neal MR, Ivankova N V., Wallston KA. Development of a Theory-based, Sociocultural Instrument to Assess Black Maternal Intentions to Vaccinate Their Daughters Aged 9 to 12 Against HPV. J Cancer Educ. 2016;31(3):514–21. | Non-European |
| Dempsey AF, Butchart A, Singer D, Clark S, Davis M. Factors associated with parental intentions for male human papillomavirus vaccination: Results of a national survey. Sex Transm Dis. 2011;38(8):769–76. | Non-European |
| Leader AE, Weiner JL, Kelly BJ, Hornik RC, Cappella JN. Effects of Information Framing on Human Papillomavirus Vaccination. J Women’s Health. 2009;18(2):225–33. | Non-European |
| Juraskova I, O’Brien M, Mullan B, Bari R, Laidsaar-Powell R, McCaffery K. HPV vaccination and the effect of information framing on intentions and behaviour: An application of the theory of planned behaviour and moral norm. Int J Behav Med. 2012;19(4):518–25. | Non-European |
| Joseph NP, Bernstein J, Pelton S, Belizaire M, Goff G, Horanieh N, et al. Brief Client-Centered Motivational and Behavioral Intervention to Promote HPV Vaccination in a Hard-to-Reach Population: A Pilot Randomized Controlled Trial. Clin Pediatr (Phila). 2016;55(9):851–9. | Non-European |
| Kennedy A, Sapsis KF, Stokley S, Curtis CR, Gust D. Parental attitudes toward human papillomavirus vaccination: Evaluation of an educational intervention, 2008. J Health Commun. 2011;16(3):300–13. | Non-European |
| Dempsey AF, Zimet GD, Davis RL, Koutsky L. Factors That Are Associated With Parental Acceptance of Human Papillomavirus Vaccines: A Randomized Intervention Study of Written Information About HPV. Pediatrics. 2006 May 1 [cited 2018 Feb 20];117(5):1486–93. | Non-European |
| Tuellsa J, Torrijos JL, Rosell E, Villalba E, Antonio Portero Alonso, Carmen Navarro Ortiz y Eva María Galiana de la Villa. Noticias sobre el virus del papiloma humano y su vacuna en la prensa valenciana (2006-2011). Gac Sanit. 2013;27(4):374–377. | Spanish |
| Navarro-Illana P, Caballero P, Tuells J, Puig-Barberá J, Diez-Domingo J. Acceptability of human papillomavirus vaccine in mothers from Valencia (Spain). An Pediatría (English Ed. 2015;83(5):318–27. | Spanish |
| Caballero-Pérez P, Tuells J, Rementería J, Nolasco A, Navarro-López V, Arístegui J. [Acceptability of the HPV vaccine among Spanish university students in the pre-vaccine era: a cross-sectional study]. Rev Esp Quimioter. 2015 Feb [cited 2018 Mar 1];28(1):21–8. | Spanish |
| Vielot NA, Goldberg SK, Zimet G, Smith SB, Anne McDonald M, Ramos S, et al. Acceptability of multipurpose human papillomavirus vaccines among providers and mothers of adolescent girls: A mixed-methods study in five countries. Papillomavirus Res. 2017;3(November 2016):126–33. | Spanish |

**SM-4- Knowledge of HPV by gender and vaccination status of respondent**

| **Item of knowledge of HPV** | **Study** | **Gender of respondent** | | | **Vaccination status of respondent** | | |
| --- | --- | --- | --- | --- | --- | --- | --- |
|  |  | **female adolescents (% Yes responses)** | **male adolescents (% Yes responses)** | **p- value** | **Vaccinated (% Yes responses)** | **Unvaccinated (% Yes responses)** | **p- value** |
| Do you know what HPV is? | Vaidakis D et al. (2017) | 47.9 | 35.5 | p<0.001 |  |  |  |
| Ever heard of HPV | Gottvall M et al. (2009) | 16.40 | 9.60 | p=0.017 |  |  |  |
|  | Pelucchi et al. (2010) (75) | 71.6 | 51.2 | p<0.001 |  |  |  |
|  | Marek E et al. (2011) * | 43.2 | 25.5 | p<0.001 |  |  |  |
|  | Bowyer HL et al. (2013) |  |  |  | 86.8 | 73.8 | p=0.017 |
| Have you heard about HPV? | Patel H et al. (2017) | 35.6 | 8.1 | p<0.001 |  |  |  |
|  | Maier C et al. (2015) | 57.54 | 48.08 | p<0.02 |  |  |  |
|  | Sopracordevole F et al. (2012) | 92.8% | 51.3% | p<0.01 |  |  |  |
|  | Grandahl M et al. (2017) |  |  |  | 38 | 27 | p=0.02 |
| Level of knowledge >4 (on a scale from 1 to 5) | Voidăzan et al. (2016) ** | 35.7 | 24.9 | p<0.001 |  |  |  |
| Heard about vaccine against cervical cancer | Grandahl M et al. (2017) |  |  |  | 99 | 92 | p<0.01 |
| Cervical cancer is related to HPV infection | Sopracordevole F et al. (2013) |  |  |  | 96.1 | 91.8 | p=0.045 |

Figures calculated from Table 1 in Marek E et al. (2011) **Figures calculated from Table 1 in Voidăzan et al. (2016)

**SM-5-** **Studies showing factors associated with parental and adolescent´s HPV knowledge**

| **Variables associated with knowledge of HPV** | **Subgroup reporting higher knowledge** | **OR (IC)/p-value** | **Studies** | **Respondent population** | **Multivariate or bivariate analysis** | **Number of studies with significant results / Number of studies that analyse this factor** |
| --- | --- | --- | --- | --- | --- | --- |
|  | | | |  |  | **20** |
| **Sociodemographic and family characteristics** | | | | | | |
| Gender of respondent | Females | P=0.007 | Patel H et al. (2017) | Adolescents (male & female) 18 (16-21) y.o. | Bivariate analysis | 9 / 10 |
|  |  |  |  |  |  |  |
|  | Females | P=0.004 | Pelucchi et al. (2010) | Adolescents (male & female) & parents 14-19 y.o. | Bivariate analysis |  |
|  | Females | P=0.001 | Voidăzan et al. (2016) | Parents (mother & father) of children (male and female) 7-10 y.o. | Bivariate analysis |  |
|  | Males (boys) | *0.60 (0.50-074) p<0.001* | Vaidakis D et al. (2017) | Adolescents (male & female) 17-18 y.o. | Multivariate analysis |  |
|  | Females | 6.45 (1.13-36.81) p<0.02 | Maier C et al. (2015) | Adolescents (male & female) 16-18 y.o. | Multivariate analysis |  |
|  |  |  |  |  |  |  |
|  | Females | P<0.001 | Sopracordevole F et al. (2012) | Adolescents (male & female) 16 y.o. | Bivariate analysis |  |
|  | Females | P<0.001 | Marek E et al. (2011) | Adolescents (male & female) 12-19 y.o. | Bivariate analysis |  |
|  | Females (girls) | P<0.001 | Balemans R. et al. (2011) | Adolescents (male & female) 14-17 y.o. | Bivariate analysis |  |
|  | Females | p=0.017 | Gottvall M et al. (2009) | Adolescents (male & female) 15-16 y.o. | Bivariate analysis |  |
| Nationality | Italian (local) | 3.03 (1.55-5.91) | Giambi C et al. (2014) | Parents (mother & father) of female children 12-14 y.o. | Multivariate analysis | 3 / 4 |
|  |  | 2.3 (1.2-4.4) | Chadenier GMC et al. (2011) | Mothers of female children 12 y.o. | Multivariate analysis |  |
|  | Spanish (local) | P=0.015 | Navarro- Illana P et al. (2014) | Female adolescents 15 y.o. | Bivariate analysis |  |
| Geographical area | North of the country | 1.79 (1.17-2.75) | Giambi C et al. (2014) | Parents (mother & father) of female children 12-14 y.o. | Multivariate analysis | 1 / 2 |
|  |  |  |  |  |  |  |
|  | Centre of the country | 1.73 (1.04-2.89) |  |  |  |  |
| Marital status | Unmarried or cohabiting | P=0.003 | Voidăzan et al. (2016) | Parents (mother & father) of children (male and female) 7-10 y.o. | Bivariate analysis | 1 / 2 |
| Educational Status | Higher | P<0.001 | Voidăzan et al. (2016) | Parents (mother & father) of children (male and female) 7-10 y.o. | Bivariate analysis | 5 / 6 |
|  |  | 3.10 (2.33-4.15) | Giambi C et al. (2014) | Parents (mother & father) of female children 12-14 y.o. | Multivariate analysis |  |
|  |  | **1.55 (1.12-2.16)** | **Bianco AS et al. (2014)** | **Parents (mother & father) of male children 10-14 y.o.** | **Multivariate analysis** |  |
|  |  | P<0.001 | Marek E et al. (2011) | Adolescents (male & female) 12-19 y.o. | Bivariate analysis |  |
|  |  | 2.4 (1.6-3.6) | Chadenier GMC et al. (2011) | Mothers of female children 12 y.o. | Multivariate analysis |  |
| Type of school | General education vs technical education | P=0.001 | Balemans R. et al. (2011) | Adolescents (male & female) 14-17 y.o. | Bivariate analysis | 1 / 1 |
| Role of religion in your life | Important role | 1.32 (1.07-0.62) p<0.001 | Vaidakis D et al. (2017) | Adolescents (male & female) 17-18 y.o. | Multivariate analysis | 1 / 1 |
| Age | Higher age of respondent parent | 1.1 (1.0-1.2) | Tozzi A et al. (2009) | Mothers of female children 10-12 y.o. | Multivariate analysis | 1 / 2 |
| Having older sisters who had received HPV vaccine | Yes | P=0.016 | Navarro- Illana P et al. (2014) | Female adolescents 15 y.o. | Bivariate analysis | 1 / 1 |
| Monthly household income | High income | 5.82 (1.10-33.29) /p=0.04 | Maier C et al. (2015) | Adolescents (male & female) 16-18 y.o. | Multivariate analysis | 1 / 2 |
| Ever had sex | No | *0.59 (0.38- 0.91)* | Samkange-Zeeb F et al. (2012) | Female adolescents 12-20 y.o. | Multivariate analysis | 1 / 1 |
| Age at first intercourse | 16-18 vs 12-15 | 1.33 (1.01-1.75) P=0.046 | Vaidakis D et al. (2017) | Adolescents (male & female) 17-18 y.o. | Multivariate analysis | 2 / 2 |
|  |  | 6.15 (1.07-5.14)/p=0.03 | Maier C et al. (2015) | Adolescents (male & female) 16-18 y.o. | Multivariate analysis |  |
| Good relationship with family | Yes | p=0.04 | Maier C et al. (2015) | Adolescents (male & female) 16-18 y.o. | Multivariate analysis | 1 / 1 |
| **Attitudes, beliefs and other factors associated with higher knowledge of HPV** | | | | | | |
| Number of sources of information | >4 | 3.96 (2.84-5.53) | Giambi C et al. (2014) | Parents (mother & father) of female children 12-14 y.o. | Multivariate analysis | 1 / 1 |
| Physician as source of information | **Yes** | **1.71 (1.12-2.61)** | **Bianco AS et al. (2014)** | **Parents (mother & father) of male children 10-14 y.o.** | **Multivariate analysis** | 2 / 2 |
|  | Yes | P<0.001 | Voidăzan et al. (2016) | Parents (mother & father) of children (male and female) 7-10 y.o. | Bivariate analysis |  |
| Are you registered with a GP? | Yes | P=0.01 | Voidăzan et al. (2016) | Parents (mother & father) of children (male and female) 7-10 y.o. | Bivariate analysis | 1 / 1 |
| Have you asked your GP for information about HPV infection? | Yes | P<0.001 | Voidăzan et al. (2016) | Parents (mother & father) of children (male and female) 7-10 y.o. | Bivariate analysis | 1 / 1 |
| Vaccination status | Yes | p=0.02 | Grandahl M et al. (2017) | Adolescents (male & female) 16 y.o. | Bivariate analysis | 4 / 4 |
|  |  |  | Navarro- Illana P et al. (2014) | Female adolescents 15 y.o. | Bivariate analysis |  |
|  |  | P=0.05 |  |  |  |  |
|  |  | p<0.05 | Sopracordevole F et al. (2013) | Adolescents (male & female) 16 y.o. | Bivariate analysis |  |
|  |  | p=0.017 | Bowyer HL et al. (2013) | Female adolescents 16-17 y.o. | Bivariate analysis |  |
| Knowledge that HPV is transmitted by sexual intercourse | **Yes** | **5.83 (1.95-17.44)** | **Bianco AS et al. (2014)** | **Parents (mother & father) of male children 10-14 y.o.** | **Multivariate analysis** | **1 / 1** |
| Knowledge that risk of HPV infection is related to both sexes | **Yes** | **1.98 (1.15-3.42)** | **Bianco AS et al. (2014)** | **Parents (mother & father) of male children 10-14 y.o.** | **Multivariate analysis** | **1 / 1** |
|  |  |  |  |  |  |  |
| Intention to vaccinate daughter against HPV infection | Yes | P=0.02 | Voidăzan et al. (2016) | Parents (mother & father) of children (male and female) 7-10 y.o. | Bivariate analysis | 1 / 1 |
| Discussing with peers about HPV vaccine | Yes | Correlation p<0.05 | Navarro- Illana P et al. (2014) | Female adolescents 15 y.o. | Bivariate analysis | 1 / 1 |
| Perceived severity | Greater | R=0.29 p<0.01 | De Visser R et al. (2008) | Parents (mother & father) of children (male and female) 12-13 y.o. | Bivariate analysis | 1 / 1 |
| Concern about HPV | Greater | R=0.26 p<0.01 | De Visser R et al. (2008) | Parents (mother & father) of children (male and female) 12-13 y.o. | Bivariate analysis | 1 / 1 |
| Subjective norms supportive of HPV vaccination | Greater | R=0.15 p<0.01 | De Visser R et al. (2008) | Parents (mother & father) of children (male and female) 12-13 y.o. | Bivariate analysis | 1 / 1 |
| Support for provision of adolescent sexual health services | Greater | R=0.18 p<0.01 | De Visser R et al. (2008) | Parents (mother & father) of children (male and female) 12-13 y.o. | Bivariate analysis | 1 / 1 |
| Mother's positive attitude toward Pap Test | Yes | 2.66 (1.85-3.84) | Giambi C et al. (2014) | Parents (mother & father) of female children 12-14 y.o. | Multivariate analysis | 1 / 2 |

In dark grey, factors related to parents’ knowledge about HPV in males

*In italics, factors negatively associated with knowledge about HPV*

**SM-6- Studies showing variables associated with parental and adolescent HPV vaccine acceptability.**

| **Variables associated with HPV vaccine acceptability** | **Subgroup reporting higher acceptance** | **OR (95% CI)/p** | **Studies** | **Respondent population** | **Multivariate or bivariate analysis** | **Number of studies with significant results / Number of studies that analyse this factor** |
| --- | --- | --- | --- | --- | --- | --- |
|  |  |  |  |  |  | **37** |
| **Sociodemographic/ family characteristics /factual conditions** | | | | | | |
| Gender of respondent | Females | 13 (1.19-141.47) | Maier C et al. (2015) | Adolescents (male & female) 16-18 y.o. | Multivariate analysis | 4 / 5 |
|  | Mothers & female adolescents | P=0.03/ p=0.001 | Pelucchi C et al. (2010) | Adolescents (male & female) & parents 14-19 y.o. | Multivariate analysis |  |
|  | Females (girls) | P<0.001 | Gottvall M et al. (2009) | Adolescents (male & female) 15-16 y.o. | Bivariate analysis |  |
|  | Females | P<0.019 | Lenselink CH et al. (2008) | Parents (mother & father) of children (male and female) 10-12 y.o. | Bivariate analysis |  |
| Age of respondent parent | 30-49 | Daughter: 1.3 (1.0-1.6) | Agorastos et al. (2015) | Mothers of children (male & females) 13 y.o. | Multivariate analysis | 4 / 9 |
|  | 30-39 | Son: 1.3 (1.0-1.6) | Agorastos et al. (2015) | Mothers of children (male & females) 13 y.o. | Multivariate analysis |  |
|  | High vs low | 2.19 (1.16-4.15)/p=0.02 | Stöcker P et al. (2013) | Female adolescents 15 (14-18) y.o. | Multivariate analysis |  |
|  | >45 | *0.78 (0.66-0.91) (if vaccine is free)* | Dahlström L et al. (2010) | Parents (mother & father) of children (male and female) 12-15 y.o. | Multivariate analysis |  |
| Family situation | Separated | *0.33 (0.13-0.81)/p=0.016* | Navarro-Illana P et al (2017) | Female adolescents & Parents of female children 12-16 | Multivariate analysis | 4 / 6 |
|  | Married | 2.2 (1.3-3.6)/p=0.002 | Navarro-Illana P et al. (2015) | Mothers of female children 12-16 y.o. | Multivariate analysis |  |
|  | Single | Son: 1.3 (1.0-1.6) | Agorastos et al. (2015) | Mothers of children (male & females) 13 y.o. | Multivariate analysis |  |
|  | Single | 1.55 (1.26-1.91) (if vaccine is free) | Dahlström L et al. (2010) | Parents (mother & father) of children (male and female) 12-15 y.o. | Multivariate analysis |  |
|  |  | 1.31 (1.11-1.54) (if it is not free) |  |  |  |  |
| Residence | Rural | Daughter: 1.5 (1.2-1.9) | Agorastos et al. (2015) | Mothers of children (male & females) 13 y.o. | Multivariate analysis | 3 / 4 |
|  | Rural | Son: 1.6 (1.3-2.0) | Agorastos et al. (2015) | Mothers of children (male & females) 13 y.o. | Multivariate analysis |  |
|  | >1000 inhabitants | *0.8 (0.7-0.9) /p=0.004* | Mollers M et al. (2014) | Female adolescents 16-17 y.o. | Multivariate analysis |  |
| Nationality /Native | Foreign | *0.49 (0.24-0.98)/p=0.042* | Navarro-Illana P et al (2017) | Female adolescents & Parents of female children 12-16 | Multivariate analysis | 7 / 8 |
|  | Greek (local) | Daughter: 3.3 (2.3-4.7) | Agorastos et al. (2015) | Mothers of children (male & females) 13 y.o. | Multivariate analysis |  |
|  |  | Son: 3.3 (2.3-4.8) |  |  |  |  |
|  | Spanish (local) | 2.0 (1.2-3.3)/p=0.008 | Navarro-Illana P et al. (2015) | Mothers of female children 12-16 y.o. | Multivariate analysis |  |
|  | Not native (Sweden) | *0.77 (0.63-0.93) (if vaccine is not free)* | Dahlström L et al. (2010) | Parents (mother & father) of children (male and female) 12-15 y.o. | Multivariate analysis |  |
|  | Italian (local) | 4.0 (1.6-10.5) | Chadenier GMC et al. (2011) | Mothers of female children 12 y.o. | Multivariate analysis |  |
|  | Not native | *0.35 (0.17-0.45)/ p=0.005* | Marlow et al. (2009) | Female adolescents 16-19 y.o. | Bivariate / Multivariate analysis |  |
|  | **France vs. UK, Germany, Italy** | **p<0.001** | **Lee Mortenssen et al. (2015)** | **Parents (mother & father) of male children 12-17 y.o.** | **Bivariate** |  |
| Educational Status | High School/ University | 1.75 (1.13-2.72) | Borena W et al. (2016) | Parents (mother & father) of children (male and female) 9-10 y.o. | Multivariate analysis | 6 / 10 |
|  | Higher | 1.5 (1.0-2.3) | Schülein S et al. (2016) | Female adolescents 9-17 y.o. | Multivariate analysis |  |
|  | Intermediate | 1.5 (1.1-2.1) |  |  |  |  |
|  | High school | *Daughter: 0.7 (0.6-0.9)* | Agorastos et al. (2015) | Mothers of children (male & females) 13 y.o. | Multivariate analysis |  |
|  |  | *Son: 0.7 (0.6-0.8)* |  |  |  |  |
|  | Higher | 2.9 (1.3-6.6) | Chadenier GMC et al. (2011) | Mothers of female children 12 y.o. | Multivariate analysis |  |
|  | High school | *0.75 (0.62-0.91) (if vaccine is free)* | Dahlström L et al. (2010) | Parents (mother & father) of children (male and female) 12-15 y.o. | Multivariate analysis |  |
|  |  | *0.79 (0.68-0.92) (even if not free)* |  |  |  |  |
|  | University degree | P<0.001 | Lenselink CH et al. (2008) | Parents (mother & father) of children (male and female) 10-12 y.o. | Bivariate analysis |  |
| Profession /employment | Housewife/farmer | Daughter: 0.8 (0.7-1.0) | Agorastos et al. (2015) | Mothers of children (male & females) 13 y.o. | Multivariate analysis | 2 / 4 |
|  | Unemployed | 1.31 (1.09-1.56) (if vaccine is free) | Dahlström L et al. (2010) | Parents (mother & father) of children (male and female) 12-15 y.o. | Multivariate analysis |  |
| Monthly household income | High | 7.11 (1.12-44.91)/p=0.01 | Maier C et al. (2015) | Adolescents (male & female) 16-18 y.o. | Multivariate analysis | 3 / 5 |
|  | <1,000 (€) | Son: 0.7 (0.5-0.9) | Agorastos et al. (2015) | Mothers of children (male & females) 13 y.o. | Multivariate analysis |  |
|  | Higher | *0.71 (0.56-0.91) (if vaccine is free)* | Dahlström L et al. (2010) | Parents (mother & father) of children (male and female) 12-15 y.o. | Multivariate analysis |  |
|  |  | 1,32 (1,11-1,58) (if vaccine is not free) |  |  |  |  |
| Religion | None | *0.90 (0.87-0.94) /P=0.02* | Bowyer et al. (20134) | Female adolescents 15-16 y.o. | Multivariate analysis | 6 / 8 |
|  | Catholic | 1.2 (1.0-1.5) | Mollers M et al. (2014) | Female adolescents 16-17 y.o. | Multivariate analysis |  |
|  | Protestant Christian | *0.4 (0.4-0.5)* |  |  |  |  |
|  | Other | *0.3 (0.1-0.7)* |  |  |  |  |
|  | Religious conviction | 2.17 (1.09-4.40) /p=0.03 | Gefenaite et al. (2012) | Parents (mother & father) of female children 13-16 y.o. | Multivariate analysis |  |
|  | Other than catholic | *0.61 (0.46-0.82)* | Pelucchi C et al. (2010) | Adolescents (male & female) & parents 14-19 y.o. | Multivariate analysis |  |
|  | Moslem | *0.19 (0.06-0.59) /p=0.004* | Marlow et al. (2009) | Female adolescents 16-19 y.o. | Bivariate / Multivariate analysis |  |
|  | Hindu | *0.12 (0.30-0.45) /p=0.002* |  |  |  |  |
|  | Other than Christian | *0.32 (0.11-0.93) /p=0.036* | Marlow L et al (2007) | Mothers of female children 11 (8-14) y.o. | Multivariate analysis |  |
| Ethnicity | NL vs SNA, MENA & others | p<0.001 | Alberts CJ et al. (2017) | Parents (mother & father) of female children 13 y.o. | Multivariate analysis | 4 / 6 |
|  | Black | *0.19 (0.04-0.98) /p=0.048* | Bowyer et al. (2014) | Female adolescents 15-16 y.o. | Multivariate analysis |  |
|  | Asian | *0.25 (0.07-0.888)/p=0.038* |  |  |  |  |
|  | Asian | P=0.039 | Marlow et al. (2009) | Female adolescents 16-19 y.o. | Bivariate / Multivariate analysis |  |
|  | White and Black Caribbean | P<0.001 | Brabin L et al. (2007) | Parents (mother & father) of children (male and female) 11-12 y.o. |  |  |
| Cultural/religious perspectives (ethnic & religious minority groups) | Yes | *0.09 (0.03-0.26)/ p<0.001* | Brabin L et al. (2006) | Parents (mother & father) of children (male and female) 11-12 y.o. | Multivariate analysis | 1 / 1 |
| Gender of child | At least one daughter | 1.88 (1.53-2.30) | Pelucchi C et al. (2010) | Adolescents (male & female) & parents 14-19 y.o. | Multivariate analysis | 3 / 3 |
|  | Female | 1.35 (1.22-1.50) | Dahlström L et al. (2010) | Parents (mother & father) of children (male and female) 12-15 y.o. | Multivariate analysis |  |
|  | Female | p<0.001 | de Visser E et al. (2008) | Parents (mother & father) of children (male and female) 12-13 y.o. | Multivariate analysis |  |
| Number of children | >2 | *0.81 (0.70-0.94) (if vaccine is not free)* | Dahlström L et al.(2010) (76) | Parents (mother & father) of children (male and female) 12-15 y.o. | Multivariate analysis | 1 / 4 |
| Age of child/adolescent | Higher | 1.15 (1.04-1.27) /p=0.007 | Marlow L et al (2007) | Mothers of female children 11 (8-14) y.o. | Multivariate analysis | 2 / 6 |
|  | Higher | 1.6 (1.5-1.7) | Schülein S et al. (2016) (31) | Female adolescents 9-17 y.o. | Multivariate analysis |  |
| Age at first sexual intercourse >16 years | Yes | 17.98 (1.72-187.7)/p=0.03 | Maier C et al. (2015) | Adolescents (male & female) 16-18 y.o. | Multivariate analysis | 1 / 1 |
| Vaccinated against pneumococcus/previous childhood vaccinations | Yes | p=0.025 | Grandahl M et al. (2017) | Parents (mother & father) of female children 11-12 y.o. | Bivariate analysis | 3 / 3 |
|  | Yes | 15.8 (6.62-37.8) | Borena W et al. (2016) | Parents (mother & father) of children (male and female) 9-10 y.o. | Multivariate analysis |  |
|  | Yes | 3.248 (1.32-8.11) /p=0.010 | Haesebaert J et al. (2012) | Mothers of female children 14-18 y.o. | Multivariate analysis |  |
| Good relationship with family | Yes | P=0.03 | Maier C et al. (2015) | Adolescents (male & female) 16-18 y.o. | Multivariate analysis | 1 / 1 |
| Alcohol use | Yes | 1.4 (1.2-1.7) | Mollers M et al. (2014) | Female adolescents 16-17 y.o. | Multivariate analysis | 1 / 1 |
| Contraception | Yes | 1.5 (1.3-1.8) | Mollers M et al. (2014) | Female adolescents 16-17 y.o. | Multivariate analysis | 1 / 1 |
| Has had sex | Yes | 1.2 (1.0-1.4) | Mollers M et al. (2014) | Female adolescents 16-17 y.o. | Multivariate analysis | 1 / 3 |
| Total number of lifetime sexual partners | Mean number | *0.9 (0.9-1.0) /p<0.001* | Mollers M et al. (2014) | Female adolescents 16-17 y.o. | Multivariate analysis | 1 / 1 |
| **Drivers/Reasons for accepting HPV vaccination** | | | | | | |
| Approval of vaccines as preventive method/better to vaccinate as many people as possible to protect the unvaccinated | Yes | 4.1 (3.3-5.3) | Navarro-Illana P et al. (2015) | Mothers of female children 12-16 y.o. | Multivariate analysis | 2 / 2 |
|  |  | P=0.001 | Voidăzan S et al. (2016) | Parents (mother & father) of children (male and female) 7-10 y.o. | Multivariate analysis |  |
| Vaccines are effective at preventing disease/ convinced of efficacy/HPV efficacy | Yes | P= 0.004 | Grandahl M et al. (2017) | Parents (mother & father) of female children 11-12 y.o. | Bivariate analysis | 8 / 8 |
|  | No | *0.39 (0.23-0.64) (if vaccine is free)* | Dahlström L et al. (2010) | Parents (mother & father) of children (male and female) 12-15 y.o. | Multivariate analysis |  |
|  |  | *0.24 (0.17-0.36) (if not free)* |  |  |  |  |
|  | Yes | R=0.64 (p<0.01) | Morison LA et al. (2010) | Parents (mother & father) of female children 11-12 y.o. | Bivariate analysis |  |
|  | Yes | 1.46 (1.231.72)/p<0.001 | Marlow et al. (2009) | Female adolescents 16-19 y.o. | Multivariate analysis |  |
|  | Yes | Beta=0.35/p<0.01 Girls | de Visser E et al. (2008) | Parents (mother & father) of children (male and female) 12-13 y.o. | Multivariate analysis |  |
|  |  | Beta=0.40/p<0.01 Boys |  |  |  |  |
|  | Very clearly or clearly | P=0.01 | Stretch R et al. (2008) | Parents (mother & father) of female children 12-13 y.o. | Bivariate analysis |  |
|  | Yes | 1.29 (1.19-1.39)/p<0.001 | Marlow et al. (2007) | Mothers of female children 11 (8-14) y.o. | Multivariate analysis |  |
|  | Yes | 51.8 (16.0-167.8)/p<0.001 | Brabin L et al. (2006) | Parents (mother & father) of children (male and female) 11-12 y.o. | Multivariate analysis |  |
| Belief in the safety of vaccines | Yes | Beta=0.29/p<0.01 Girls | de Visser E et al. (2008) | Parents (mother & father) of children (male and female) 12-13 y.o. | Multivariate analysis | 1 / 1 |
| Severity of the disease | Yes | P<0.05 | Balla B et al. (2016) | Female adolescents 18-19 y.o. | Bivariate analysis | 3 / 3 |
|  |  | p=0.02 | Grandahl M et al. (2017) | Adolescents (male & female) 16 y.o. | Bivariate analysis |  |
|  |  | 1.15 (1.05-1.26)/p=0.003 | Marlow L et al (2007) | Mothers of female children 11 (8-14) y.o. | Multivariate analysis |  |
| Heard of HPV vaccine/previous awareness of HPV /previous knowledge of the virus | Yes | 1.74 (1.10-2.79) | Borena W et al. (2016) | Parents (mother & father) of children (male and female) 9-10 y.o. | Multivariate analysis | 6 / 6 |
|  | Yes | 8.12 (1.13-38.1)/p=0.004 | Haesebaert J et al. (2012) | Mothers of female children 14-18 y.o. | Multivariate analysis |  |
|  | **Yes** | **P= 0.002** | **Forster AS et al. (2012)** (66) | **Male adolescents 16-18 y.o.** | **Bivariate analysis** |  |
|  | Yes | 1.42 (1.21-1.66) (if vaccine is free) | Dahlström L et al. (2010) | Parents (mother & father) of children (male and female) 12-15 y.o. | Multivariate analysis |  |
|  |  | 1.96 (1.75-2.20) (even if not free) |  |  |  |  |
|  | Yes | Value of p not provided | Gottvall M et al. (2009) | Adolescents (male & female) 15-16 y.o. | Bivariate analysis |  |
|  | Higher knowledge score | 1.29 (1.19-1.39) | Marlow et al. (2007) | Mothers of female children 11 (8-14) y.o. | Multivariate analysis |  |
| Respondent's estimation of high HPV prevalence | Yes | 1.80 (1.20-2.68) | Borena W et al. (2016) | Parents (mother & father) of children (male and female) 9-10 y.o. | Multivariate analysis | 1 / 1 |
| Perceived benefits of HPV vaccine | **Yes (perceived benefits in girls)** | **1.49 (1.24-1.79)/p<0.001** | **Bianco AS et al. (2014)** | **Parents (mother & father) of male children 10-14 y.o.** | **Multivariate analysis** | 4 / 4 |
|  | Yes | Beta=0.16; p<0.01 Girls | de Visser E et al. (2008) | Parents (mother & father) of children (male and female) 12-13 y.o. | Multivariate analysis |  |
|  |  | Beta=0.15; p<0.01 Boys |  |  |  |  |
|  | Yes | 2.2 (1.7-2.9) | Mollers M et al. (2014) | Female adolescents 16-17 y.o. | Multivariate analysis |  |
|  | Yes | P=0.004 | Stretch R et al. (2008) | Parents (mother & father) of female children 12-13 y.o. | Bivariate analysis |  |
| Perceived benefits of vaccinations within the childhood immunization program (acceptability for males) | **Yes** | **1.28 (1.05-1.55)** | **Bianco AS et al. (2014)** | **Parents (mother & father) of male children 10-14 y.o.** | **Multivariate analysis** | 1 / 1 |
| Confidence in the HPV vaccination/belief that it is necessary | Yes | P<0.001 | Grandahl M et al. (2017) | Parents (mother & father) of female children 11-12 y.o. | Bivariate analysis | 2 / 2 |
|  |  | P=0.001 | Voidăzan S et al. (2016) | Parents (mother & father) of children (male and female) 7-10 y.o. | Multivariate analysis |  |
| HPV vaccination protects against cervical cancer | Yes | p<0.01 | Grandahl M et al. (2017) | Adolescents (male & female) 16 y.o. | Bivariate analysis | 6 / 6 |
|  |  | p<0.001 | Grandahl M et al. (2017) | Parents (mother & father) of female children 11-12 y.o. | Bivariate analysis |  |
|  |  | P=0.001 | Voidăzan S et al. (2016) | Parents (mother & father) of children (male and female) 7-10 y.o. | Multivariate analysis |  |
|  |  | p<0.05 | Balla B et al. (2016) | Female adolescents 18-19 y.o. | Bivariate analysis |  |
|  |  | P<0.05 | Marek E et al. (2011) | Adolescents (male & female) 12-19 y.o. | Bivariate analysis |  |
|  |  | 2.,31 (1.69-3.16) | Pelucchi C et al. (2010) | Adolescents (male & female) & parents 14-19 y.o. | Multivariate analysis |  |
| Possible of getting HPV in the future/susceptibility/perceived risk | Yes | p=0.02 | Grandahl M et al. (2017) | Adolescents (male & female) 16 y.o. | Bivariate analysis | 4 / 5 |
|  |  | p>0.05 | Balla B et al. (2016) | Female adolescents 18-19 y.o. | Bivariate analysis |  |
|  |  | 1.94 (1.36-2.77)/p<0.001 | Marlow et al. (2009) | Female adolescents 16-19 y.o. | Bivariate / Multivariate analysis |  |
|  |  | 1.27 (1.14-1.40)/p<0.001 | Marlow L et al (2007) | Mothers of female children 11 (8-14) y.o. | Multivariate analysis |  |
| Consideration of future consequences | Yes | R=0.16 (p<0.05) | Morison LA et al. (2010) | Parents (mother & father) of female children 11-12 y.o. | Bivariate analysis | 1 / 1 |
| Number of positive thoughts | Yes | R=0.41 (p<0.01) | Morison LA et al. (2010) | Parents (mother & father) of female children 11-12 y.o. | Bivariate analysis | 1 / 1 |
| Anticipated regret is that daughter does not have the vaccine | Yes | R=0.45 (p<0.01) | Morison LA et al. (2010) | Parents (mother & father) of female children 11-12 y.o. | Bivariate analysis | 1 / 1 |
| Has a boyfriend/girlfriend | Yes | *females 0.67 (0.47-0.97)* | Pelucchi C et al. (2010) | Adolescents (male & female) & parents 14-19 y.o. | Multivariate analysis | 2 / 2 |
|  |  | 1.18 (0.94-1.48) (if vaccine is free) | Dahlström L et al. (2010) | Parents (mother & father) of children (male and female) 12-15 y.o. | Multivariate analysis |  |
|  |  | 1.30 (1.16-1.47) (if not free) |  |  |  |  |
| Believe child has had intercourse | Yes | 1.18 (0.94-1.48) (if vaccine is free) | Dahlström L et al. (2010) | Parents (mother & father) of children (male and female) 12-15 y.o. | Multivariate analysis | 1 / 1 |
|  |  | 1.22 (1.03-1.46) (if not free) |  |  |  |  |
| Recommended for young adolescent girls before sexual debut |  | 2.12 (1.15-3.90)/p=0.016 | Haesebaert J et al. (2012) | Mothers of female children 14-18 y.o. | Multivariate analysis | 1 / 1 |
| Discussing sex at an early age |  | 1.12 (1.01-1.24)/p=0.036 | Marlow L et al (2007) | Mothers of female children 11 (8-14) y.o. | Multivariate analysis | 1 / 1 |
| Child’s opinion is important | Yes | P=0.006) | Lenselink CH et al. (2008) | Parents (mother & father) of children (male and female) 10-12 y.o. | Bivariate analysis | 1 / 1 |
| Thinks that HPV might concern him/her | Yes | males 5.73 (2.85-11.5) | P Pelucchi C et al. (2010) | Adolescents (male & female) & parents 14-19 y.o. | Multivariate analysis | 1 / 1 |
|  |  | females 2.39 (1.66-3.46) |  |  |  |  |
| Thinks that HPV might concern own child(ren) | Yes | 3.52 (2.89-4.29) | Pelucchi C et al. (2010) | Adolescents (male & female) & parents 14-19 y.o. | Multivariate analysis | 1 / 1 |
| Intend to use condom if I have sex with a new partner | Yes | p=0.01 | Grandahl M et al. (2017) | Adolescents (male & female) 16 y.o. | Bivariate analysis | 1 / 1 |
| Support for adolescent sexual health services | Yes | Beta=0.16; p<0.01 Girls | de Visser E et al. (2008) | Parents (mother & father) of children (male and female) 12-13 y.o. | Multivariate analysis | 1 / 1 |
|  |  | Beta=0.15; p<0.01 Boys |  |  |  |  |
| Experience of cancer in family | Yes | 1.61 (1.14-2.29)/p=0.007 | Marlow L et al (2007) | Mothers of female children 11 (8-14) y.o. | Multivariate analysis | 2 / 3 |
|  |  | P=0.001 | Voidăzan S et al. (2016) | Parents (mother & father) of children (male and female) 7-10 y.o. | Multivariate analysis |  |
| Pap Test at present or future intention | Yes | p=0,01 | Grandahl M et al. (2017) | Adolescents (male & female) 16 y.o. | Bivariate analysis | 3 / 3 |
|  |  | Daughter: 1.5 (1.2-1.9) | Agorastos et al. (2015) | Mothers of children (male & females) 13 y.o. | Multivariate analysis |  |
|  |  | Son: 1.4 (1.1-1.7) |  |  |  |  |
|  |  | *0.32 (0.12-0.82)/p=0.018* | Haesebaert J et al. (2012) | Mothers of female children 14-18 y.o. | Multivariate analysis |  |
| Desire to fit in social norms /believe authorities | Yes | P=0.001 | van Keulen et a. (2013) | Female adolescents & Mothers of female adolescents 13-14 y.o. | Multivariate analysis | 4 / 4 |
|  |  | Beta=0.14; p<0.01 Girls | de Visser E et al. (2008) | Parents (mother & father) of children (male and female) 12-13 y.o. | Multivariate analysis |  |
|  |  | 1.78 (1.59-2.01) | Marlow L et al (2007) | Mothers of female children 11 (8-14) y.o. | Multivariate analysis |  |
|  |  | 3.8 (2.2-6.7) /p=0.001 | Brabin L et al. (2006) | Parents (mother & father) of children (male and female) 11-12 y.o. | Multivariate analysis |  |
| Social norm- Husband will not oppose vaccination | Yes | 14.51 (6.15-34.25) /p<0.0001 | Marlow L et al (2007) | Mothers of female children 11 (8-14) y.o. | Multivariate analysis | 1 / 1 |
| Positive family/personal attitude toward recommended vaccination/HPV vaccine | Yes | P<0.01 | van Keulen et a. (2013) | Female adolescents & Mothers of female adolescents 13-14 y.o. | Multivariate analysis | 3 / 3 |
|  | Yes | R=0.69 (p<0.01) | Morison LA et al. (2010) | Parents (mother & father) of female children 11-12 y.o. | Bivariate analysis |  |
|  | Yes | P<0.05 | Balla B et al. (2016) | Female adolescents 18-19 y.o. | Bivariate analysis |  |
| Positioning of their peers about HPV vaccine | Positive | Correlation p<0.001 | Navarro-Illana P et al. (2014) | Female adolescents 15 y.o. | Bivariate analysis | 1 / 1 |
| Received information/recommendation from doctor | Yes | 1.72 (1.01-2.94) /p= 0.048 | Navarro-Illana P et al (2017) | Female adolescents & Parents of female children 12-16 | Multivariate analysis | 4 / 4 |
|  |  | P=0.009 | Voidăzan S et al. (2016) | Parents (mother & father) of children (male and female) 7-10 y.o. | Multivariate analysis |  |
|  |  | 1.60 (1.06-2.43) | Borena W et al. (2016) | Parents (mother & father) of children (male and female) 9-10 y.o. | Multivariate analysis |  |
|  |  | 1.8 (1.2-2.8)/p=0.004 | Navarro-Illana P et al. (2015) | Mothers of female children 12-16 y.o. | Multivariate analysis |  |
| Received information in pamphlets | Yes | 2.32 (1.37-3.92) /p=0.002 | Navarro-Illana P et al (2017) | Female adolescents & Parents of female children 12-16 | Multivariate analysis | 2 / 2 |
|  |  | 2.0 (1.4-3.1)/p=0.001 | Navarro-Illana P et al. (2015) | Mothers of female children 12-16 y.o. | Multivariate analysis |  |
| Received information from a nurse | Yes | 1.83 (1.01-.3.35 /p=0.003 | Navarro-Illana P et al (2017) | Female adolescents & Parents of female children 12-16 | Multivariate analysis | 2 / 2 |
|  |  | 2.1 (1.2-3.4)/p=0.006 | Navarro-Illana P et al. (2015) | Mothers of female children 12-16 y.o. | Multivariate analysis |  |
| I was advised to have the vaccine by the nurse | Yes | 6.57 (3.19-13.56) | Navarro-Illana P et al (2017) | Female adolescents & Parents of female children 12-16 | Multivariate analysis | 1 / 1 |
| Number of information sources | Number | Correlation p<0.05 | Marek E et al. (2011)( | Adolescents (male & female) 12-19 y.o. | Bivariate analysis | 1 / 1 |
| Confidence in own doctor |  | 1.70 (1.23-2.36) /p<0.01 | Marlow et al. (2007) | Mothers of female children 11 (8-14) y.o. | Multivariate analysis | 1 / 1 |
| Confidence in the government in general | Yes | P<0.01 | van Keulen et a. (2013) | Female adolescents & Mothers of female adolescents 13-14 y.o. | Multivariate analysis | 2 / 2 |
|  | Yes | 1.35 (1.22-1.50) /p<0.001 | Marlow et al. (2007) | Mothers of female children 11 (8-14) y.o. | Multivariate analysis |  |
| Has attended the nurse-led clinic in the community healthcare centre in the last year | More than once | 2.12 (1.10-4.07) /p=0.025 | Navarro-Illana P et al (2017)(26) | Female adolescents & Parents of female children 12-16 | Multivariate analysis | 1 / 1 |
| **Barriers/reasons for refusing HPV vaccination** | | | | | | |
|  |  |  |  |  |  |  |
|  |  |  |  |  |  |  |
| Too many vaccinations included in the childhood vaccination schedule | Agree | *0.35 (0.25-0.48)/p<0.001* | Marlow et al. (2007) | Mothers of female children 11 (8-14) y.o. | Multivariate analysis | 2 / 2 |
|  |  | *0.22 (0.15-0.31)/p=0.0001* | Marlow L et al (2007) | Mothers of female children 11 (8-14) y.o. | Multivariate analysis |  |
| Put off a previous vaccination | Yes | *0.31 (0.19-0.51)/p<0.001* | Marlow et al. (2007) | Mothers of female children 11 (8-14) y.o. | Multivariate analysis | 1 / 1 |
| Refused a previous vaccination | Yes | *0.33 (0.18-0.59)/p<0.001* | Marlow et al. (2007) | Mothers of female children 11 (8-14) y.o. | Multivariate analysis | 1 / 1 |
| Negative attitudes towards vaccination in general | Yes | *0.33 (0.13-0.84)/p=0.02* | Stöcker P et al. (2013) | Female adolescents 15 (14-18) y.o. | Multivariate analysis | 1 / 1 |
| Low knowledge of HPV and STDs | Yes | 3.4 (1.4-8.3) adolescents | Woodhall SC et al. (2007)(86) | Adolescents (male & female) & their parents 14-15 y.o. | Multivariate analysis | 1 / 1 |
|  |  | 4.3 (2.5-7.6) Parents |  |  |  |  |
| HPV vaccine is too new | Yes | 21.08 (2.57-172.97) | Firenze A et al. (2015) | Female adolescents 15 (13-17) y.o. | Bivariate analysis | 1 / 1 |
| Low perceived susceptibility /HPV risk/STI concerns | Yes | p=0.046 | Balla B et al. (2016) | Female adolescents 18-19 y.o. | Bivariate analysis | 3 / 3 |
|  |  | 3.0 (1.3-6.9) Parents | Woodhall SC et al. (2007) | Adolescents (male & female) & their parents 14-15 y.o. | Multivariate analysis |  |
|  |  | 7.9 (2.0-31.3)/p=0.001 | Brabin L et al. (2006) | Parents (mother & father) of children (male and female) 11-12 y.o. | Multivariate analysis |  |
| Doesn’t believe that HPV can be extremely harmful | Yes | 4.19 (1.63-10.83) /p=0.03 | Gefenaite et al. (2012) | Parents (mother & father) of female children 13-16 y.o. | Multivariate analysis | 1 / 1 |
| Efficacy of the HPV vaccine is unclear /know too little about the effects of the vaccine | Yes | p<0.001 | Grandahl M et al. (2017) | Parents (mother & father) of female children 11-12 y.o. | Bivariate analysis | 3 / 3 |
|  | Yes | P<0.01 | van Keulen et a. (2013) | Female adolescents & Mothers of female adolescents 13-14 y.o. | Multivariate analysis |  |
|  | Yes | 8.34 (1.41-49.50) /p=0.02 | Gefenaite et al. (2012) | Parents (mother & father) of female children 13-16 y.o. | Multivariate analysis |  |
| HPV vaccine is painful/hurts//side effects /safety concerns | Yes | *0.15 (0.06-0.35)/p<0.001* | Navarro-Illana P et al (2017) | Female adolescents & Parents of female children 12-16 | Multivariate analysis | 12 / 12 |
|  |  | p<0.001 | Grandahl M et al. (2017) | Parents (mother & father) of female children 11-12 y.o. | Bivariate analysis |  |
|  |  | P<0.05 | Balla B et al. (2016  ) | Female adolescents 18-19 y.o. | Bivariate analysis |  |
|  |  | 4.71 (2.13-10.44) /p<0.001 | Gefenaite et al. (2012) | Parents (mother & father) of female children 13-16 y.o. | Multivariate analysis |  |
|  |  | 4.36 (1.26-15.07) | Firenze A et al. (2015) | Female adolescents 15 (13-17) y.o. | Bivariate analysis |  |
|  |  | *0.38 (0.30-0.48) (if vaccine is free)* | Dahlström L et al. (2010) | Parents (mother & father) of children (male and female) 12-15 y.o. | Multivariate analysis |  |
|  |  | *0.26 (0.22-0.30) (if not free)* |  |  |  |  |
|  |  | *0.68 (0.54-0.85)/p=0.001* | Marlow et al. (2009) | Female adolescents 16-19 y.o. | Bivariate / Multivariate analysis |  |
|  |  | p<0.001 | Stretch R et al. (2008) | Parents (mother & father) of female children 12-13 y.o. | Bivariate analysis |  |
|  |  | *0.57 (0.43-0.77)/p<0.001* | Marlow et al. (2007) | Mothers of female children 11 (8-14) y.o. | Multivariate analysis |  |
|  |  | *0.37 (0.28-0.50)/p<0.0001* | Marlow L et al (2007) | Mothers of female children 11 (8-14) y.o. | Multivariate analysis |  |
|  |  | 2.6 (1.2-5.5) Parents | Woodhall SC et al. (2007) | Adolescents (male & female) & their parents 14-15 y.o. | Multivariate analysis |  |
|  |  | *0.59 (0.40-0.88)/p=0.006* | Brabin L et al. (2006) | Parents (mother & father) of children (male and female) 11-12 y.o. | Multivariate analysis |  |
| Number of negative thoughts | Yes | R=-0.51 (p<0.01) | Morison LA et al. (2010) | Parents (mother & father) of female children 11-12 y.o. | Bivariate analysis | 1 / 1 |
| Anticipated regret if daughter has the vaccine | Yes | R=-0.34 (p<0.01) | Morison LA et al. (2010) | Parents (mother & father) of female children 11-12 y.o. | Bivariate analysis | 1 / 1 |
| Not sure that the government would halt vaccinations if there was evidence of serious side effects |  | 10.13 (4.06-25.60) /p<0.001 | Gefenaite et al. (2012) | Parents (mother & father) of female children 13-16 y.o. | Multivariate analysis | 1 / 1 |
| Belief that vaccine will encourage child to have more partners /unprotected sex | Yes | *0.86 (0.74-0.99) (if vaccine is not free)* | Dahlström L et al. (2010) | Parents (mother & father) of children (male and female) 12-15 y.o. | Multivariate analysis | 6 / 6 |
|  |  | *0.78 (0.62-0.97)/p=0.026* | Marlow et al. (2009) | Female adolescents 16-19 y.o. | Bivariate / Multivariate analysis |  |
|  |  | Beta=-0.18; p=0.02 Girls | de Visser E et al. (2008) | Parents (mother & father) of children (male and female) 12-13 y.o. | Multivariate analysis |  |
|  |  | Beta=-0.11; p=0.03 Boys |  |  |  |  |
|  |  | *0.47 (0.36-0.62)/p<0.001* | Marlow L et al (2007) | Mothers of female children 11 (8-14) y.o. | Multivariate analysis |  |
|  |  | 2.1 (1.3-3.3) Parents | Woodhall SC et al. (2007) | Adolescents (male & female) & their parents 14-15 y.o. | Multivariate analysis |  |
|  |  | 1.9 (1.0-3.4) Adolescents |  |  |  |  |
|  |  | *0.10 (0.04-0.26) /p=0.001* | Brabin L et al. (2006) | Parents (mother & father) of children (male and female) 11-12 y.o. | Multivariate analysis |  |
| My daughter does not need to have the HPV vaccination if she is already sexually active | Yes | p<0.01 | van Keulen et a. (2013) | Female adolescents & Mothers of female adolescents 13-14 y.o. | Multivariate analysis | 1 / 1 |
| Concern about sexual health clinics | Yes | p<0.001 | Brabin L et al. (2007) | Parents (mother & father) of children (male and female) 11-12 y.o. | Bivariate analysis | 1 / 1 |
| Communication problems between parents and children | Yes | *0.13 (0.03-0.57)/p=0.009* | Brabin L et al. (2006) | Parents (mother & father) of children (male and female) 11-12 y.o. | Multivariate analysis | 1 / 1 |
| Need for additional information about HPV vaccination | Yes | **2.24 (1.16-4.32)** | **Bianco AS et al. (2014)** | **Parents (mother & father) of male children 10-14 y.o.** | **Multivariate analysis** | 2 / 3 |
|  |  | 13.74 (3.82-49.46) /p<0.001 | Gefenaite et al. (2012) | Parents (mother & father) of female children 13-16 y.o. | Multivariate analysis |  |
| HPV information from friends | Yes | 3.67 (1.63-8.25) | Firenze A et al. (2015) | Female adolescents 15 (13-17) y.o. | Bivariate analysis | 2 / 2 |
|  |  | *0.43 (0.24-0.77) /p=0.003* | Navarro-Illana P et al (2017) | Female adolescents & Parents of female children 12-16 | Multivariate analysis |  |
| HPV information from paediatrician/general practitioner | Yes | *0.40 (0.24-0.68)* | Firenze A et al. (2015) | Female adolescents 15 (13-17) y.o. | Bivariate analysis | 1 / 1 |
| It is difficult to make an appointment for HPV vaccination | Yes | p<0.01 | Grandahl M et al. (2017) | Adolescents (male & female) 16 y.o. | Bivariate analysis | 1 / 1 |
| The HPV vaccination was only introduced because the pharmaceutical industry will make a lot of money from it/government strongly influenced by vaccine producers | Yes | P=0.001 | van Keulen et a. (2013) | Female adolescents & Mothers of female adolescents 13-14 y.o. | Multivariate analysis | 2 / 2 |
|  |  | 3.60 (1.74-7.47) /p=0.001 | Gefenaite et al. (2012) | Parents (mother & father) of female children 13-16 y.o. | Multivariate analysis |  |

In dark grey, factors related to parents’ acceptance of HPV vaccine in males

In light grey, factors related to male adolescents’ acceptance of HPV vaccine

*In italics, factors negatively associated with acceptance of HPV vaccine*

Whenever possible, multivariate data were selected

SM-7. Knowledge of HPV by gender and vaccination status of respondent

| **Item of knowledge of HPV** | **Study** | **Gender of respondent** | | | **Vaccination status of respondent** | | |
| --- | --- | --- | --- | --- | --- | --- | --- |
|  |  | **female adolescents (% Yes responses)** | **male adolescents (% Yes responses)** | **p- value** | **Vaccinated (% Yes responses)** | **Unvaccinated (% Yes responses)** | **p- value** |
| Do you know what HPV is? | Vaidakis D et al. (2017) | 47.9 | 35.5 | p<0.001 |  |  |  |
| Ever heard of HPV | Gottvall M et al. (2009) | 16.40 | 9.60 | p=0.017 |  |  |  |
|  | Pelucchi et al. (2010) | 71.6 | 51.2 | P<0.001 |  |  |  |
|  | Marek E et al. (2011) * | 43.2 | 25.5 | P<0.001 |  |  |  |
|  | Bowyer HL et al. (2013) |  |  |  | 86.8 | 73.8 | p=0.017 |
| Have you heard about HPV? | Patel H et al. (2017) | 35.6 | 8.1 | p<0.001 |  |  |  |
|  | Maier C et al. (2015) | 57.54 | 48.08 | p<0.02 |  |  |  |
|  | Sopracordevole F et al. (2012) | 92.8% | 51.3% | P<0.01 |  |  |  |
|  | Grandahl M et al. (2017) |  |  |  | 38 | 27 | p=0.02 |
| Level of knowledge >4 (on a scale from 1 to 5) | Voidăzan et al. (2016) ** | 35.7 | 24.9 | p<0.001 |  |  |  |
| Heard about vaccine against cervical cancer | Grandahl M et al. (2017) |  |  |  | 99 | 92 | p<0.01 |
| Cervical cancer is related to HPV infection | Sopracordevole F et al. (2013) |  |  |  | 96.1 | 91.8 | p=0.045 |

Figures calculated from Table 1 in Marek E et al. (2011) **Figures calculated from Table 1 in Voidăzan et al. (2016)

**SM-8:** Source of information about HPV and HPV vaccine in adolescents and their parents

|  | **A. Adolescents (T)** | | | | | | | | **P. Parents (T)** | | | | | | | |
| --- | --- | --- | --- | --- | --- | --- | --- | --- | --- | --- | --- | --- | --- | --- | --- | --- |
|  | **Number of studies** | **Number of items** | **Sample size (respondents)** | **N (+; Yes; True)** | **Pooled %** | **Mean %** | **Min %** | **Max. %** | **Number of studies** | **Number of items** | **Sample size (respondents)** | **N (+; Yes; True)** | **Pooled %** | **Mean %** | **Min %** | **Max. %** |
| **Sources of information about HPV** | **5** | **35** | **31692** | **8223** | **25.9** | **24.3** | **0.0** | **94.3** | **5** | **46** | **47981** | **13365** | **27.9** | **29.7** | **1.9** | **92.0** |
| Source of information about HPV: Other |  |  |  |  |  |  |  |  | 2 | 3 | 4071 | 703 | 17.3 | 23.6 | 6.7 | 55.5 |
| Source of information about HPV: Family doctor/medical staff /pharmacist | 4 | 5 | 3581 | 939 | 26.2 | 20.1 | 1.9 | 42.1 | 4 | 9 | 6847 | 1067 | 15.6 | 20.8 | 2.2 | 56.4 |
| Source of information about HPV: Family or friends | 3 | 4 | 339 | 27 | 8.0 | 8.2 | 3.8 | 14.6 | 4 | 8 | 9315 | 1527 | 16.4 | 18.0 | 4.0 | 54.1 |
| Source of information about HPV: Gynaecologist | 1 | 1 | 1662 | 484 | 29.1 | 29.1 | 29.1 | 29.1 | 2 | 3 | 3685 | 1823 | 49.5 | 55.8 | 30.6 | 75.5 |
| Source of information about HPV: The Internet | 2 | 3 | 3393 | 741 | 21.8 | 26.9 | 18.4 | 37.7 | 2 | 3 | 4174 | 1283 | 30.7 | 32.4 | 20.9 | 42.3 |
| Source of information about HPV: Paediatrician | 1 | 1 | 1662 | 221 | 13.3 | 13.3 | 13.3 | 13.3 | 3 | 4 | 4223 | 2778 | 65.8 | 66.6 | 46.9 | 92.0 |
| Source of information about HPV: Press/printed media | 3 | 5 | 5137 | 611 | 11.9 | 16.3 | 4.3 | 42.7 | 4 | 5 | 4696 | 1468 | 31.3 | 32.6 | 22.5 | 46.5 |
| Source of information about HPV: Radio/TV | 3 | 5 | 3568 | 458 | 12.8 | 25.7 | 1.4 | 94.3 | 4 | 6 | 4642 | 1195 | 25.7 | 22.4 | 1.9 | 71.5 |
| Source of information about HPV: School | 5 | 11 | 12350 | 4742 | 38.4 | 34.9 | 0.0 | 61.3 | 3 | 3 | 3072 | 553 | 18.0 | 25.9 | 9.3 | 59.1 |
| Source of information about HPV: Vaccination centre |  |  |  |  |  |  |  |  | 1 | 2 | 3256 | 968 | 29.7 | 29.7 | 24.9 | 34.5 |
| **Sources of information about HPV vaccine** | **3** | **18** | **5515** | **701** | **12.7** | **14.9** | **0.0** | **90.3** | **5** | **45** | **20391** | **5190** | **25.5** | **22.0** | **0.3** | **78.5** |
| Need for additional information about HPV vaccine |  |  |  |  |  |  |  |  | 2 | 5 | 3703 | 976 | 26.4 | 25.4 | 7.7 | 48.0 |
| Source of information about HPV vaccine: Other | 1 | 1 | 350 | 18 | 5.1 | 5.1 | 5.1 | 5.1 | 2 | 2 | 616 | 163 | 26.5 | 25.7 | 11.0 | 40.4 |
| Source of information about HPV vaccine: Family doctor/medical staff | 3 | 3 | 881 | 207 | 23.5 | 18.7 | 4.2 | 32.0 | 5 | 9 | 4743 | 1656 | 34.9 | 32.6 | 8.9 | 78.5 |
| Source of information about HPV vaccine: Family or friends | 2 | 4 | 1122 | 244 | 21.7 | 18.5 | 5.6 | 51.7 | 2 | 5 | 1597 | 274 | 17.2 | 18.3 | 1.0 | 56.5 |
| Source of information about HPV vaccine: Gynaecologist | 1 | 1 | 350 | 21 | 6.0 | 6.0 | 6.0 | 6.0 | 3 | 6 | 2755 | 569 | 20.7 | 18.7 | 3.4 | 32.4 |
| Source of information about HPV vaccine: The Internet | 1 | 1 | 350 | 2 | 0.6 | 0.6 | 0.6 | 0.6 | 2 | 3 | 1013 | 31 | 3.1 | 3.2 | 0.3 | 7.2 |
| Source of information about HPV vaccine: Paediatrician |  |  |  |  |  |  |  |  | 3 | 5 | 2780 | 838 | 30.1 | 25.3 | 1.2 | 58.2 |
| Source of information about HPV vaccine: Press /printed media | 2 | 3 | 1268 | 80 | 6.3 | 6.1 | 3.1 | 12.0 | 2 | 3 | 1013 | 236 | 23.3 | 19.9 | 1.4 | 45.0 |
| Source of information about HPV vaccine: Radio/TV | 2 | 2 | 422 | 88 | 20.9 | 48.4 | 6.6 | 90.3 | 1 | 4 | 1168 | 76 | 6.5 | 6.5 | 0.3 | 18.5 |
| Source of information about HPV vaccine: School | 2 | 2 | 422 | 20 | 4.7 | 2.9 | 0.0 | 5.7 | 1 | 2 | 584 | 47 | 8.0 | 8.0 | 7.5 | 8.6 |
| Source of information about HPV vaccine: Vaccination centre | 1 | 1 | 350 | 21 | 6.0 | 6.0 | 6.0 | 6.0 | 1 | 1 | 419 | 324 | 77.3 | 77.3 | 77.3 | 77.3 |

Number of studies: number of studies reporting these data

Number of items: number of items reporting these data

Sample size (respondents): total number of respondents to any item under this heading or subheading across the included studies

N (+; Yes; True): total number of respondents who responded +, Yes or True to any item under this heading or subheading across the included studies

Pooled %: calculated value using previous columns N /Sample size

Mean %: average of the percentages reported in the studies for the items under this heading or subheading

Min %: minimum of the percentages reported in the studies for the items under this heading or subheading

Max. %: maximum of the percentages reported in the studies for the items under this heading or subheading

**SM-9:** Percentage of HPV Knowledge per country and year of publication (for countries with 2 or more publications)

Mean % of respondents that answered HPV knowledge items correctly

**SM-10:** Percentage of HPV vaccine Knowledge per country and year of publication (for countries with 2 or more publications)

Mean % of respondents that answered HPV vaccine knowledge items correctly

**SM-11:** Intention to vaccinate girls per country and year of publication (for countries with 2 or more publications)

Mean % of respondents that answered “yes” to “intention to vaccinate against HPV (girls)

**SM-12:** Intention to vaccinate boys per country and year of publication (for countries with 2 or more publications))

Mean % of respondents that answered “yes” to “intention to vaccinate against HPV (boys)

**SM-7-** List of items used to evaluate parents and adolescents' knowledge of HPV and knowledge of HPV vaccine in the studies included in the literature review

| **Verbatim of the item (response option)** |  |  |  |
| --- | --- | --- | --- |
|  | **# of items for adolescents** | **# of items por parents** | **Total # of items** |
| **Knowledge about HPV** | **234** | **90** | **324** |
| **General knowledge about HPV** | **30** | **9** | **39** |
| A person could have HPV for many years without knowing it | 1 |  | 1 |
| An HPV infection always leads to cervical cancer | 1 |  | 1 |
| An HPV infection usually disappears on its own | 1 |  | 1 |
| Believe that people are always aware of an ongoing HPV infection |  | 1 | 1 |
| Do you know how common is the infection of sexually active women with HPV? | 2 |  | 2 |
| Do you know what HPV is? | 2 |  | 2 |
| Do you think HPV can be diagnosed? | 2 |  | 2 |
| Do you think HPV infection can be dangerous | 2 | 2 | 4 |
| Having sex at an early age increases the risk of getting HPV | 1 |  | 1 |
| HPV always has visible signs or symptoms | 1 |  | 1 |
| HPV can be cured with antibiotics | 1 |  | 1 |
| HPV infection occurs frequently -HPV infection | 2 |  | 2 |
| HPV infections are rare |  | 1 | 1 |
| HPV is rare | 1 |  | 1 |
| HPV may infect you without symptoms |  | 1 | 1 |
| HPV usually does not need any treatment | 1 |  | 1 |
| Is HPV infection a venereal disease? | 2 |  | 2 |
| Knew about HPV | 2 |  | 2 |
| Know about HPV |  | 1 | 1 |
| Know HPV is an STI |  | 1 | 1 |
| Known STDs | 2 |  | 2 |
| Most HPV infections resolve spontaneously |  | 1 | 1 |
| Most sexually active people will get HPV at some point in their lives | 1 |  | 1 |
| Smoke is a risk factor for HPV | 1 |  | 1 |
| Sometimes HPV infections can last years |  | 1 | 1 |
| There are many types of HPV | 1 |  | 1 |
| What is HPV? | 2 |  | 2 |
| You always notice if you are HPV infected | 1 |  | 1 |
| **Have you heard about HPV?** | **18** | **10** | **28** |
| Ever heard of HPV | 2 | 1 | 3 |
| Had heard about HPV infection | 1 |  | 1 |
| Had heard of HPV |  | 1 | 1 |
| Has heard of infection by HPV |  | 1 | 1 |
| Have you ever heard about human papillomavirus? |  | 1 | 1 |
| Have you ever heard of HPV? | 1 |  | 1 |
| Have you ever heard of human papillomavirus (HPV)? | 2 |  | 2 |
| Have you heard about HPV |  | 1 | 1 |
| Have you heard about HPV infection? |  | 1 | 1 |
| Have you heard about HPV? | 2 |  | 2 |
| Have you heard of HPV? | 4 | 2 | 6 |
| Have you heard of the human papillomavirus (HPV)? | 2 |  | 2 |
| Heard about HPV | 3 | 2 | 5 |
| Previous awareness of HPV | 1 |  | 1 |
| **Knowledge about diseases related to HPV** | **67** | **28** | **95** |
| An HPV infection can cause genital warts | 1 |  | 1 |
| An HPV infection is a risk for cervical cancer | 1 |  | 1 |
| Believes cervical cancer to be common |  | 1 | 1 |
| Believes HPV cause cervical cancer |  | 1 | 1 |
| Believes HPV causes other cancer |  | 1 | 1 |
| Can HPV cause cervical cancer? | 1 |  | 1 |
| Can HPV cause condyloma? | 1 |  | 1 |
| Can HPV cause other forms of cancer? | 1 |  | 1 |
| Cervical cancer is always fatal | 1 |  | 1 |
| Cervical cancer is related to HPV infection | 1 |  | 1 |
| Cervical cancer was caused by infection | 1 |  | 1 |
| Could HPV cause cervical cancer? | 2 |  | 2 |
| Did you know that infections may cause tumours? | 2 |  | 2 |
| Diseases related to HPV (Cervical Cancer) | 1 |  | 1 |
| Diseases related to HPV (Genital Warts) | 1 |  | 1 |
| Diseases related to HPV (Ovarian Cancer) | 1 |  | 1 |
| Do you believe that HPV induces cervical cancer? | 2 |  | 2 |
| Do you know what cervical cancer is? | 2 |  | 2 |
| Ever heard of cervical cancer |  | 1 | 1 |
| For those who heard of HPV: did you know that HPV may cause tumors? | 2 |  | 2 |
| For those who heard of HPV: did you know that HPV may induce cervical cancer? | 2 |  | 2 |
| For those who heard of HPV: did you know that HPV may induce genital warts? | 2 |  | 2 |
| Genital warts are caused by HPV |  | 1 | 1 |
| Have you heard about cervical cancer? | 2 |  | 2 |
| Have you heard of cervical cancer? | 2 |  | 2 |
| HP-related diseases: AIDS | 1 |  | 1 |
| HP-related diseases: Cervical tumours | 1 |  | 1 |
| HP-related diseases: Hepatitis | 1 |  | 1 |
| HP-related diseases: Infectious mononucleosis | 1 |  | 1 |
| HP-related diseases: Warts | 1 |  | 1 |
| HPV as a possible causative agent of CC | 1 |  | 1 |
| HPV can cause cervical cancer | 1 |  | 1 |
| HPV can cause cervical cancer in women | 2 |  | 2 |
| HPV can cause condyloma | 2 |  | 2 |
| HPV can cause genital warts | 1 |  | 1 |
| HPV can cause HIV/AIDs | 1 |  | 1 |
| HPV can cause other types of cancer | 3 |  | 3 |
| HPV can cause: Anal cancer |  | 1 | 1 |
| HPV can cause: Bladder cancer |  | 1 | 1 |
| HPV can cause: Cancer of the oral cavity |  | 1 | 1 |
| HPV can cause: Cervical cancer |  | 1 | 1 |
| HPV can cause: Esophageal cancer |  | 1 | 1 |
| HPV can cause: Genital warts |  | 1 | 1 |
| HPV can cause: Irritable colon |  | 1 | 1 |
| HPV can cause: Recurrent cystitis |  | 1 | 1 |
| HPV infection cause bladder cancer | 1 |  | 1 |
| HPV infection cause cervical cancer | 1 |  | 1 |
| HPV infection cause cystitis | 1 |  | 1 |
| HPV infection cause genital warts | 1 |  | 1 |
| HPV infection cause other diseases | 1 |  | 1 |
| HPV infection may cause cervical cancer |  | 1 | 1 |
| HPV infection may cause genital warts |  | 1 | 1 |
| HPV infection may cause liver cancer |  | 1 | 1 |
| HPV infection may cause ovarian cancer |  | 1 | 1 |
| HPV is associated with AIDS | 1 |  | 1 |
| HPV is associated with genital warts | 1 |  | 1 |
| HPV is associated with hepatitis | 1 |  | 1 |
| HPV is the main cause of cervical cancer |  | 1 | 1 |
| HPV may cause cancer |  | 1 | 1 |
| HPV may cause cervical cancer |  | 1 | 1 |
| In your opinion, what is cervical cancer caused by? | 2 |  | 2 |
| Knew about the causal relation of HPV and cervical cancer |  | 1 | 1 |
| Know about HPV and CC |  | 1 | 1 |
| Knowledge of the incidence of CC in Germany | 1 | 1 | 2 |
| The infection cause CC |  | 1 | 1 |
| The infection causes genital warts |  | 1 | 1 |
| Thinks there is relationship between number of sexual partners and cervical cancer |  | 1 | 1 |
| What is the association between cervical cancer and infection with HPV? | 2 |  | 2 |
| What is the cause of CC? |  | 1 | 1 |
| What is the cause of CC? Related response (STD, viral infection) |  | 1 | 1 |
| Which conditions are caused by HPV? (Cervical cancer) | 1 |  | 1 |
| Which conditions are caused by HPV? (Genital warts) | 2 |  | 2 |
| Which conditions are caused by HPV? (Hepatitis) | 2 |  | 2 |
| Which conditions are caused by HPV? (HIV/AIDS) | 2 |  | 2 |
| Which conditions are caused by HPV? (Infertility) | 2 |  | 2 |
| **Knowledge about diseases related to HPV (boys)** | **9** | **2** | **11** |
| Diseases related to HPV (Penis Cancer) | 1 |  | 1 |
| HP-related diseases: Penile tumours | 1 |  | 1 |
| HPV causes cancer (In men and women) |  | 1 | 1 |
| HPV infection can cause premalignant lesions and carcinosis of cervix and penis -HPV infection | 2 |  | 2 |
| HPV infection may cause penile cancer |  | 1 | 1 |
| HPV is associated with penile cancer | 1 |  | 1 |
| Role of HPV infection among males relating to a male infection | 1 |  | 1 |
| Role of HPV infection among males relating to penis cancer | 1 |  | 1 |
| Which conditions are caused by HPV? (Penile cancer) | 2 |  | 2 |
| **Knowledge about HPV target population** | **1** | **1** | **2** |
| Consider themselves to be at risk of infection | 1 |  | 1 |
| My daughter is in risk to contracting HPV |  | 1 | 1 |
| **Knowledge about HPV target population (boys)** | **5** | **3** | **8** |
| Can men contract HPV? | 1 |  | 1 |
| Knowledge about HPV infection: Risk of HPV infection is related to both sexes |  | 1 | 1 |
| Men can be infected by HPV |  | 1 | 1 |
| Men can be infected with HPV | 1 |  | 1 |
| Men can contract HPV? | 2 |  | 2 |
| Men cannot get HPV | 1 |  | 1 |
| Men could also be infected |  | 1 | 1 |
| **Knowledge about HPV target population (girls)** | **4** | **2** | **6** |
| Can women contract HPV? | 1 |  | 1 |
| Only women can be infected by HPV |  | 1 | 1 |
| Women can be infected by HPV |  | 1 | 1 |
| Women can be infected with HPV | 1 |  | 1 |
| Women can contract HPV | 2 |  | 2 |
| **Knowledge about HPV transmission** | **53** | **15** | **68** |
| Adolescents are at risk of HPV when they start having sex |  | 1 | 1 |
| Aware of the mechanism of transmission of the infection | 1 |  | 1 |
| Believes HPV to be sexually transmitted |  | 1 | 1 |
| Can HPV be transmitted through sexual intercourse? | 1 |  | 1 |
| Do you know how HPV infection is passed on (Sexual intercourse) | 2 | 2 | 4 |
| Having many sexual partners increases the risk of getting HPV | 1 |  | 1 |
| How can HPV infection be transmitted? (By contaminated surfaces) | 2 |  | 2 |
| How can HPV infection be transmitted? (By droplet infection) | 2 |  | 2 |
| How can HPV infection be transmitted? (Sexually, by any forms) | 2 |  | 2 |
| How can HPV infection be transmitted? (Sexually, by vaginal intercourse) | 2 |  | 2 |
| How can HPV infection be transmitted? (Skin to skin) | 2 |  | 2 |
| How is HPV transmitted? (Blood transfusion) | 2 |  | 2 |
| How is HPV transmitted? (Genital skin-to-skin contact) | 2 |  | 2 |
| How is HPV transmitted? (Sexual intercourse) | 2 |  | 2 |
| How is HPV transmitted? (Using public toilets) | 2 |  | 2 |
| HPV can be passed on by genital skin-to-skin contact | 1 |  | 1 |
| HPV can be passed on during sex | 1 |  | 1 |
| HPV can be transmitted via; Deep throat kissing | 1 |  | 1 |
| HPV can be transmitted via; Holding hands | 1 |  | 1 |
| HPV can be transmitted via; Public toilet | 1 |  | 1 |
| HPV can be transmitted via; Sex with a condom | 1 |  | 1 |
| HPV can be transmitted via; Sharing a spoon or cup | 1 |  | 1 |
| HPV can be transmitted via; Skin to skin contact | 1 |  | 1 |
| HPV can be transmitted via; sneezing/coughing | 1 |  | 1 |
| HPV can be transmitted via; Stroking partner at genitals | 1 |  | 1 |
| HPV can be transmitted via; Unprotected anal sex | 1 |  | 1 |
| HPV can be transmitted via; Unprotected oral sex | 1 |  | 1 |
| HPV can be transmitted via; Unprotected vaginal sex |  | 1 | 1 |
| HPV can spread through sexual contact | 1 |  | 1 |
| HPV infection is sexually transmitted | 1 |  | 1 |
| HPV is a sexually transmitted disease |  | 1 | 1 |
| HPV is spread through sexual contact | 2 |  | 2 |
| If you have unprotected sex, you are at high risk of an HPV infection | 1 |  | 1 |
| Intimate kissing | 1 |  | 1 |
| Intime soaps protect against HPV infection | 1 |  | 1 |
| Knew genital warts can be transmitted sexually |  | 1 | 1 |
| Knowledge about HPV infection: HPV is transmitted by sexual intercourse |  | 1 | 1 |
| Lack of hygiene | 1 |  | 1 |
| Number of sexual partners is directly related to the risk of HPV-related lesions | 1 |  | 1 |
| Risk factors for HPV-related diseases: Number of partners | 1 |  | 1 |
| Risk factors for HPV-related diseases: Scarce intimate hygiene | 1 |  | 1 |
| Risk factors for HPV-related diseases: Smoking | 1 |  | 1 |
| Routes of transmission: Blood | 1 |  | 1 |
| Routes of transmission: Sexual | 1 |  | 1 |
| Scarce personal hygiene is a factor risk for HPV-related lesions | 1 |  | 1 |
| Sexual contacts are the only route of HPV transmission | 1 |  | 1 |
| Sexual intercourse | 1 |  | 1 |
| Transmission of HPV infection may be through blood | 1 |  | 1 |
| Ways of transmission: Blood transfusion |  | 1 | 1 |
| Ways of transmission: Contaminated objects |  | 1 | 1 |
| Ways of transmission: Cutaneous transmission (touching warts or intimate touchs) |  | 1 | 1 |
| Ways of transmission: Kiss |  | 1 | 1 |
| Ways of transmission: Public baths or swimming pools |  | 1 | 1 |
| Ways of transmission: Unsterilized needle |  | 1 | 1 |
| Ways of transmission: WC |  | 1 | 1 |
| **Knowledge about HPV transmission (boys)** | **1** |  | **1** |
| Transmitted by boys | 1 |  | 1 |
| **Knowledge about ways to prevent HPV** | **42** | **6** | **48** |
| Attending cervical cancer screening was important for prevention | 1 |  | 1 |
| Condom is necessary for HPV protection | 1 |  | 1 |
| Condoms do not offer 100% protection against HPV |  | 1 | 1 |
| Does condom give full protection from HPV? | 2 |  | 2 |
| How can we prevent venereal diseases? (Condoms) | 2 |  | 2 |
| How can we prevent venereal diseases? (Hygiene) | 2 |  | 2 |
| How can we prevent venereal diseases? (Monogamy) | 2 |  | 2 |
| How can we prevent venereal diseases? (Screening) | 2 |  | 2 |
| How can we prevent venereal diseases? (Vaccine) | 2 |  | 2 |
| How can you reduce your risk of getting HPV? (Antibiotics) | 2 |  | 2 |
| How can you reduce your risk of getting HPV? (Condoms) | 2 |  | 2 |
| How can you reduce your risk of getting HPV? (Good personal hygiene) | 2 |  | 2 |
| How can you reduce your risk of getting HPV? (HPV vaccine) | 2 |  | 2 |
| How can you reduce your risk of getting HPV? (Oral contraceptive pill) | 1 |  | 1 |
| How can you reduce your risk of getting HPV? (Oral contraceptive pill) | 1 |  | 1 |
| How much condoms reduce the risk of cervical cancer? | 2 |  | 2 |
| How much condoms reduce the risk of HPV infection? | 2 |  | 2 |
| HPV infection cannot be prevented | 1 |  | 1 |
| Knowledge about HPV infection Preventive measures for HPV infection: Condom use |  | 1 | 1 |
| Knowledge about HPV infection Preventive measures for HPV infection: Late start of sexual activity |  | 1 | 1 |
| Knowledge about HPV infection Preventive measures for HPV infection: Other contraceptive than condoms |  | 1 | 1 |
| Knowledge about HPV infection Preventive measures for HPV infection: Reducing number of sexual partners |  | 1 | 1 |
| Knowledge about HPV infection Preventive measures for HPV infection: Specific vaccination |  | 1 | 1 |
| Oral contraceptive protects against HPV infection | 1 |  | 1 |
| Prevention of HPV-related diseases correlate: Condoms | 1 |  | 1 |
| Prevention of HPV-related diseases correlate: Intimate soaps | 1 |  | 1 |
| Prevention of HPV-related diseases correlate: Oral contraceptives | 1 |  | 1 |
| Secondary prevention by Pap test at the screening age should be performed for vaccinated girls | 1 |  | 1 |
| Use of condoms avoid the infection | 1 |  | 1 |
| Using condoms reduces the risk of getting HPV | 1 |  | 1 |
| Vaccination is a manner to avoid disease | 2 |  | 2 |
| Ways of preventing HPV diseases: cannot be prevented | 1 |  | 1 |
| Ways of preventing HPV diseases: Good hygiene | 1 |  | 1 |
| Ways of preventing HPV diseases: Hand washing | 1 |  | 1 |
| Ways of preventing HPV diseases: use of condoms | 1 |  | 1 |
| **Knowledge related to Papanicolaou test** | **3** | **5** | **8** |
| Did any of your friends/family members have taken this test? | 1 |  | 1 |
| Do you know what is a Pap test? |  | 1 | 1 |
| Have you ever had a Pap test? |  | 1 | 1 |
| Have you ever heard of the Papanicolaou test? | 1 |  | 1 |
| How often should she have a Pap test? |  | 1 | 1 |
| What is the role of the Pap test? (to prevent CC) |  | 1 | 1 |
| When should a woman have a Pap test? |  | 1 | 1 |
| Which is the main purpose of the Papanicolaou test? | 1 |  | 1 |
| **Social/ familiar experience regarding HPV** | **1** | **8** | **9** |
| Believe in cervical screening programs? |  | 1 | 1 |
| Have you ever talked about HPV with your family members? | 1 |  | 1 |
| HPV associated illness in the family |  | 1 | 1 |
| Know anyone who has had abnormal PAP? |  | 1 | 1 |
| Know anyone who has had cervical biopsy? |  | 1 | 1 |
| Know anyone who has had cervical cancer? |  | 1 | 1 |
| Know anyone who has had conization? |  | 1 | 1 |
| Know anyone who has had genital warts? |  | 1 | 1 |
| Know anyone who has had hysterectomy because of abnormal PAP or cervical cancer? |  | 1 | 1 |
| **Knowledge about HPV vaccine** | **41** | **49** | **90** |
| **General knowledge about HPV vaccine** | **17** | **24** | **41** |
| At what age is the first dose of HPV vaccine recommended |  | 1 | 1 |
| Believes HPV vaccine fully protects vs. cervical cancer |  | 1 | 1 |
| Clinical trials show vaccine prevents persistent infection |  | 1 | 1 |
| Do you think girls will be completely protected against cervical cancer after HPV vaccination? |  | 1 | 1 |
| How many doses does vaccination require? |  | 1 | 1 |
| HPV vaccination protects against all HPV types | 1 |  | 1 |
| HPV vaccination protects against all STIs | 1 |  | 1 |
| HPV vaccine completely protects against cervical cancer | 1 |  | 1 |
| HPV vaccine in Italy is available only for girls? |  | 1 | 1 |
| HPV vaccines require three doses? | 1 |  | 1 |
| Someone who has had an HPV vaccine cannot develop cervical cancer | 1 |  | 1 |
| The HPV vaccine offers protection against most cervical cancers | 1 |  | 1 |
| HPV vaccine prevents: Anal cancer |  | 1 | 1 |
| HPV vaccine prevents: Cervical cancer |  | 1 | 1 |
| HPV vaccine prevents: Condyloma acuminate |  | 1 | 1 |
| HPV vaccine prevents: Oral cancer |  | 1 | 1 |
| HPV vaccine protect against cervical cancer | 1 |  | 1 |
| HPV vaccine protect against genital warts | 1 |  | 1 |
| HPV vaccine protect against HIV/AIDS | 1 |  | 1 |
| HPV vaccine protect against viral hepatitis | 1 |  | 1 |
| HPV vaccines protect against all HPV types |  | 1 | 1 |
| Is it better to vaccinate as many people as possible in order to protect unvaccinated ones? |  | 1 | 1 |
| The aim of HPV vaccination: (to prevent cervical cancer) | 2 | 2 | 4 |
| The aim of HPV vaccination: (to prevent STD) |  | 2 | 2 |
| The vaccine does not protect if you already have HPV 16/18 |  | 1 | 1 |
| The vaccine is effective | 1 |  | 1 |
| The vaccine is safe | 1 |  | 1 |
| The vaccine prevents changes to cervical cells |  | 1 | 1 |
| The vaccine protects against all sexually transmitted diseases and allows to have safe sex | 1 |  | 1 |
| The vaccine protects against cervical cancer |  | 1 | 1 |
| The vaccine protects against genital warts |  | 1 | 1 |
| The vaccine protects against liver cancer |  | 1 | 1 |
| The vaccine protects against ovarian cancer |  | 1 | 1 |
| The vaccine protects against sexually transmitted diseases, but it is necessary to adopt methods of prevention during sexual intercourse | 1 |  | 1 |
| The vaccine provides protection for at least 5 years |  | 1 | 1 |
| Vaccine prevents HPV 16 and 18 |  | 1 | 1 |
| Vaccines are available for CC | 1 |  | 1 |
| **General knowledge about HPV vaccine (boys)** |  | **1** | **1** |
| HPV vaccine prevents: Prostate cancer |  | 1 | 1 |
| **Have you heard about HPV vaccine?** | **14** | **3** | **17** |
| Are you informed about HPV vaccine? | 2 |  | 2 |
| Ever heard of any vaccine against HPV | 2 |  | 2 |
| Had heard of HPV vaccine |  | 1 | 1 |
| Had heard of the vaccine | 1 |  | 1 |
| Have you ever heard of HPV vaccination? |  | 1 | 1 |
| Have you ever heard of HPV vaccine? | 2 |  | 2 |
| Have you heard of the HPV vaccine? | 2 | 1 | 3 |
| Heard about HPV vaccine | 2 |  | 2 |
| Heard about vaccine against cervical cancer | 1 |  | 1 |
| Heard of the vaccination against cervical cancer | 1 |  | 1 |
| Is there a vaccine against HPV? | 1 |  | 1 |
| **Knowledge about HPV vaccine target population** |  | **9** | **9** |
| At what age should someone undergo HPV vaccination? (11-13 years) |  | 1 | 1 |
| At what age should someone undergo HPV vaccination? (14-17 years) |  | 1 | 1 |
| At what age should someone undergo HPV vaccination? (18-21 years) |  | 1 | 1 |
| At what age should someone undergo HPV vaccination? (22-26 years) |  | 1 | 1 |
| At what age should someone undergo HPV vaccination? (All ages) |  | 1 | 1 |
| At which age is vaccination recommended? 14-23 |  | 1 | 1 |
| At which age is vaccination recommended? Answer close to recommendation: (14–23 +/− 4 years) |  | 1 | 1 |
| In regard to sexual activity, when is HPV vaccination required? (at any moment) |  | 1 | 1 |
| Who should be vaccinated? (Females) |  | 1 | 1 |
| **Knowledge about HPV vaccine target population (boys)** |  | **2** | **2** |
| Who should be vaccinated? (Males) |  | 1 | 1 |
| Who should be vaccinated? Males and females |  | 1 | 1 |
| **Knowledge on prevention of HPV after vaccination** | **7** | **2** | **9** |
| Condoms are not needed anymore once vaccinated | 1 |  | 1 |
| How should a woman behave towards Pap test after HPV vaccination? (Pap test is still necessary) |  | 1 | 1 |
| HPV vaccine knowledge items. Girls who have had the HPV vaccine do not need cervical screening (a smear or Pap test) when they are older | 1 |  | 1 |
| If vaccinated I will need cervical cancer screening | 1 |  | 1 |
| If vaccinated I will never get HPV infection | 1 |  | 1 |
| If vaccinated I will not need condom any longer in sexual intercourses | 1 |  | 1 |
| Need to participate in the CC screening program after vaccination | 1 |  | 1 |
| Secondary prevention for vaccinated girls: a condom should be used for occasional sexual intercourse | 1 |  | 1 |
| Women will still need to go for cervical smear |  | 1 | 1 |
| **Vaccination before sexual debut** | **3** | **7** | **10** |
| At what age is the first dose of HPV vaccine recommended: Before the start of sexual activity |  | 1 | 1 |
| HPV vaccine knowledge items. The HPV vaccine is most effective if given to people who have never had sex | 1 |  | 1 |
| HPV vaccines work well if given before sexual debut |  | 1 | 1 |
| Know the appropriate age for starting the immunization |  | 1 | 1 |
| Vaccination be given: before sexual activity begins | 2 | 2 | 4 |
| Who should be vaccinated? (Answer nearly correct: (Young girls before or within a year of first intercourse) |  | 1 | 1 |
| Who should be vaccinated? (Young girls before or within a year of first intercourse) |  | 1 | 1 |
| **General total** | **275** | **139** | **414** |

**SM-8-** List of items used to evaluate parents and adolescents' HPV vaccine acceptability in the studies included in the literature review.

| **Verbatim of the item (response option)** | **# of items for adolescents** | **# of items por parents** | **Total # of items** |
| --- | --- | --- | --- |
| **Acceptability of HPV vaccination** | **141** | **286** | **427** |
| **Agree to pay for the vaccine** |  | **1** | **1** |
| Acceptance of HPV vaccination: Willing to vaccinate even the vaccine is not free |  | 1 | 1 |
| **Agree to pay for the vaccine (boys)** | **3** | **1** | **4** |
| Could afford approximately 330 EUR | 1 |  | 1 |
| Intention to vaccinate 12-15 years old boy: Even if we have to cover the cost of vaccine on ourselves |  | 1 | 1 |
| Thought 330 EUR to be realistic compared to domestic earnings | 1 |  | 1 |
| Would even pay for the vaccination | 1 |  | 1 |
| **Agree to pay for the vaccine (girls)** | **3** | **2** | **5** |
| Could afford approximately 330 EUR | 1 |  | 1 |
| If your daughter was out of the age range for free HPV immunization, would you pay to vaccinate her? |  | 1 | 1 |
| Thought 330 EUR to be realistic compared to domestic earnings | 1 |  | 1 |
| Willing to pay 100 euros to vaccinate older daughter too |  | 1 | 1 |
| Would even pay for the vaccination | 1 |  | 1 |
| **Agree to vaccinate at certain age** |  | **12** | **12** |
| 10 and 14 years would be an appropriate age for vaccination |  | 1 | 1 |
| At what age (in years) should vaccination start? >= 18 |  | 1 | 1 |
| At what age (in years) should vaccination start? 12-14 |  | 1 | 1 |
| At what age (in years) should vaccination start? 15-17 |  | 1 | 1 |
| At what age (in years) should vaccination start? 9-11 |  | 1 | 1 |
| At what age should vaccination start? 11-12 |  | 1 | 1 |
| At what age should vaccination start? 13-14 |  | 1 | 1 |
| At what age should vaccination start? 15-16 |  | 1 | 1 |
| At what age should vaccination start? 17-18 |  | 1 | 1 |
| At what age should vaccination start? Earlier (9-11) |  | 1 | 1 |
| Early vaccination (12 years or younger) |  | 1 | 1 |
| Later vaccination (>12 years) |  | 1 | 1 |
| **Agree to vaccinate if it is free** |  | **1** | **1** |
| Acceptance of HPV vaccination: Willing to vaccinate only if the vaccine is free |  | 1 | 1 |
| **Agree to vaccinate if it is free (boys)** | **1** | **3** | **4** |
| Intention to vaccinate 12-15 years old boy: Yes, if the vaccine is included in the publicly financed NIP |  | 1 | 1 |
| What are the main reasons for wanting your son(s) to receive HPV vaccination? If HPV vaccination was included in the CIP, I would vaccinate without questioning |  | 1 | 1 |
| Willing to vaccinate their son if the vaccine is free |  | 1 | 1 |
| Would request the HPV vaccine if it was free | 1 |  | 1 |
| **Agree to vaccinate if it is free (girls)** | **1** | **1** | **2** |
| Reason for not accept HPV vaccination: We did not Know that HPV vaccine was free-of-charge |  | 1 | 1 |
| Would request the HPV vaccine if it was free | 1 |  | 1 |
| **Barriers to accept and reasons for refusing HPV vaccination** | **16** | **20** | **36** |
| Believes that vaccination for STDs increases the likelihood of early sexual debut (YES) - children - | 1 |  | 1 |
| Believes that vaccination for STDs increases the likelihood of early sexual debut (YES) - parents - |  | 1 | 1 |
| Child missed school at the day of vaccination |  | 1 | 1 |
| Child too young for the vaccine |  | 1 | 1 |
| Delayed a previous vaccination |  | 1 | 1 |
| Did not favour vaccinating her/himself | 1 |  | 1 |
| Did not favour vaccinating: afraid of injection | 1 |  | 1 |
| Did not favour vaccinating: afraid of pain | 1 |  | 1 |
| Did not favour vaccinating: afraid of side effects | 1 |  | 1 |
| Did not favour vaccinating: Other (e.g. expensive) | 1 |  | 1 |
| Discouraging information about the vaccine from internet |  | 1 | 1 |
| GP/Paediatrician does not believe it is necessary |  | 1 | 1 |
| Had a bad reaction to a previous vaccination |  | 1 | 1 |
| It is difficult to make an appointment for HPV vaccination | 1 |  | 1 |
| Not being adequately informed |  | 1 | 1 |
| Other reasons/Vaccine too new |  | 1 | 1 |
| Reason with the highest impact on your decision to get vaccinated: Afraid of the needle | 1 |  | 1 |
| Reason with the highest impact on your decision to get vaccinated: Against vaccination | 1 |  | 1 |
| Reason with the highest impact on your decision to get vaccinated: Don’t feel exposed for disease | 1 |  | 1 |
| Reason with the highest impact on your decision to get vaccinated: Not recommended by the physician | 1 |  | 1 |
| Reason with the highest impact on your decision to get vaccinated: The easy treatment of the disease | 1 |  | 1 |
| Reason with the highest impact on your decision to get vaccinated: The price of the vaccine | 1 |  | 1 |
| Reason with the highest impact on your decision to get vaccinated: The side-effects of vaccination | 1 |  | 1 |
| Reasons why they did not want to have their child vaccinated: did not know details or were otherwise poorly informed about the vaccine |  | 1 | 1 |
| Reasons why they did not want to have their child vaccinated: fear side effects |  | 1 | 1 |
| Reasons why they did not want to have their child vaccinated: were in doubt regarding the safety or efficacy of vaccine |  | 1 | 1 |
| Refused a previous vaccination |  | 1 | 1 |
| Regret having given a previous vaccination |  | 1 | 1 |
| STD vaccine leads to early initiation of sexual activity | 1 | 1 | 2 |
| The HPV vaccine has severe side effects | 1 |  | 1 |
| The vaccine is just a publicity ploy by pharmaceutical company |  | 1 | 1 |
| Vaccine not effective |  | 1 | 1 |
| Vaccine unnecessary due to low disease risk |  | 1 | 1 |
| Wait for vaccine which covers more HPV types |  | 1 | 1 |
| Worried child will have more partners/unprotected sex |  | 1 | 1 |
| **Barriers to accept and reasons for refusing HPV vaccination (boys)** | **14** | **28** | **42** |
| Find it embarrassing to discuss HPV vaccination with parents | 1 |  | 1 |
| I don’t know enough about HPV related diseases |  | 2 | 2 |
| I don’t know enough about HPV vaccination |  | 2 | 2 |
| I fear side effects |  | 2 | 2 |
| If you have not been vaccinated for HPV which was the main reason? I am not sexually active | 1 |  | 1 |
| If you have not been vaccinated for HPV which was the main reason? I have not heard about it | 1 |  | 1 |
| If you have not been vaccinated for HPV which was the main reason? I was afraid of the pain | 1 |  | 1 |
| If you have not been vaccinated for HPV which was the main reason? It is expensive | 1 |  | 1 |
| If you have not been vaccinated for HPV which was the main reason? My parents thought it was unsafe | 1 |  | 1 |
| It goes against my cultural/ religious beliefs |  | 1 | 1 |
| Lack of recommendation from HCP |  | 1 | 1 |
| Lack of recommendation from HPC |  | 1 | 1 |
| My son is too young |  | 1 | 1 |
| Need more information before considering a HPV vaccination | 1 |  | 1 |
| Obstacles to vaccination: Afraid of needles | 1 |  | 1 |
| Obstacles to vaccination: Do not believe they will be infected | 1 |  | 1 |
| Obstacles to vaccination: High cost of the vaccine | 1 |  | 1 |
| Obstacles to vaccination: Other | 1 |  | 1 |
| Obstacles to vaccination: Pain from the injection | 1 |  | 1 |
| Obstacles to vaccination: Parents would not allow | 1 |  | 1 |
| Reasons for not having your son vaccinated against HPV: Contrary to many vaccinations |  | 1 | 1 |
| Reasons for not having your son vaccinated against HPV: Doubtful efficacy |  | 1 | 1 |
| Reasons for not having your son vaccinated against HPV: Doubtful utility |  | 1 | 1 |
| Reasons for not having your son vaccinated against HPV: Fear of side effects |  | 1 | 1 |
| Reasons for not having your son vaccinated against HPV: He is too young |  | 1 | 1 |
| Reasons for not having your son vaccinated against HPV: It would be better that children make their own decisions |  | 1 | 1 |
| Reasons for not having your son vaccinated against HPV: Vaccination is not suitable for males/vaccination for girls is enough |  | 1 | 1 |
| Reasons for not having your son vaccinated against HPV: Vaccination will encourage unprotected sexual intercourse/the condom is preferable |  | 1 | 1 |
| Reasons for not informing their children about HPV infection: It will excite curiosity for sex |  | 1 | 1 |
| Reasons for not informing their children about HPV infection: This is not an interesting subject for children |  | 1 | 1 |
| Should you receive more information will you be willing to get the HPV vaccine? Yes | 1 |  | 1 |
| What are the main reasons you do not want/are uncertain about your son(s) receiving HPV vaccination? Better for him/them to use condoms as protection against HPV infections |  | 1 | 1 |
| What are the main reasons you do not want/are uncertain about your son(s) receiving HPV vaccination? Difficulties talking about such (sexual) matters |  | 1 | 1 |
| What are the main reasons you do not want/are uncertain about your son(s) receiving HPV vaccination? Fear of side effects (the vaccination is new/has not been in use long enough) |  | 1 | 1 |
| What are the main reasons you do not want/are uncertain about your son(s) receiving HPV vaccination? He is/they are too young – not yet relevant |  | 1 | 1 |
| What are the main reasons you do not want/are uncertain about your son(s) receiving HPV vaccination? I am against (too many) vaccinations |  | 1 | 1 |
| What are the main reasons you do not want/are uncertain about your son(s) receiving HPV vaccination? Lack of recommendations |  | 1 | 1 |
| What are the main reasons you do not want/are uncertain about your son(s) receiving HPV vaccination? Sufficient that girls are HPV vaccinated |  | 1 | 1 |
| What are the main reasons you do not want/are uncertain about your son(s) receiving HPV vaccination? The vaccination is unsuitable for boys |  | 1 | 1 |
| **Barriers to accept and reasons for refusing HPV vaccination (girls)** | **38** | **44** | **82** |
| Afraid of needles | 1 |  | 1 |
| Although it prevented cancer, it had important problems | 1 |  | 1 |
| Believes HPV vaccine will increase promiscuity |  | 1 | 1 |
| Cervical cancer is not something I’m worried about right now for my daughter -behavioral determinants and declining HPV |  | 1 | 1 |
| Find it embarrassing to discuss HPV vaccination with parents | 1 |  | 1 |
| HPV is not that serious to get vaccinated for -behavioral determinants and declining HPV vaccination |  | 1 | 1 |
| HPV vaccination would make girls more likely to have sex (YES) |  | 1 | 1 |
| I don’t believe HPV can be extremely harmful -behavioral determinants and declining HPV |  | 1 | 1 |
| I don’t believe HPV can cause cervical cancer -behavioral determinants and declining HPV |  | 1 | 1 |
| I don’t believe that cervical cancer is a serious disease -behavioral determinants and declining HPV |  | 1 | 1 |
| I have no trust that the government would stop the vaccinations if there was evidence of serious side effects -determinants of declining the HPV vaccine |  | 1 | 1 |
| I need more information about vaccine | 1 |  | 1 |
| I pay attention not to get infected with HPV -Reasons for refusal of HPV vaccination of unvaccinated girls | 1 |  | 1 |
| I prefer to wait |  | 1 | 1 |
| I think that this vaccination is useless |  | 1 | 1 |
| If you have not been vaccinated for HPV which was the main reason? I am not sexually active | 1 |  | 1 |
| If you have not been vaccinated for HPV which was the main reason? I have not heard about it | 1 |  | 1 |
| If you have not been vaccinated for HPV which was the main reason? I was afraid of the pain | 1 |  | 1 |
| If you have not been vaccinated for HPV which was the main reason? It is expensive | 1 |  | 1 |
| If you have not been vaccinated for HPV which was the main reason? My parents thought it was unsafe | 1 |  | 1 |
| It’s not likely that my daughter gets cervical cancer in the future -behavioral determinants and declining HPV |  | 1 | 1 |
| It’s not likely that my daughter gets infected with HPV some day -behavioral determinants and declining HPV) |  | 1 | 1 |
| It’s not possible that my daughter gets cervical cancer in the future -behavioral determinants and declining HPV |  | 1 | 1 |
| It’s not possible that my daughter gets infected with HPV some day -behavioral determinants and declining HPV |  | 1 | 1 |
| Need more information before considering a HPV vaccination | 1 |  | 1 |
| No interest in HPV vaccinations -Reasons for refusal of HPV vaccination of unvaccinated girls | 1 |  | 1 |
| Obstacles to vaccination: Afraid of needles | 1 |  | 1 |
| Obstacles to vaccination: Do not believe they will be infected | 1 |  | 1 |
| Obstacles to vaccination: High cost of the vaccine | 1 |  | 1 |
| Obstacles to vaccination: Other | 1 |  | 1 |
| Obstacles to vaccination: Pain from the injection | 1 |  | 1 |
| Obstacles to vaccination: Parents would not allow | 1 |  | 1 |
| Parents had advised against HPV vaccination -Reasons for non-vaccination of HPV vaccination of unvaccinated girls- | 1 |  | 1 |
| Physician had advised against HPV vaccination -Reasons for non-vaccination of HPV vaccination of unvaccinated girls | 1 |  | 1 |
| Potential vaccine side effects |  | 1 | 1 |
| Reason for not accept HPV vaccination: Regular pap-test can prevent cervical cancer |  | 1 | 1 |
| Reason for not accept HPV vaccination: Alternative medical approach, not including vaccinations |  | 1 | 1 |
| Reason for not accept HPV vaccination: Contraindications to vaccination |  | 1 | 1 |
| Reason for not accept HPV vaccination: Discordance of information received by HCWs on HPV vaccination |  | 1 | 1 |
| Reason for not accept HPV vaccination: Familiars/friend's advice against HPV vaccination |  | 1 | 1 |
| Reason for not accept HPV vaccination: Family doctor's advice against HPV vaccination |  | 1 | 1 |
| Reason for not accept HPV vaccination: Fear of adverse events |  | 1 | 1 |
| Reason for not accept HPV vaccination: Fear of injection |  | 1 | 1 |
| Reason for not accept HPV vaccination: HPV infection is not severe |  | 1 | 1 |
| Reason for not accept HPV vaccination: HPV vaccination not compulsory |  | 1 | 1 |
| Reason for not accept HPV vaccination: HPV vaccination not useful |  | 1 | 1 |
| Reason for not accept HPV vaccination: HPV vaccination promotes risk sexual behaviours |  | 1 | 1 |
| Reason for not accept HPV vaccination: It is difficult to get a date for vaccination |  | 1 | 1 |
| Reason for not accept HPV vaccination: Lack of trust in a new vaccine |  | 1 | 1 |
| Reason for not accept HPV vaccination: Other health care worker's advice against HPV vaccination |  | 1 | 1 |
| Reason for not accept HPV vaccination: Our daughter is young and not sexually active |  | 1 | 1 |
| Reason for not accept HPV vaccination: Scarce promotion of HPV vaccination |  | 1 | 1 |
| Reason for not accept HPV vaccination: Scare information on HPV vaccination |  | 1 | 1 |
| Reason for not accept HPV vaccination: Vaccination service is difficult to reach |  | 1 | 1 |
| Reason for not accept HPV vaccination: We were not able to respect the date |  | 1 | 1 |
| Reason not to vaccinate: I don't want this vaccine because I don't think it is necessary |  | 1 | 1 |
| Reason not to vaccinate: I have not been given enough information on this vaccine |  | 1 | 1 |
| Reason not to vaccinate: I'd rather wait |  | 1 | 1 |
| Reason not to vaccinate: I'm worried about adverse events |  | 1 | 1 |
| Reason not to vaccinate: My doctor said it was not necessary |  | 1 | 1 |
| Reason not to vaccinate: She has received some doses of the vaccine and the last one is pending |  | 1 | 1 |
| Reasons for not vaccinate or undervaccinate: Administrative reasons (Yes) | 1 |  | 1 |
| Reasons for not vaccinate or undervaccinate: Health reasons | 1 |  | 1 |
| Reasons for not vaccinate or undervaccinate: Lack of parental consent (with explanation) | 1 |  | 1 |
| Reasons for not vaccinate or undervaccinate: Lack of parental consent (without explanation) | 1 |  | 1 |
| Reasons for not vaccinate or undervaccinate: Need for more information | 1 |  | 1 |
| Reasons for not vaccinate or undervaccinate: Other reasons | 1 |  | 1 |
| Reasons for not vaccinate or undervaccinate: Procedural issues | 1 |  | 1 |
| Reasons for not vaccinate or undervaccinate: Safety concerns | 1 |  | 1 |
| Reasons for not vaccinate or undervaccinate: The vaccine isn’t needed | 1 |  | 1 |
| Religious conviction -determinants of declining the HPV vaccine |  | 1 | 1 |
| Risk of unprotected sex (if vaccine is taken) |  | 1 | 1 |
| Should you receive more information will you be willing to get the HPV vaccine? | 1 |  | 1 |
| The most common topic was that the vaccine hurts | 1 |  | 1 |
| The most common topic was that the vaccine it was a source of diseases | 1 |  | 1 |
| The most common topic was that the vaccine was very reactogenic | 1 |  | 1 |
| They would definitely because they were not at risk of infection | 1 |  | 1 |
| They would definitely not have the vaccine because of the adverse events | 1 |  | 1 |
| Too time-consuming-Reasons for refusal of HPV vaccination of unvaccinated girls | 1 |  | 1 |
| Vaccine might be dangerous | 1 |  | 1 |
| We know way too little about the effects of the vaccine -determinants of declining the HPV vaccine |  | 1 | 1 |
| Worried about side effects from HPV vaccination | 1 |  | 1 |
| **Concerns with HPV vaccine** |  | **7** | **7** |
| Which concerns do you have about the HPV vaccine? if the vaccine gives full protection |  | 1 | 1 |
| Which concerns do you have about the HPV vaccine? if the vaccine has any adverse effects |  | 1 | 1 |
| Which concerns do you have about the HPV vaccine? if vaccination has to be repeated |  | 1 | 1 |
| Which of those concerns would make you abstain from vaccinating your child? if the vaccination has to be repeated |  | 1 | 1 |
| Which of those concerns would make you abstain from vaccinating your child? If the vaccine does not fully protect |  | 1 | 1 |
| Which of those concerns would make you abstain from vaccinating your child? if the vaccine has adverse effects |  | 1 | 1 |
| Which of those concerns would make you abstain from vaccinating your child? Other issues |  | 1 | 1 |
| **Concerns with HPV vaccine (girls)** | **3** | **20** | **23** |
| Concerns about side-effects or bad experiences with vaccines in general -Reasons for non-vaccination of HPV vaccination of unvaccinated girls | 1 |  | 1 |
| Concerns specifically about HPV vaccine safety -Reasons for refusal of HPV vaccination of unvaccinated girls | 1 |  | 1 |
| Concerns that HPV vaccine is new and further research is needed on safety and efficacy -Reasons for refusal of HPV vaccination of unvaccinated girls | 1 |  | 1 |
| Doctors do not take parents seriously regarding the side effects of vaccinations - concerns, organisational determinants and declining the HPV vaccination |  | 1 | 1 |
| Girls who had the HPV vaccination would be more likely to have unprotected sex - concerns, organisational determinants and declining the HPV vaccination |  | 1 | 1 |
| Having the HPV vaccination might make girls more likely to have sex - concerns, organisational determinants and declining the HPV vaccination |  | 1 | 1 |
| I feel I didn’t get enough information to make a good decision - concerns, organisational determinants and declining the HPV vaccination |  | 1 | 1 |
| I think it’s good that the HPV vaccine exists, but not at this age - concerns, organisational determinants and declining the HPV vaccination |  | 1 | 1 |
| I think the information about the vaccine provided by the government was not very clear - concerns, organisational determinants and declining the HPV vaccination |  | 1 | 1 |
| I think the information about the vaccine provided by the government was very limited/biased - concerns, organisational determinants and declining the HPV vaccination |  | 1 | 1 |
| I won’t do everything to prevent my daughter getting cervical cancer - concerns, organisational determinants and declining the HPV vaccination |  | 1 | 1 |
| I would get my daughter vaccinated if the vaccine wasn’t only for girls but also for boys - concerns, organisational determinants and declining the HPV vaccination |  | 1 | 1 |
| I would have had more information to make a good decision - concerns, organisational determinants and declining the HPV vaccination |  | 1 | 1 |
| I would strongly disapprove if my daughter would be sexually active at this age - concerns, organisational determinants and declining the HPV vaccination |  | 1 | 1 |
| I’m very worried about the side effects of the HPV vaccination - concerns, organisational determinants and declining the HPV vaccination |  | 1 | 1 |
| It wasn’t very clear when my daughter could get the HPV vaccine - concerns, organisational determinants and declining the HPV vaccination |  | 1 | 1 |
| Other girls might be vaccinated, but my daughter won’t - concerns, organisational determinants and declining the HPV vaccination |  | 1 | 1 |
| Reason for not accept HPV vaccination: Religious concerns |  | 1 | 1 |
| The government is strongly influenced by the vaccine producers - concerns, organisational determinants and declining the HPV vaccination |  | 1 | 1 |
| There are already too many vaccines in the Dutch vaccination program - concerns, organisational determinants and declining the HPV vaccination |  | 1 | 1 |
| There weren’t enough locations to get the vaccination - concerns, organisational determinants and declining the HPV vaccination |  | 1 | 1 |
| We don’t know a lot about the side effects of the vaccine - concerns, organisational determinants and declining the HPV vaccination |  | 1 | 1 |
| We know way too little about the effects of the vaccine - concerns, organisational determinants and declining the HPV vaccination |  | 1 | 1 |
| **Drivers for accepting HPV vaccination (attitudes & beliefs)** | **17** | **8** | **25** |
| Avoiding the risk of becoming infertile | 1 |  | 1 |
| Believes vaccines are safe |  | 1 | 1 |
| Cervical cancer can be life-threatening | 1 |  | 1 |
| Do you agree that it is better to vaccinate a child before he/she becomes sexually active? |  | 1 | 1 |
| Do you believe your child will be fully protected against condyloma after HPV vaccination? |  | 1 | 1 |
| Do you think HPV infection might concern your children (for parents)/you (for students)? |  | 1 | 1 |
| Do you think that HPV vaccination is necessary? |  | 1 | 1 |
| HPV can lead to a serious disease | 1 |  | 1 |
| HPV vaccination protects against cervical cancer | 1 |  | 1 |
| HPV vaccination protects against condyloma | 1 |  | 1 |
| If HPV vaccine was effective for you, would you get vaccinated |  | 1 | 1 |
| Perceived susceptibility | 1 | 1 | 2 |
| Protection against an incurable disease | 1 |  | 1 |
| Protection against cancer | 1 |  | 1 |
| Reason with the highest impact on your decision to get vaccinated: Being ill disturbs me or makes me lose time | 1 |  | 1 |
| Reason with the highest impact on your decision to get vaccinated: Being ill is unpleasant | 1 |  | 1 |
| Reason with the highest impact on your decision to get vaccinated: The fact that the vaccination is mandatory | 1 |  | 1 |
| Reason with the highest impact on your decision to get vaccinated: The protection of people around you | 1 |  | 1 |
| Reason with the highest impact on your decision to get vaccinated: The recommendation by a physician | 1 |  | 1 |
| Reason with the highest impact on your decision to get vaccinated: The recommendation by your friends or relatives | 1 |  | 1 |
| Reason with the highest impact on your decision to get vaccinated: The recommendation by your parents | 1 |  | 1 |
| Reason with the highest impact on your decision to get vaccinated: The seriousness of the disease | 1 |  | 1 |
| Reason with the highest impact on your decision to get vaccinated: To avoid costs related to the treatment | 1 |  | 1 |
| Should there be a cancer history in your family, would you opt for vaccination? |  | 1 | 1 |
| **Drivers for accepting HPV vaccination (attitudes & beliefs) (boys)** | **2** | **29** | **31** |
| Both sexes are equally responsible for preventing STD |  | 1 | 1 |
| Both sexes should have equal rights to vaccination |  | 1 | 1 |
| Do you think that boys should be vaccinated against HPV to reduce the overall transmission of HPV in the population? |  | 1 | 1 |
| Fear of HPV related diseases if not vaccinated |  | 1 | 1 |
| If HPV vaccination is recommended by a HC |  | 1 | 1 |
| My son is also at risk from HPV infection |  | 1 | 1 |
| Perception of vaccine efficacy: 95% | 1 |  | 1 |
| Positive attitudes to inform their children about HPV infection |  | 1 | 1 |
| Positive attitudes toward HPV vaccination for their sons |  | 1 | 1 |
| Prevention of any disease related to HPV infection (pre-cancerous lesions, cancer or benign lesions such as genital warts) |  | 1 | 1 |
| Reasons for vaccinating your son against HPV: Experiences of cancer in family |  | 1 | 1 |
| Reasons for vaccinating your son against HPV: Protecting females from cervical cancer |  | 1 | 1 |
| Reasons for vaccinating your son against HPV: Protecting males from cancers |  | 1 | 1 |
| Reasons for vaccinating your son against HPV: Protecting males from genital warts |  | 1 | 1 |
| Reasons for vaccinating your son against HPV: Recommended by paediatrician/family physician |  | 1 | 1 |
| Reasons for vaccinating your son against HPV: Reducing infection in both sexes |  | 1 | 1 |
| Reasons for vaccinating your son against HPV: The vaccine is required/provided by the Ministry of Health |  | 1 | 1 |
| Reasons for vaccinating your son against HPV: Willingness to receive all effective vaccines |  | 1 | 1 |
| To protect my son against STD |  | 1 | 1 |
| To protect my son’s future partners |  | 2 | 2 |
| Vaccination should be offered to both, boys and girls |  | 1 | 1 |
| What are the main reasons for wanting your son(s) to receive HPV vaccination? (If) HPV vaccination was recommended by a health care professional or the National Board of Health |  | 1 | 1 |
| What are the main reasons for wanting your son(s) to receive HPV vaccination? High risk of HPV infection in my son |  | 1 | 1 |
| What are the main reasons for wanting your son(s) to receive HPV vaccination? I welcome any protection against cancer |  | 1 | 1 |
| What are the main reasons for wanting your son(s) to receive HPV vaccination? If HPV vaccination was included in the CIP, I would not fear side effects |  | 1 | 1 |
| What are the main reasons for wanting your son(s) to receive HPV vaccination? Personal experience with cancer among my close family/relations |  | 1 | 1 |
| What are the main reasons for wanting your son(s) to receive HPV vaccination? Personal experience with GWs among my close family/relations |  | 1 | 1 |
| What are the main reasons for wanting your son(s) to receive HPV vaccination? To protect my son against cancer |  | 1 | 1 |
| What are the main reasons for wanting your son(s) to receive HPV vaccination? To protect my son against GWs |  | 1 | 1 |
| Would like to be vaccinated if vaccine also protect against genital warts | 1 |  | 1 |
| **Drivers for accepting HPV vaccination (attitudes & beliefs) (girls)** | **6** | **10** | **16** |
| Believes HPV vaccination will decrease cervical screening attendance |  | 1 | 1 |
| Do you believe your daughter will be fully protected against cervical cancer after HPV vaccination? |  | 1 | 1 |
| Do you think you are at risk of having an HPV infection? |  | 1 | 1 |
| Does HPV vaccine efficacy influence your decision? |  | 1 | 1 |
| HPV vaccination would be a good way to protect themselves against CC | 1 |  | 1 |
| HPV vaccination would be a good way to protect themselves against HPV | 1 |  | 1 |
| Knowledge of HPV causes cervical cancer and genital warts |  | 1 | 1 |
| Perception of vaccine efficacy: 95% | 1 |  | 1 |
| Reason for vaccination: prevention cancer |  | 1 | 1 |
| Reason for vaccination: prevention of a frequent infection |  | 1 | 1 |
| Reason to accept: vaccination offer the opportunity to preventing a severe and potentially fatal disease, namely CC |  | 1 | 1 |
| The most common topic was that the vaccine was a very good vaccine to prevent cancers | 1 |  | 1 |
| The vaccine was very good for preventing cancers | 1 |  | 1 |
| Wished the vaccine had been around when they themselves were young |  | 1 | 1 |
| Would be glad if the vaccination meant an end to smear tests |  | 1 | 1 |
| Would like to be vaccinated if vaccine also protect against genital warts | 1 |  | 1 |
| **Intention to HPV vaccination** | **2** | **3** | **5** |
| Agree to HPV vaccination for their child |  | 1 | 1 |
| In favour of vaccinating their future children | 1 |  | 1 |
| Vaccination: Now (YES) | 1 | 1 | 2 |
| Would you give your children HPV vaccination HPV? |  | 1 | 1 |
| **Intention to HPV vaccination (boys)** | **4** | **6** | **10** |
| Do you believe it is also necessary to vaccinate boys? |  | 1 | 1 |
| If you had a son, would you vaccinate him with HPV vaccine? |  | 1 | 1 |
| In favour of vaccinating boys | 1 |  | 1 |
| Intention to have the HPV vaccine | 1 |  | 1 |
| Intention to vaccinate 12-15 years old boy: Our son has already received the vaccine |  | 1 | 1 |
| Intention to vaccinate son |  | 1 | 1 |
| Intention to vaccinate sons |  | 1 | 1 |
| Would like to be vaccinated | 1 |  | 1 |
| Would you undergo HPV vaccination HPV? | 1 |  | 1 |
| Would you want your son to be vaccinated against HPV? |  | 1 | 1 |
| **Intention to HPV vaccination (girls)** | **9** | **15** | **24** |
| Accept an HPV vaccination | 1 |  | 1 |
| Attitude toward recommended vaccination | 1 |  | 1 |
| Do you believe it also is necessary to vaccinate girls? |  | 1 | 1 |
| HPV vaccination intention | 1 | 1 | 2 |
| I intent to vaccinate my daughter(s) in the future |  | 1 | 1 |
| I will have her vaccinated in a few weeks |  | 1 | 1 |
| I will vaccine my daughter(s) if she (they) asks me |  | 1 | 1 |
| Intended to accept HPV vaccination |  | 1 | 1 |
| Intention to have the HPV vaccine -girl- | 1 |  | 1 |
| Intention to vaccinate daughter |  | 2 | 2 |
| Parents with 0-11-year-old daughters wanted them to be vaccinated at 12 years |  | 1 | 1 |
| Probably + definitely accept the HPV vaccine for their daughter |  | 1 | 1 |
| The HPV vaccine was accepted |  | 1 | 1 |
| Will you accept the active offer of the HPV vaccine for your daughter? |  | 1 | 1 |
| Will you let your daughter(s) aged below X (the eligible age) receive HPV vaccination when she/they reach(es) the eligible age? |  | 1 | 1 |
| Would like to be vaccinated | 1 |  | 1 |
| Would you have your daughter vaccinated against HPV infection? |  | 1 | 1 |
| Would you have yourself vaccinated against HPV? Already been vaccinated | 1 |  | 1 |
| Would you have yourself vaccinated against HPV? Vaccination is a possibility | 1 |  | 1 |
| Would you have yourself vaccinated against HPV? Vaccination will occur | 1 |  | 1 |
| Would you undergo HPV vaccination HPV? | 1 |  | 1 |
| Would you vaccinate your daughter with HPV vaccine? |  | 1 | 1 |
| **Interested in receiving more information** | **1** |  | **1** |
| Would you be interested in receiving more information about HPV? | 1 |  | 1 |
| **Source of information- Family /friends** | **5** | **4** | **9** |
| Preferred source of information (Friends) | 1 |  | 1 |
| Preferred source of information (Parents) | 1 |  | 1 |
| Source of information: Friends | 1 | 1 | 2 |
| Source of information: Friends and relations |  | 1 | 1 |
| Source of information: Parents and family | 1 |  | 1 |
| Which do you think would be the best information source? family/friend | 1 |  | 1 |
| Which were the two main sources of information on HPV infection? Friends |  | 1 | 1 |
| Which were the two main sources of information on HPV infection? Parents, relatives |  | 1 | 1 |
| **Source of information- Internet** | **1** | **2** | **3** |
| Source of information: Internet | 1 | 1 | 2 |
| Which were the two main sources of information on HPV infection? Internet |  | 1 | 1 |
| **Source of information- Media** | **5** | **6** | **11** |
| Preferred source of information (Media) | 1 |  | 1 |
| Source of information: Magazines |  | 1 | 1 |
| Source of information: Mass media |  | 1 | 1 |
| Source of information: Newspapers |  | 1 | 1 |
| Source of information: Newspapers, magazines | 1 |  | 1 |
| Source of information: Radio | 1 | 1 | 2 |
| Source of information: TV | 1 | 1 | 2 |
| Which do you think would be the best information source? Media | 1 |  | 1 |
| Which were the two main sources of information on HPV infection? Newspapers, radio, TV |  | 1 | 1 |
| **Source of information- Medical staff** | **5** | **4** | **9** |
| Have you requested information from your GP about HPV infection and HPV vaccination? |  | 1 | 1 |
| Preferred source of information (Youth Clinic) | 1 |  | 1 |
| Source of information: Family physicians | 1 |  | 1 |
| Source of information: Specialists (paediatricians….) | 1 |  | 1 |
| Source of information: Healthcare provider |  | 1 | 1 |
| Source of information: Other healthcare professionals | 1 |  | 1 |
| Source of information: School nurse |  | 1 | 1 |
| Which do you think would be the best information source? Medical staff, in a private space | 1 |  | 1 |
| Which were the two main sources of information on HPV infection? Healthcare professionals |  | 1 | 1 |
| **Source of information- Other** | **1** |  | **1** |
| Preferred source of information (Other) | 1 |  | 1 |
| **Source of information- Printed documents** | **1** | **1** | **2** |
| Source of information: Leaflet in doctors’ surgery | 1 |  | 1 |
| Which were the two main sources of information on HPV infection? Books, magazines |  | 1 | 1 |
| **Source of information- School** | **3** | **2** | **5** |
| Preferred source of information (School nurse) | 1 |  | 1 |
| Should information be given in school? |  | 1 | 1 |
| Source of information: School (doctor, nurse, course) | 1 |  | 1 |
| Which do you think would be the best information source? School | 1 |  | 1 |
| Which were the two main sources of information on HPV infection? School |  | 1 | 1 |
| **Type of information received (negative)** |  | **3** | **3** |
| Advices concerning HPV vaccination received by consulted health professionals: Advice to delay |  | 1 | 1 |
| Advices concerning HPV vaccination received by consulted health professionals: Negative advice |  | 1 | 1 |
| Regarding the advice given by your doctor or nurse: They have advised that I do not give her the vaccine or that I defer giving it |  | 1 | 1 |
| **Type of information received (neutral/discordant)** |  | **5** | **5** |
| Advices concerning HPV vaccination received by consulted health professionals: Discordant advices |  | 1 | 1 |
| Advices concerning HPV vaccination received by consulted health professionals: No advice |  | 1 | 1 |
| Received discordant advices by the physician |  | 1 | 1 |
| Regarding the advice given by your doctor or nurse: I have not talked to them |  | 1 | 1 |
| Regarding the advice given by your doctor or nurse: They gave me information but left the decision about vaccination to me |  | 1 | 1 |
| **Type of information received (positive)** |  | **3** | **3** |
| Advices concerning HPV vaccination received by consulted health professionals: Positive advice |  | 1 | 1 |
| Received recommendation of HPV vaccination by the physician |  | 1 | 1 |
| Regarding the advice given by your doctor or nurse: They advise to me vaccinate |  | 1 | 1 |
| **Type of information received/needed** |  | **32** | **32** |
| Have you received information from your GP about HPV infection and HPV vaccination? Benefits of the vaccine |  | 1 | 1 |
| Have you received information from your GP about HPV infection and HPV vaccination? Direct skin contact in the genital area may be a medium of transmitting HPV |  | 1 | 1 |
| Have you received information from your GP about HPV infection and HPV vaccination? Genital warts are caused by the same types of HPV that cause cervical cancer |  | 1 | 1 |
| Have you received information from your GP about HPV infection and HPV vaccination? How is HPV transmitted? |  | 1 | 1 |
| Have you received information from your GP about HPV infection and HPV vaccination? HPV infection prevention methods |  | 1 | 1 |
| Have you received information from your GP about HPV infection and HPV vaccination? HPV vaccination recommendations |  | 1 | 1 |
| Have you received information from your GP about HPV infection and HPV vaccination? Nearly all cervical cancer cases are caused by HPV |  | 1 | 1 |
| Have you received information from your GP about HPV infection and HPV vaccination? Side effects of the vaccine |  | 1 | 1 |
| Have you received information from your GP about HPV infection and HPV vaccination? The existence of two vaccines |  | 1 | 1 |
| Have you received information from your GP about HPV infection and HPV vaccination? The known types of HPV are divided into those of high risk and low risk |  | 1 | 1 |
| Have you received information from your GP about HPV infection and HPV vaccination? Vaccine contraindications and precautions |  | 1 | 1 |
| Have you received information from your GP about HPV infection and HPV vaccination? What is HPV? |  | 1 | 1 |
| If given in school, what should it be? Biological information on HPV and other STIs. |  | 1 | 1 |
| If given in school, what should it be? Information of when and where to get treated for STIs. |  | 1 | 1 |
| If given in school, what should it be? Information on cervical cancer and its prevention |  | 1 | 1 |
| If given in school, what should it be? Safer sex messages. |  | 1 | 1 |
| If given in school, what should it be? Sexual abstinence messages |  | 1 | 1 |
| Which recommendation is significant to you when deciding on a vaccination for your children: Ministry of health |  | 2 | 2 |
| Which recommendation is significant to you when deciding on a vaccination for your children: other |  | 1 | 1 |
| Which recommendation is significant to you when deciding on a vaccination for your children: another physician |  | 2 | 2 |
| Which recommendation is significant to you when deciding on a vaccination for your children: paediatrician |  | 2 | 2 |
| Which recommendation is significant to you when deciding on a vaccination for your children: relative or friend |  | 2 | 2 |
| Which recommendation is significant to you when deciding on a vaccination for your children: religious authority |  | 2 | 2 |
| Which recommendation is significant to you when deciding on a vaccination for your children: teacher |  | 2 | 2 |
| Which recommendation is significant to you when deciding on a vaccination for your recommendation: regional body |  | 2 | 2 |
| **Who should decide** |  | **12** | **12** |
| Do children need information about the vaccine? |  | 1 | 1 |
| Do you agree that a well-informed child should be able to request vaccination at sexual health clinics without parental consent? (strongly agree/agree) |  | 1 | 1 |
| I don’t think my daughter is very capable to make her own decision about accepting the vaccination - concerns, organisational determinants and declining the HPV vaccination |  | 1 | 1 |
| I prefer that my son makes his own decision later |  | 1 | 1 |
| Parents reported discussing the vaccine with their daughters |  | 1 | 1 |
| Reasons for not informing their children about HPV infection: The parents make decisions for their children |  | 1 | 1 |
| Who should make the decision on vaccination? Father (or male guardian) |  | 1 | 1 |
| Who should make the decision on vaccination? Joint decision of parents and child |  | 1 | 1 |
| Who should make the decision on vaccination? Mother of female relative |  | 1 | 1 |
| Who should make the decision on vaccination? Only the parents |  | 1 | 1 |
| Who should make the decision on vaccination? The child |  | 1 | 1 |
| Willingness to vaccinate: Opinion child important |  | 1 | 1 |
| **Acceptability of vaccines in general** | **10** | **41** | **51** |
| **Agree to vaccinate at certain age** |  | **5** | **5** |
| Willingness to vaccinate: Age at vaccinations 0-3 |  | 1 | 1 |
| Willingness to vaccinate: Age at vaccinations 10-12 |  | 1 | 1 |
| Willingness to vaccinate: Age at vaccinations 13-18 |  | 1 | 1 |
| Willingness to vaccinate: Age at vaccinations 9 |  | 1 | 1 |
| Willingness to vaccinate: Girls and boys should be vaccinated |  | 1 | 1 |
| **Barriers to vaccination in general** | **3** | **20** | **23** |
| Against vaccinations |  | 1 | 1 |
| Child afraid of getting vaccinated at school |  | 1 | 1 |
| Child has allergy |  | 1 | 1 |
| Child is afraid of needles |  | 1 | 1 |
| Children received vaccine complications |  | 1 | 1 |
| Concern about safety of vaccines in general | 1 | 1 | 2 |
| Fear of debilitating/permanent side effects |  | 1 | 1 |
| Fear of side effects although temporary |  | 1 | 1 |
| Generally, against all vaccines |  | 1 | 1 |
| Giving their daughter too many vaccinations |  | 1 | 1 |
| I am afraid of needles | 1 |  | 1 |
| I am afraid of vaccinations for my children |  | 1 | 1 |
| I am against (too many) vaccines |  | 2 | 2 |
| I am concerned about vaccination side effects |  | 1 | 1 |
| I don’t believe/trust that the government would stop vaccinations if there was evidence of serious side effects - concerns, organisational determinants and declining the HPV vaccination |  | 1 | 1 |
| It’s not very important that my children receive all their vaccinations - concerns, organisational determinants and declining the HPV vaccination |  | 1 | 1 |
| Reason for not accept HPV vaccination: No confidence in vaccinations |  | 1 | 1 |
| Reason not to vaccinate: I don't believe in vaccination |  | 1 | 1 |
| Reason with the highest impact on your decision to get vaccinated: Not all vaccines are necessary | 1 |  | 1 |
| There are too many vaccinations already included in the childhood vaccination schedule |  | 1 | 1 |
| Vaccines aren’t effective in preventing diseases -behavioral determinants and declining HPV |  | 1 | 1 |
| **Barriers to vaccination in general (girls)** | **1** |  | **1** |
| Reasons for not vaccinate or undervaccinate: General vaccination beliefs | 1 |  | 1 |
| **Positive attitudes towards vaccination in general** | **6** | **16** | **22** |
| Acceptance of vaccination as a means to prevent diseases: Very in favour |  | 1 | 1 |
| Are you in favour of vaccination in general |  | 2 | 2 |
| Attitudes towards the childhood immunisation programme: Adherence (Yes) |  | 1 | 1 |
| Believed in the effectiveness of vaccination | 1 |  | 1 |
| Believes vaccines are efficient |  | 1 | 1 |
| Children received childhood vaccines |  | 1 | 1 |
| Compulsory vaccines were important | 1 |  | 1 |
| Do you agree that it is preferable to vaccinate every child (universal vaccination)? |  | 1 | 1 |
| I have a responsibility to have my children vaccinated for the protection of all children |  | 1 | 1 |
| I welcome all vaccines for children |  | 1 | 1 |
| I welcome any protection of my children against cancer |  | 1 | 1 |
| In general vaccinations are efficient | 1 |  | 1 |
| In general vaccinations are safe | 1 |  | 1 |
| It is very important that my children receive all their vaccinations |  | 1 | 1 |
| More kids should be vaccinated against diseases so that outbreaks do not occur |  | 1 | 1 |
| People who don’t have their kids vaccinated put others at risk |  | 1 | 1 |
| Positioned positively for vaccination | 1 |  | 1 |
| The government does a good job of protecting us from risks to health |  | 1 | 1 |
| Vaccination is one way that parents can make a positive contribution to their children’s health |  | 1 | 1 |
| Vaccinations were an effective way to prevent disease | 1 |  | 1 |
| What are the main reasons for wanting your son(s) to receive HPV vaccination? I welcome all vaccines |  | 1 | 1 |
| **Others** | **3** | **9** | **12** |
| **Agree to start sexual education at certain age** |  | **7** | **7** |
| At what age should sexual education begin? >15 |  | 1 | 1 |
| At what age should sexual education begin? ≤9 |  | 1 | 1 |
| At what age should sexual education begin? 11 |  | 1 | 1 |
| At what age should sexual education begin? 12 |  | 1 | 1 |
| At what age should sexual education begin? 13 |  | 1 | 1 |
| At what age should sexual education begin? 14 |  | 1 | 1 |
| At what age should sexual education begin?10 |  | 1 | 1 |
| **Others** | **3** | **2** | **5** |
| Had no taken yet the decision to vaccinate their daughter |  | 1 | 1 |
| I intend to attend Pap-smear testing (only girls) | 1 |  | 1 |
| I intend to use condom if I have sex with a new partner | 1 |  | 1 |
| If I have any concerns about vaccinations they are taken seriously by my doctor: agree |  | 1 | 1 |
| Speak with classmates or friends about the vaccine | 1 |  | 1 |
| **General total** | **154** | **336** | **490** |
